# Cascade biotransformation of estrogens by *Isaria fumosorosea* KCh J2

**DOI:** 10.1038/s41598-019-47225-1

**Published:** 2019-07-24

**Authors:** Ewa Kozłowska, Monika Dymarska, Edyta Kostrzewa-Susłow, Tomasz Janeczko

**Affiliations:** Department of Chemistry, Wrocław University of Environmental and Life Sciences, Norwida 25, 50-375 Wrocław, Poland

**Keywords:** Biocatalysis, Transferases, Oxidoreductases

## Abstract

Estrone, estradiol, ethynylestradiol and estrone 3-methyl ether underwent a biotransformation process in the submerged culture of *Isaria fumosorosea* KCh J2. Estrone was transformed into seven metabolites, four of which were glycosylated. Estradiol was selectively glycosylated at C-3 and then transformed to D-ring lactone. Ethynylestradiol was coupled with methylglucoside and 6β-hydroxyderivative was obtained. Estrone 3-methyl ether was not transformed indicating that a free hydroxyl group at C-3 is necessary for glycosylation. Baeyer–Villiger oxidation combined with hydroxylation and glycosylation was observed. All glycosides obtained in this study are 3-*O*-β-methylglucosides.

## Introduction

Estrogens are involved in the development and maintenance of the female phenotype, the maturation of reproductive cells and the development of pregnancy. They are also crucial for many other non-sexually sensitive processes, including growth, maturation of the nervous system, bone metabolism, remodelling and endothelial reactions^1–3^. In women, estrogens are mainly produced in the ovaries, smaller amounts in adrenal glands, and during pregnancy also in the placenta. The two main biologically active estrogens in non-pregnant women are estrone and estradiol. Estrone, depending on the assay used, is assigned 20–80% of estradiol bioactivity^4–6^. These compounds can be converted in mammalian cells into each other by 17β-hydroxysteroid dehydrogenases^7,8^.

Reduction of estrone to estradiol is also known as a result of using the whole cells of yeasts (*Saccharomyces cerevisiae*, *Saccharomyces carlsbergensis*, *Pichia fermentans*, *Rhodotorula glutinis*)^9,10^ or filamentous fungi (*Penicillium citrinum*)^10^. In filamentous fungal cultures, hydroxylation products at positions 6α, 6β, 15α are also described^11,12^, as well as simultaneous hydroxylation of estrone and carbonyl moiety reduction^11^ or hydroxylation with the oxidation of estradiol’s 17β-hydroxy group^12^. Many human CYP isoforms are involved in the oxidative metabolism of 17-estradiol and estrone. In the literature, hydroxylation products (mainly at the 2, 4, 6α, 7α, and 15α positions) obtained by 15 cytochrome P450 isoenzymes were described^13^.

Only in a few studies there were observed glycosides of steroid compounds formed as a result of biotransformation. Regioselective glucosylation of estrogen analogues mediated by the fungus *Rhizopus oryzae* AS 3.2380^14^ and hydroxylation with glucosylation by microalgae *Selenastrum capricornutum* strains^15^ were described. In our previous studies, we presented the *Isaria fumosorosea* KCh J2 strain, which provides glycosylation of flavonoid compounds^16–18^. Due to the structural similarity of some flavonoids to estrones, we decided to check whether the glucotransferase of this strain could effectively transform estrone. Moreover, this strain is an effective biocatalyst for hydroxylation and Baeyer–Villiger oxidation (BVO) of androstanes^19^. The aim of this study was testing the *Isaria fumosorosea* KCh J2 strain towards estrone’s transformation, and checking whether the products could be the effect of the combination of the reactions previously described for flavonoids (methylglucosylation) and androstanes (hydroxylation, BVO) or just one of them. Herein, we present products of microbial glycosylation, hydroxylation and Baeyer–Villiger oxidation of estrone, estradiol and ethynylestradiol.

## Materials and Methods

### Materials

The substrates, estrone (3-hydroxy-1,3,5(10)-estratrien-17-one) **(1)**, β-estradiol (3,17β-dihydroxy-1,3,5(10)-estratriene) **(2)**, 17α-ethynylestradiol (17α-ethynyl-1,3,5**(10)**-estratriene-3,17β-diol) **(3)**, estrone 3-methyl ether (3-methoxy-1,3,5**(10)**-estratrien-17-one) **(4)**, were purchased from Sigma-Aldrich. TLC and PTLC plates, deuterated NMR solvents and 5,7-dimethoxy-α-tetralone were purchased from Sigma-Aldrich. Archem supplied all other chemicals and reagents used. Solvents were of analytical grade.

The microorganism *Isaria fumosorosea* KCh J2 was obtained from the collection of the Department of Chemistry, Wrocław University of Environmental and Life Sciences (Wrocław, Poland). Isolation and identification procedures were described in our previous paper^16^. The strain was maintained on Sabouraud 4% dextrose-agar slopes and freshly subcultured before use in the transformation experiments.

### Screening procedure

Erlenmeyer flasks (300 mL), each containing 100 mL of the cultivation medium (3% glucose, 1% aminobac), were inoculated with a suspension of *I*. *fumosorosea* KCh J2 strain and then incubated for 3 days at 24 °C on a rotary shaker. Then 10 mg of a substrate dissolved in 1 mL of DMSO was added. Samples were taken on the 1st, 3rd, 7th and 10th day of the process and products were subsequently extracted using ethyl acetate and analysed using TLC and HPLC.

### Reaction course by NMR analysis

Erlenmeyer flasks with the cultivation medium and inoculum were incubated under the same conditions as in section “Screening procedure”. A solution of 10 mg of the substrate in DMSO was added to 3-day old cultures. Whole flasks were extracted with ethyl acetate after the 1st, 3rd, 7th and 10th day of transformation. Extracts were dried and concentrated *in vacuo*. The crude mixture was dissolved in DMSO-*d*_6_, and 5 mg of 5,7-dimethoxy-α-tetralone was added as an internal standard.

### Preparative biotransformation

The same transformations were performed on the preparative scale in 2000 mL flasks, each containing 500 mL of the cultivation medium. The culture of *I*. *fumosorosea* KCh J2 was incubated under the same conditions as in the screening procedure, and then 100 mg of substrate dissolved in 2 mL of DMSO was added to the 3-day-old culture. After the complete transformation of the substrate, the mixture was extracted with ethyl acetate (3 × 300 mL), dried (anhydrous MgSO_4_) and concentrated *in vacuo*. The crude mixture obtained this way was separated by preparative TLC and analysed (TLC, HPLC).

### Analytical methods

The course of the biotransformation was monitored using TLC. The composition of product mixtures was established by ^1^HNMR. The crude mixture was separated as described previously by preparative TLC (Silica Gel GF, 500 μm) and chloroform/methanol mixture (9:1, v/v) as an eluent^19–21^. After elution products were detected under UV light (365 nm) then scraped from the plate and eluted with ethyl acetate to give pure fractions. Analytical TLC was carried out on silica gel G. Compounds were detected by spraying the plates with a H_2_SO_4_/CH_3_OH mixture (1:1, v/v) and visualised under UV light (254 nm). HPLC analyses were performed with a Waters 2690 instrument equipped with a Waters 996 photodiode array detector, using an ODS 2 column (4.6 × 250 mm) and a Guard- Pak Inserts μBondapak C18 pre-column. Separation conditions were as follows: gradient elution, using 80% of acetonitrile in 4.5% acetic acid solution (eluent A) and 4.5% acetic acid (eluent B); flow, 1 mL/min; detection wavelength 280 nm; program: 0–7 min, 10% A 90% B; 7–10 min, 50% A 50% B; 10–13 min, 60% A 40% B; 13–15 min, 70% A 30% B; 15–20 min 80% A The NMR spectra were recorded on a DRX 600 MHz spectrometer (Bruker, Bruker, Billerica, MA, USA) and measured in CDCl_3_. Products poorly soluble in chloroform were dissolved in DMSO-*d*_6_. The products’ structures were determined by means of elemental analysis, ^1^HNMR, ^13^CNMR (Table 1) and correlation spectroscopy (HMBC, HMQC).

**Table 1:** ^13^C NMR chemical shifts of products in DMSO-*d*_6_ or CDCl_3_ (*).

Atom No	Products														
	5	6	7	8	8*	9	9*	10	10*	11	12	13	14	15	16
1	125.69	125.67	125.91	126.11	126.67	126.23	126.69	125.00	125.59	112.76	126.06	124.86	125.00	125.62	126.18
2	113.71	113.70	113.39	113.78	114.10	113.89	114.26	114.68	114.92	143.24	113.69	114.61	114.64	114.38	113.83
3	155.32	155.21	155.42	155.27	154.96	155.37	155.12	156.47	156.13	144.73	155.16	156.06	156.24	155.07	155.24
4	113.78	113.73	113.94	116.32	116.88	115.81	116.65	116.09	116.65	117.37	116.25	116.01	116.01	116.23	116.30
5	142.13	142.12	141.96	137.36	138.25	137.25	137.94	137.12	137.49	126.77	137.37	137.08	137.22	140.12	137.44
6	67.96	68.10	68.11	29.19	29.77	29.45	29.98	29.63	30.12	28.53	29.29	30.06	29.42	65.56	29.35
7	36.82	37.72	36.15	26.05	26.56	25.53	26.15	27.02	27.87	26.28	26.86	27.87	27.27	36.53	26.95
8	37.14	37.93	39.75	37.84	38.34	40.35	41.39	40.23	40.84	37.76	38.56	38.53	37.63	33.30	39.12
9	44.10	44.23	42.85	43.51	44.14	42.23	42.91	127.53	129.10	43.63	43.60	128.16	127.73	43.37	43.45
10	129.48	130.03	129.05	133.09	134.67	132.70	133.97	134.38	134.64	134.19	133.59	134.47	134.96	130.36	133.52
11	25.56	26.10	27.21	25.51	25.99	27.16	27.54	115.64	116.41	25.55	26.04	118.23	116.95	25.82	26.12
12	31.38	36.60	39.02	31.38	31.67	40.05	39.36	40.75	40.84	31.40	36.89	38.53	33.66	32.64	32.65
13	47.29	42.80	82.75	47.36	48.11	82.81	83.43	81.85	82.69	47.34	42.83	41.06	45.48	46.67	46.74
14	49.09	49.04	43.95	49.60	50.52	44.42	45.28	41.64	42.62	49.61	49.55	47.00	47.00	48.75	49.09
15	21.12	22.79	19.16	21.17	21.71	19.97	19.85	19.97	20.54	21.16	22.81	23.68	22.08	22.49	22.53
16	35.39	29.90	28.25	35.41	35.99	28.27	28.71	29.03	29.48	35.42	29.62	29.65	35.76	38.85	38.85
17	219.66	80.05	170.79	219.74	221.06	170.12	171.56	170.75	170.37	219.74	80.07	80.16	220.41	78.22	78.23
18	13.54	11.29	19.96	13.55	13.96	20.31	20.25	20.31	20.73	13.53	11.28	11.41	14.29	12.84	12.79
1c				100.34	100.50	100.34	100.44	100.21	100.33	102.52	100.34	100.29	100.24		100.40
2c				73.49	73.88	73.49	73.89	73.49	73.87	73.60	73.49	73.53	73.49		73.54
3c				76.35	76.45	76.36	76.47	76.32	76.47	75.75	76.35	76.34	76.31		76.39
4c				79.08	79.12	79.10	79.14	79.06	79.11	79.16	79.07	79.10	79.05		79.14
5c				75.60	75.61	75.61	75.67	75.65	75.73	75.62	75.59	75.65	75.63		75.63
6c				60.31	62.10	60.31	62.16	60.28	62.16	60.36	60.30	60.32	60.28		60.36
-O*C*H_3_				59.67	60.96	59.68	61.06	59.68	61.06	59.70	59.66	59.71	59.66		59.71
20														89.06	89.01
21														75.08	75.11

## Results and Discussion

### Spectral data of isolated metabolites

#### 6β-hydroxyestrone (**5**)

^1^H NMR (600 MHz) (ppm) (DMSO-*d*_6_) δ: 0.82 (s, 3H, 18-H); 1.27 (dd, 1H, *J* = 12.0 Hz, 7-Hα); 1.28–1.37 (m, 2H, 11-Hβ, 12-Hα); 1.45–1.61 (m, 3H, 8-H, 14-H, 15-Hβ); 1.71 (m, 1H, 12-Hβ); 1.90–1.95 (m, 1H, 15-Hα); 2.03 (dd, 1H, *J* = 18.8, 8.8 Hz, 16-Hα); 2.09 (ddd, 1H, *J* = 8.6, 6.2, 1.9 Hz, 7-Hβ); 2.15–2.20 (m, 1H, 9-H); 2.23–2.28 (m, 1H, 11-Hα); 2.40 (dd, 1H, *J* = 18.8, 8.1 Hz, 16-Hβ); 4.53–4.58 (m, 1H, 6-Hα); 5.15 (d, 1H, *J* = 7.1 Hz, C6-OH); 6.54 (dd, 1H, *J* = 8.4, 2.6 Hz, 2-H); 6.92 (dd, 1H, *J* = 2.6, 0.5 Hz, 4-H); 7.00 (d, 1H, *J* = 8.4 Hz, 1-H); 9.04 (s, 1H, C3-OH).

#### estra-3,6β,17β-triol (**6**)

^1^H NMR (600 MHz) (ppm) (DMSO-*d*_6_) δ: 0.65 (s, 3H, 18-H); 1.14–1.28 (m, 5H, 7-Hα, 11-Hβ, 12-Hα, 14-H, 15-Hβ); 1.33–1.42 (m, 2H, 8-H, 16-Hα); 1.53–1.60 (m, 1H, 15-Hα); 1.81 (dt, 1H, *J* = 12.3, 3.1 Hz, 12-Hβ); 1.87 (dtd, 1H, *J* = 13.3, 9.3, 5.7 Hz, 16-Hβ); 1.98 (ddd, 1H, *J* = 11.8, 6.1, 1.8 Hz, 7-Hβ); 2.12 (td, 1H, *J* = 11.1, 4.0 Hz, 9-H); 2.20 (dq, 1H, *J* = 13.2, 3.8 Hz, 11-Hα); 3.51 (td, 1H, *J* = 8.6, 4.7 Hz, 17-Hα); 4.50 (d, 1H, *J* = 4.7 Hz, C17-OH); 4.50–4.55 (m, 1H, 6-Hα); 5.10 (d, 1H, *J* = 7.0 Hz, C6-OH); 6.55 (dd, 1H, *J* = 8.4, 2.6 Hz, 2-H); 6.92 (d, 1H, *J* = 2.6 Hz, 4-H); 7.01 (d, 1H, *J* = 8.3 Hz, 1-H); 9.03 (s, 1H, C3-OH).

#### 3,6β-dihydroxy-17a-oxa-D-homo-estrone (**7**)

^1^H NMR (600 MHz) (ppm) (DMSO-*d*_6_) δ: 1.19–1.26 (m, 3H, 7-Hα, 8-H, 11-Hβ); 1.27 (s, 3H, 18-H); 1.51 (tt, 1H, *J* = 13.1, 8.5 Hz, 15-Hβ); 1.61 (ddd, 1H, *J* = 13.2, 10.4, 4.4 Hz, 14-H); 1.73 (td, 1H, *J* = 13.1, 4.2 Hz, 12-Hα); 1.91 (dt, 1H, *J* = 12.4, 3.1 Hz, 12-Hβ); 1.94–1.99 (m, 1H, 15-Hα); 2.16 (dd, 1H, *J* = 10.4, 6.2 Hz, 7-Hβ); 2.34–2.41 (m, 2H, 9-H, 11-Hα); 2.50–2.55 (m, 1H, 16-Hα); 2.66 (ddd, 1H, *J* = 18.5, 8.8, 2.0 Hz, 16-Hβ); 4.49–4.55 (m, 1H, 6-Hα); 5.21 (d, 1H, *J* = 7.1 Hz, C6-OH); 6.57 (dd, 1H, *J* = 8.4, 2.6 Hz, 2-H); 6.92 (d, 1H, *J* = 2.3 Hz, 4-H); 7.04 (d, 1H, *J* = 8.5 Hz, 1-H); 9.10 (s, 1H, C3-OH).

#### 3-(β-D-4′-*O*-methyloglucosyloxy)-estrone (**8**)

^1^H NMR (600 MHz) (ppm) (CDCl_3_) δ: 0.90 (s, 3H, 18-H); 1.38–1.53 (m, 4H, 7-Hα, 11-Hβ, 12-Hα, 14-H); 1.57 (ddd, 1H, *J* = 13.0, 10.9, 2.7 Hz, 8-H); 1.62 (tt, 1H, *J* = 12.3, 9.0 Hz, 15-Hβ); 1.86–1.90 (m, 1H, 12-Hβ); 1.93 (d, 1 H, *J* = 12.5, 2.7 Hz, 7-Hβ); 1.98 (s, 1 H *J* = 13.0, 9.8, 6.7 Hz, 15-Hα); 2.07 (dt, 1 H, *J* = 19.1, 9.1 Hz, 16-Hα); 2.17 (td, 1 H, *J* = 10.5, 4.0 Hz, 9-H); 2.31 (dq, 1H, *J* = 13.1, 4.3 Hz, 11-Hα); 2.43 (dd, 1H, *J* = 19.1, 8.6 Hz, 16-Hβ); 2.79–2.86 (m, 2H, 6-Hα, 6-Hβ); 3.22 (t, 1H, *J* = 9.3 Hz, 4-H′); 3.37 (ddd, 1H, *J* = 9.6, 4.7, 2.8 Hz, 5-H′); 3.54 (s, 3H, -OCH_3_); 3.56 (dd, 1H, *J* = 9.4, 8.0 Hz, 2-H′); 3.65 (t, 1H, *J* = 9.1 Hz, 3-H′); 3.69 (dd, 1H, *J* = 12.0, 4.7 Hz, one of 6-H′); 3.85 (dd, 1H, *J* = 12.0, 2.6 Hz, one of 6-H′); 4.82 (d, 1H, *J* = 7.7 Hz, 1-H′); 6.68 (d, 1H, *J* = 2.6 Hz, 4-H); 6.74 (dd, 1H, *J* = 8.6, 2.6 Hz, 2-H); 7.14 (d, 1H, *J* = 8.6 Hz, 1-H).

^1^H NMR (600 MHz) (ppm) (DMSO-*d*_6_) δ: 0.84 (s, 3H, 18-H); 1.30–1.42 (m, 3H, 11-Hβ, 12-Hα, 14-H); 1.45–1.53 (m, 2H, 7-Hα, 8-H); 1.56 (tt, 1H, *J* = 11.9, 9.0 Hz, 15-Hα); 1.74–1.77 (m, 1H, 12-Hβ); 1.90–1.98 (m, 2H, 7-Hβ, 15-Hα); 2.05 (dd, 1H, *J* = 18.8, 9.1 Hz, 16-Hα); 2.15–2.21 (m, 1H, 9-H); 2.31–2.37 (m, 1H, 11-Hα); 2.45 (dd, 1H, *J* = 19.0, 8.3 Hz, 16-Hβ); 2.79–2.87 (m, 2H, 6-Hα, 6-Hβ); 3.03 (t, 1H, *J* = 9.4 Hz, 4-H′); 3.22 (ddd, 1H, *J* = 8.6, 8.1, 5.2 Hz, 5-H′); 3.33 (ddd, 1H, *J* = 9.8, 4.9, 2.0 Hz, 2-H′); 3.36 (s, 3H, -OCH_3_); 3.40 (td, 1H, *J* = 9.0, 5.4 Hz, 3-H′); 3.50 (ddd, 1H, *J* = 11.6, 6.3, 5.2 Hz, one of 6-H′); 3.63 (ddd, 1H, *J* = 11.7, 4.8, 1.7 Hz, one of 6-H′); 4.68 (dd, 1H, *J* = 6.3, 5.1 Hz, C′6-OH); 4.81 (d, 1H, *J* = 7.8 Hz, 1-H′); 5.23 (d, 1H, *J* = 5.4 Hz, C′3-OH); 5.33 (d, 1H, *J* = 5.3 Hz, C′2-OH); 6.73 (d, 1H, *J* = 2.6 Hz, 4-H); 6.80 (dd, 1H, *J* = 8.6, 2.6 Hz, 2-H); 7.18 (d, 1H, *J* = 8.6 Hz, 1-H).

#### 3-(β-D-4′-*O*-methyloglucosyloxy)-17a-oxa-D-homo-estr-17-one (**9**)

^1^H NMR (600 MHz) (ppm) (CDCl_3_) δ: 1.21–1.25 (m, 1H, 8-H); 1.26 (s, 3H, 18-H); 1.36–1.44 (m, 2H, 7-Hα, 11-Hβ); 1.60–1.67 (m, 2H, 14-H, 15-Hβ); 1.88 (td, 1H, *J* = 13.6, 3.9 Hz, 12-Hα); 2.04–2.13 (m, 3H, 7-Hβ, 12-Hβ, 15-Hα); 2.42–2.51 (m, 2H, 9-H, 11-Hα); 2.63 (dt, 1H, *J* = 19.0, 9.1 Hz, 16-Hα); 2.73 (dd, 1H, *J* = 19.0, 8.8 Hz, 16-Hβ); 2.84–2.90 (m, 2H, 6-Hα, 6-Hβ); 3.30 (t, 1H, *J* = 9.3 Hz, 4-H′); 3.45 (ddd, 1H, *J* = 9.6, 4.7, 2.8 Hz, 5-H′); 3.62 (s, 3H, -OCH_3_); 3.63 (dd, 1H, *J* = 9.4, 7.8 Hz, 2-H′); 3.73 (t, 1H, *J* = 9.1 Hz, 3-H′); 3.73–3.79 (m 1H, one of 6-H′); 3.93 (dd, 1H, *J* = 12.0, 2.6 Hz, one of 6-H′); 4.90 (d, *J* = 7.9 Hz, 1-H′); 6.75 (d, 1H, *J* = 2.6 Hz, 4-H); 6.75 (d, *J* = 2.6 Hz, 1H); 6.83 (dd, 1H, *J* = 8.6, 2.8 Hz, 2-H); 7.20 (d, 1H, *J* = 8.5 Hz, 1-H).

^1^H NMR (600 MHz) (ppm) (DMSO-*d*_6_) δ: 1.27 (s, 3H, 18-H); 3.44 (s, 3H, -OCH_3_); 4.79 (d, *J* = 7.8 Hz, 1-H′); 6.71 (d, 1H, *J* = 2.6 Hz, 4-H); 6.79 (dd, 1H, *J* = 8.7, 2.7 Hz, 2-H); 7.19 (d, 1H, *J* = 8.9 Hz, 1-H).

#### 3-(β-D-4′-*O*-methyloglucosyloxy)-17a-oxa-D-homo-estr-9-en-17-one (**10**)

^1^H NMR (600 MHz) (ppm) (CDCl_3_) δ: 1.26 (s, 3H, 18-H); 1.36–1.40 (m, 2H, 7-Hα); 1.68–1.73 (m, 1H, 15-Hβ); 1.75 (ddd, 1H, *J* = 12.7, 9.9, 3.5 Hz, 14-H); 2.01 (dd, 1H, *J* = 12.0, 2.7 Hz, 8-H); 2.14–2.25 (m, 2H, 7-Hβ, 15-Hα); 2.48 (td, 1H, *J* = 13.6, 3.9 Hz, 12-Hα); 2.55–2.62 (m, 2H, 12-Hβ, 16-Hα); 2.75 (dd, 1H, *J* = 19.0, 8.8 Hz, 16-Hβ); (m, 2H, 6-Hα, 6-Hβ); 3.30 (t, 1H, *J* = 9.3 Hz, 4-H′); 3.46 (ddd, 1H, *J* = 9.6, 4.7, 2.8 Hz, 5-H′); 3.62 (s, 3H, -OCH_3_); 3.64 (dd, 1H, *J* = 9.3, 7.6 Hz, 2-H′); 3.74 (t, 1H, *J* = 9.1 Hz, 3-H′); 3.73–3.79 (m 1H, one of 6-H′); 3.94 (dd, 1H, *J* = 12.0, 2.6 Hz, one of 6-H′); 4.91 (d, 1H, *J* = 7.9 Hz, 1-H′); 6.07–6.10 (m, 1H, 11-H); 6.76 (d, 1H, *J* = 2.6 Hz, 4-H); 6.83 (dd, 1H, *J* = 8.8, 2.9 Hz, 2-H); 7.49 (d, 1H, *J* = 8.8 Hz, 1-H).

#### 3-*O*-(β-D-4′-*O*-methyloglucopyranosyl)-2-hydroxyestrone (**11**)

^1^H NMR (600 MHz) (ppm) (DMSO-*d*_6_) δ: 0.82 (s, 3H, 18-H); 1.24–1.37 (m, 3H, 7-Hα, 11-Hβ, 12-Hα); 1.43–1.49 (m, 2H, 8-H, 14-H); 1.55 (tt, 1H, *J* = 12.3, 8.9 Hz, 15-Hα); 1.74 (dd, 1H, *J* = 8.8, 2.3 Hz, 12-Hβ); 1.88–1.97 (m, 2H, 7-Hβ, 15-Hα); 2.05 (dd, 1H, *J* = 18.8, 9.1 Hz, 16-Hα); 2.12–2.17 (m, 1H, 9-H); 2.21–2.28 (m, 1H, 11-Hα); 2.43 (dd, 1H, *J* = 18.8, 8.7 Hz, 16-Hβ); 2.67–2.72 (m, 2H, 6-Hα, 6-Hβ); 3.02 (t, 1H, *J* = 9.4 Hz, 4-H′); 3.24–3.29 (m, 1H, 5-H′); 3.31 (ddd, 1H, *J* = 10.1, 5.1, 1.9 Hz, 2-H′); 3.39–3.42 (m, 1H, 3-H′); 3.45 (s, 3H, -OCH_3_); 3.48–3.53 (m, 1H, one of 6-H′); 3.64 (ddd, 1H, *J* = 11.9, 4.4, 1.5 Hz, one of 6-H′); 4.58 (d, 1H, *J* = 7.8 Hz, 1-H′); 4.73 (t, 1H, *J* = 5.5 Hz, C′6-OH); 5.24 (d, 1H, *J* = 5.3 Hz, C′3-OH); 5.59 (d, 1H, *J* = 3.0 Hz, C′2-OH); 6.70 (s, 1H, 1-H); 6.78 (s, 1H, 4-H).

^1^H NMR (600 MHz) (ppm) (CDCl_3_) δ: 1.28 (s, 3H, 18-H); 3.45 (s, 3H, -OCH_3_); 4.82 (d, 1H, *J* = 7.8 Hz, 1-H′); 6.10–6.12 (m, 1H, 11-H); 6.74 (d, 1H, *J* = 2.6 Hz, 4-H); 6.81 (dd, 1H, *J* = 8.8, 2.6 Hz, 2-H); 7.52 (d, 1H, *J* = 8.9 Hz, 1-H).

#### 3-(β-D-4′-*O*-methyloglucosyloxy)-estr-17β-ol (**12**)

^1^H NMR (600 MHz) (ppm) (DMSO-*d*_6_) δ: 0.66 (s, 3H, 18-H); 1.12 (ddd, 1H, *J* = 18.8, 11.3, 7.5 Hz, 14-H); 1.19 (td, 1H, *J* = 12.9, 3.8 Hz, 12-Hα); 1.22–1.39 (m, 5H, 7-Hα, 8-H, 11-Hβ, 15-Hβ, 16-Hα); 1.55–1.62 (m, 1H, 15-Hα); 1.79 (ddt, 1H, *J* = 9.6, 4.6, 2.8 Hz, 7-Hβ); 1.84 (dt, 1H, *J* = 9.6, 3.2 Hz, 12-Hβ); 1.86–1.92 (m, 1H, 16-Hβ); 2.14 (td, 1H, *J* = 16.0, 5.3 Hz, 9-H); 2.27 (dq, 1H, *J* = 13.5, 3.2 Hz, 11-Hα); 2.73–2.77 (m, 2H, 6-Hα, 6-Hβ); 3.01 (t, 1H, *J* = 9.4 Hz, 4-H′); 3.18–3.23 (m, 1H, 2-H′); 3.30–3.40 (m, 2H, 3-H′, 5-H′); 3.44 (s, 3H, -OCH_3_); 3.45–3.55 (m, 2H, one of 6-H′ and 17-Hα); 3.61 (ddd, 1H, *J* = 11.7, 4.8, 1.7 Hz, one of 6-H′); 4.49 (d, 1H, *J* = 4.9 Hz, C17-OH); 4.66 (dd, 1H, *J* = 6.4, 5.1 Hz, C′6-OH); 4.78 (d, 1H, *J* = 7.8 Hz, 1-H′); 5.20 (d, 1H, *J* = 5.5 Hz, C′3-OH); 5.31 (d, 1H, *J* = 5.3 Hz, C′2-OH); 6.69 (d, 1H, *J* = 2.6 Hz, 4-H); 6.76 (dd, 1H, *J* = 8.4, 2.6 Hz, 2-H); 7.16 (d, 1H, *J* = 8.6 Hz, 1-H).

#### 3-(β-D-4′-*O*-methyloglucosyloxy)-estr-9-en-17β-ol (**13**)

^1^H NMR (600 MHz) (ppm) (DMSO-*d*_6_) δ: 0.67 (s, 3H, 18-H); 1.39–1.47 (m, 3H, 7-Hα, 14-H, 15-Hβ); 1.39–1.47 (m, 1H, 16-Hα); 1.71–1.76 (m, 1H, 15-Hα); 1.92–2.05 (m, 4H, 7-Hβ, 8-H, 12-Hβ, 16-Hβ); 2.15 (dd, 1H, *J* = 16.3, 5.6 Hz, 12-Hβ); 2.74–2.77 (m, 2H, 6-Hα, 6-Hβ); 3.02 (t, 1H, *J* = 9.4 Hz, 4-H′); 3.21 (ddd, 1H, *J* = 9.8, 4.7, 2.0 Hz, 2-H′); 3.31–3.40 (m, 2H, 3-H′and 5-H′); 3.45 (s, 3H, -OCH_3_); 3.47–3.52 (m, 2H, one of 6-H′ and 17α-H); 3.59–3.64 (m, 1H, one of 6-H′); 4.58 (d, 1H, *J* = 4.9 Hz, C17-OH); 4.68 (dd, 1H, *J* = 6.2, 5.3 Hz, C′6-OH); 4.81 (d, 1H, *J* = 7.8 Hz, 1-H′); 5.22 (d, 1H, *J* = 5.5 Hz, C′3-OH); 5.33 (d, 1H, *J* = 5.2 Hz, C′2-OH); 6.14–6.16 (m, 1H, 11-H); 6.71 (d, 1H, *J* = 2.5 Hz, 4-H); 6.78 (dd, 1H, *J* = 8.6, 2.6 Hz, 2-H); 7.53 (d, 1H, *J* = 8.9 Hz, 1-H).

#### 3-(β-D-4′-*O*-methyloglucosyloxy)-estr-9-en-17-on (**14**)

^1^H NMR (600 MHz) (ppm) (DMSO-*d*_6_) δ: 0.83 (s, 3H, 18-H); 1.25–1.29 (m, 1H, 7-Hα); 1.57–1.64 (m, 2H, 14-H, 15-Hα); 2.03–2.10 (m, 4H, 7-Hβ, 12-Hα, 15-Hα, 16-Hα); 2.14–2.21 (m, 2H, 8-H, 12-Hβ); 2.46 (dd, 1H,*J* = 18.8, 8.7 Hz, 16-Hβ); 2.78–2.83 (m, 2H, 6-Hα, 6-Hβ);

3.02 (t, 1H, *J* = 9.3 Hz, 4-H′); 3.21 (ddd, 1H, *J* = 9.6, 4.7, 2.8 Hz, 5-H′); 3.33 (ddd, 1H, *J* = 9.8, 4.7, 2.0 Hz, 2-H′); 3.39–3.43 (m, 1H, 3-H′); 3.45 (s, 3H, -OCH_3_); 3.47–3.53 (m, 1H, one of 6-H′); 3.63 (ddd, 1H, *J* = 11.9, 4.7, 1.6 Hz, one of 6-H′); 4.70 (dd, 1H, *J* = 6.2, 5.3 Hz, C′6-OH); 4.82 (d, 1H, *J* = 7.8 Hz, 1-H′); 5.23 (d, 1H, *J* = 5.6 Hz, C′3-OH); 5.34 (d, 1H, *J* = 5.2 Hz, C′2-OH); 6.15–6.18 (m, 1H, 11-H); 6.75 (d, 1H, *J* = 2.5 Hz, 4-H); 6.80 (dd, 1H, *J* = 9.1, 2.6 Hz, 2-H); 7.54 (d, 1H, *J* = 9.0 Hz, 1-H).

#### 17-ethynyloestra-3,6β,17β-triol (**15**)

^1^H NMR (600 MHz) (ppm) (DMSO-*d*_6_) δ: 0.76 (s, 3H, 18-H); 1.27–1.35 (m, 2H, 11-Hβ, 15-Hβ); 1.39 (td, *J* = 11.9, 4.3 Hz, 7-Hα); 1.61–1.70 (m, 4H, 8-H, 12-Hα, 14-H, 15-Hα); 1.74–1.80 (m, 2H, 7-Hβ, 12-Hβ); 1.83–1.88 (m, 1H, 16-Hα); 1.93 (td, 1H, *J* = 11.4, 3.9 Hz, 9-H); 2.07–2.13 (m, 1H, 16-Hβ); 2.23–2.31 (m, 1H, 11-Hα); 3.32 (s, 1H, 21-H); 4.45–4.58 (m, 1H, 6-Hα); 4.96 (d, 1H, *J* = 5.5 Hz, C6-OH); 5.33 (s, 1H, C17-OH); 6.59 (dd, 1H, *J* = 8.4, 2.7 Hz, 2-H); 6.70 (d, 1H, *J* = 2.7 Hz, 4-H); 7.06 (d, 1H, *J* = 8.4 Hz, 1-H); 9.07 (s, 1H, C3-OH).

#### 3-(β-D-4′-*O*-methyloglucosyloxy)-17-ethynyloestr-17β-ol (**16**)

^1^H NMR (600 MHz) (ppm) (DMSO-*d*_6_) δ: 0.75 (s, 3H, 18-H); 1.22–1.35 (m, 4H, 7-Hα, 8-H, 11-Hβ, 15-Hβ); 1.59 (dd, 1H, *J* = 10.9, 7.7 Hz, 14-H); 1.62–1.68 (m, 2H, 12-Hα, 15-Hα); 1.75–1.82 (m, 2H, 7-Hβ, 12-Hβ); 1.86 (td, 1H, *J* = 13.1, 3.5 Hz, 16-Hα); 2.06–2.13 (m, 2H, 9-H, 16-Hβ); 2.32 (dq, 1H, *J* = 13.5, 3.0 Hz, 11-Hα); 2.72–2.78 (m, 2H, 6-Hα, 6-Hβ); 3.01 (t, 1H, *J* = 9.3 Hz, 4-H′); 3.20 (ddd, 1H, *J* = 8.8, 8.0, 5.2 Hz, 2-H′); 3.30 (ddd, 1 H *J* = 9.6, 4.9, 2.0 Hz, 5-H′); 3.36–3.39 (m, 1H, 3-H′); 3.44 (s, 3H, -OCH_3_); 3.45–3.51 (m, 1H, one of 6-H′); 3.61 (ddd, 1H, *J* = 11.9, 4.7, 1.6 Hz, one of 6-H′); 4.68 (t, 1H, *J* = 5.7 Hz, C′6-OH); 4.78 (d, 1H, *J* = 6.7 Hz, 1-H′); 5.21 (d, 1H, *J* = 5.4 Hz, C′3-OH); 5.32 (d, 1H, *J* = 5.3 Hz, C′2-OH); 5.34 (s, 1H, C17-OH); 6.69 (d, 1H, *J* = 2.6 Hz, 4-H); 6.77 (dd, 1H, *J* = 8.6, 2.6 Hz, 2-H); 7.16 (d, 1H, *J* = 8.7 Hz, 1-H).

**Figure 1:**
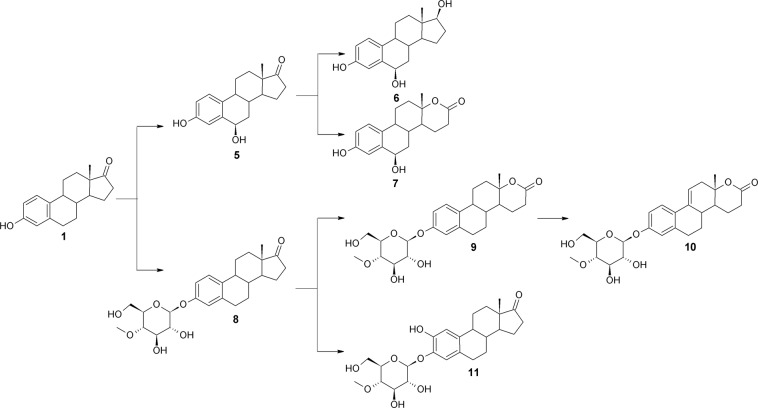
The proposed course of transformation of estrone **(1)** in the culture of *Isaria fumosorosea* KCh J2.

Transformation of estrone **(1)** in the culture of *Isaria fumosorosea* KCh J2 led to seven metabolites. Four of them were obtained as methyloglucosyl derivatives (Fig. 1). Substrate **1** was hydroxylated at the 6β position to compound **5**. Then the C-17 carbonyl group of **5** was reduced, giving compound **6**. Estradiol was not observed in the reaction mixture, which disproves the possibility of reducing estrone to estradiol and then hydroxylating it at the 6β position. It can be assumed that the dehydrogenases reducing the carbonyl group at C-17 accepted only 6β-hydroxy derivative. Simultaneously, the C-17 carbonyl group of **5** is necessary for Baeyer-Villiger oxidation of the D ring, which led to **7**. Apparently the hydroxyl group at the 6β position has to be a steric hindrance for glycosylation because none of the 6β-hydroxylated derivatives was conjugated with a glycosyl moiety. The substrate **(1)** was glycosylated at the C-3 hydroxyl group, giving compound **8**, which was transformed further to D-ring lactone **9**. The tested strain can introduce a double bond between C-9 and C-11 in the obtained lactone, forming C-9 unsaturated D-lactone **10**. Together with that transformation, hydroxylation of **8** at the C-2 position occurred, giving product **11**. No free 2-hydroxyestrone and no conjugated 6β-hydroxy-derivatives were detected, which suggests that the methylglucosyl moiety is a steric hindrance for 6β-steroid hydroxylase but not for 2-steroid hydroxylase. Furthermore, Baeyer-Villiger oxidation combined with the previous hydroxylation is not common for microbial steroid transformation. Such a combination of reactions is possible in *Beauveria bassiana*^22,23^ and *Isaria fumosorosea*^19,24^ culture.

**Figure 2:**
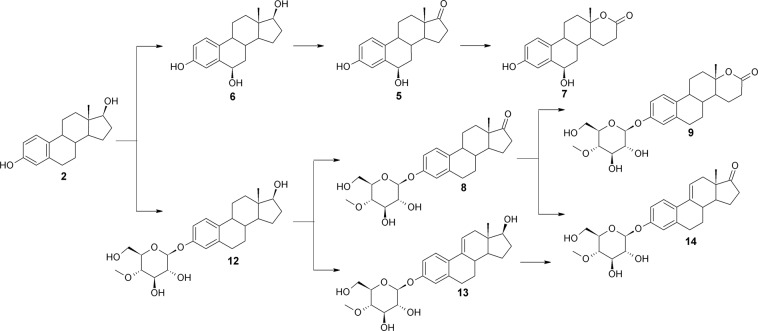
Putative transformation of estradiol **(2)** in the culture of *Isaria fumosorosea* KCh J2.

Transformation of estradiol **(2)**, estrane with two free hydroxyl groups at the C-3 and C-17β position, was performed to evaluate regioselectivity of glycosylation. As in the case of estrone **(1)**, free 6β-hydroxy derivatives were obtained. Lactone **7** was a result of 6β-hydroxylation of **2**, then oxidation of the C-17β hydroxyl group to **5** and then Baeyer-Villiger oxidation (Fig. 2). Simultaneously, regioselective glycosylation to **12** occurred at the C-3 position of **2**. Then, the C-17β hydroxyl group of conjugated **12** was oxidised twice – to compound **8** and then to **9**. At the same time, between C-9 and C-11 of **12** a double bond was introduced, forming **13**, then compound **13** was oxidized to give **14**. Probably, compound **14** can be formed from **8** too. Similar to estrone **(1)**, *Isaria fumosorosea* KCh J2 is not able to glycosylate 6β-hydroxyderivatives of estradiol **(2)**. This substrate is metabolized faster than estrone **(1)**, but emerging products are in nearly equal concentrations.

**Figure 3:**
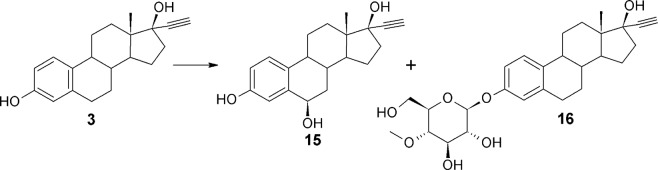
Transformation of ethynylestradiol **(3)** in the culture of *Isaria fumosorosea* KCh J2.

The multitude of emerging products of estrone **(1)** and estradiol **(2)** is the result of glycosylation and transformations in the D-ring of those compounds. Therefore ethynylestradiol **(3)**, with a relatively unreactive substituent at C-17, was used. As expected, **3** was transformed into two products (Fig. 3). One was a free 6β-hydroxy derivative **15**, and the other one was a methylglucosyl derivative **16**. The ethynyl group at the 17α position makes oxidation of the 17α-hydroxyl group impossible, which explains the lack of Baeyer-Villiger oxidation products.

3-Methoxyestrone **(4)** was used to assess whether *O*-demethylation to estrone **(1)**, as in flavone compounds^25^ or only D-ring transformation, similar to estrone **(1)**, occurs. Surprisingly, **4** was not transformed in the *I*. *fumosorosea* KCh J2 culture. Inhibition of any activity toward this substrate was observed. A free hydroxyl group at C-3 is necessary for transformation of 3-methoxyestrone **(4)** by this strain.

**Figure 4:**
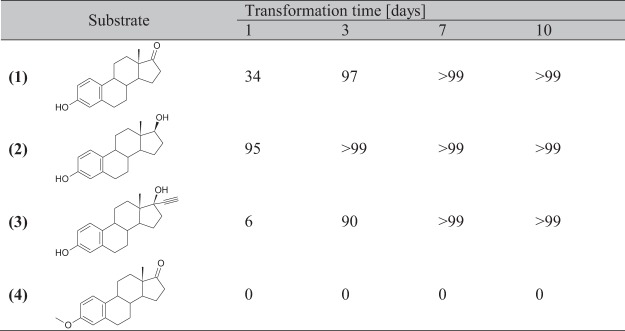
Conversion of the substrate in *Isaria fumosorosea* KCh J2 culture.

The transformation course for all presented substrates was tested using the HPLC technique. Because of the multitude of products from substrates **1**–**3** and their poor separation, it was impossible to determine the percentage composition of the mixture unambiguously. Additionally, NMR with the internal standard was used to establish the transformation pathway for substrates **1**–**3**. In this case, the whole transformation broth was extracted, evaporated and dissolved in a deuterated solvent, but the number of products and their similar spectral data caused the experiment challenging to analyse. However, the amount of unreacted substrate and approximate composition of the products was estimated for all cases (Fig. 4). Taking into account the results of the two methods of tracking the transformation course, it can be said that the transformation of estrone **(1)** in *I*. *fumosorosea* KCh J2 took three days and the main product obtained was **8** which composition in the crude mixture was over 60%. However, after a longer time, a gradual decrease in the amount of this compound is observed due to its conversion into subsequent glycosidic products. Among them, 3-(β-D-4′-*O*-methyloglucosyloxy)-17a-oxa-D-homo-estr-17-one **(9)** was 16% of the crude mixture after 3 days of transformation. The maximum concentration of **9** was observed after ten days, and it reached 25%. The rest of the glycosidic products were in concentration between 1 and 8%. The percentage of products without a glycosidic group did not exceed 10%.

Transformation of estradiol **(2)** was faster, whole added substrate being converted in less than 24 hours, but the obtained products were in nearly equal concentrations. None of the products was in the majority, like in estrone **(1)** transformation. Noteworthy is also definitely a higher percentage of products without a glycosidic group. During the biotransformation estradiol **(2)** their share was recorded at 30%.

In the case of **(3)**, after 24 hours of biotransformation, 6% of the substrate was observed by NMR technique, and the whole conversion occurred in 7 days. During the incubation of this substrate in the culture of the test strain, a constant ratio of both products (determined as 85 to 15% for compounds **16** and **15**, respectively) was observed.

The metabolic pathways of the tested compounds described above have been proposed both based on isolated products and the transformation course analysed by HPLC or NMR. The preparation process of glycoside analogues and their separation needs further development.

Steroid glycosides are known as biotransformation products obtained from the cultures of *Syncephalastrum racemosum* AS 3.264^26^, *Mucor hiemalis*^27^ and many plants^28^. Glucosyloestrogens were obtained in the cultures of *Rhizopus oryzae* AS 3.2380^14^ and microalgae of the genus *Selenastrum*^15^. In the culture of *R*. *oryzae* AS 3.2380 estrogen 3β-glucosides were separated but no further transformation was observed. 96-hour transformation of *Selenastrum capricornutum* produced glucosides of 2-hydroxy and 6β-hydroxyethynylestradiol, but they were in the minority (both 5%). 6β-Hydroxyethynylestradiol was also obtained in the transformation in the culture of *Cephalosporium aphidicola* and *Cunninghamella elegans*^29^ and the alga *Ankistrodesmus braunii*^15^, but no glucosides were detected. To the best of the authors’ knowledge, this is the first description of fungal catalysed methylglycosylation of estrone derivatives and their further transformation.

The reaction of methylglycosylation is usually described for strains of the species *Beauveria bassiana*. The ability of strains from this species to catalyze this reaction for a wide range of substrates (in addition to steroids) has been investigated^30–34^.

It has recently been demonstrated that also other entomopathogenic strains such as *Isaria fumosorosea* and *I*. *farinosa* are capable of attaching a 4-*O*-methylglucose moiety, however, until now, such a transformation has been described only for flavonoid substrates^16,17,25^.

## Conclusions

Biotransformations of steroids using *Isaria fumosorosea* KCh J2 are a valuable source of many derivatives. Estrone derivatives obtained in this study are products of the multienzyme activity of this strain: hydroxylase, reductase, oxidase and glucosyltransferase. All glycosyls obtained in this study are 3-*O*-β-D-(4′-*O*-methyl)-glucopyranosides. Transformations of estradiol, estrone, ethinyloestradiol and methoxyestradiol were performed to evaluate the regioselectivity of glycosylation. Interesting features have been elucidated through this work. First of all, the hydroxyl group at the 6β position most likely is a steric hindrance for glucosylation, because none of the 6β-hydroxylated derivatives was conjugated with a glycosyl moiety. Second, no free 2-hydroxyestrone, as well as conjugated 6β-hydroxy-derivatives, were detected, which suggests that the methylglucosyl moiety is a steric hindrance for 6β-steroid hydroxylase but not for 2-steroid hydroxylase. Third, glycosylated derivatives are more likely to be transformed further, including to lactones. To the best of our knowledge, this is the first demonstration of further transformation of glycosylated estrogens by whole fungal cells.

## Supplementary information

Cascade biotransformation of estrogens by Isaria fumosorosea KCh J2
