# Supplementary material for: Cascade biotransformation of estrogens by Isaria fumosorosea KCh J2
Source: Sci Rep. 2019 Jul 24;9:10734. doi: 10.1038/s41598-019-47225-1 (PMC6656742; doi:10.1038/s41598-019-47225-1)

Supplementary material

Cascade biotransformation of estrogens by *Isaria fumosorosea* KCh J2

Ewa Kozłowska*, Monika Dymarska, Edyta Kostrzewa-Susłow, Tomasz Janeczko*

Department of Chemistry, Wrocław University of Environmental and Life Sciences, Norwida 25, 50-375 Wrocław, Poland

Contents:

Fig.S1. ^{1}H NMR spectral of 6β-hydroxyestrone (**5**) (DMSO-d_6, 600 MHz)
Fig.S2. ^{13}C NMR spectral of 6β-hydroxyestrone (**5**) (DMSO-d_6, 151 MHz)
Fig.S3. HSQC spectral of 6β-hydroxyestrone (**5**) (DMSO-d_6, 151 MHz)
Fig.S4. COSY spectral of 6β-hydroxyestrone (**5**) (DMSO-d_6, 151 MHz)
Fig.S5. ^{1}H NMR spectral of estra-3,6β,17β-triol (**6**) (DMSO-d_6, 600 MHz)
Fig.S6. ^{13}C NMR spectral of estra-3,6β,17β-triol (**6**) (DMSO-d_6, 151 MHz)
Fig.S7. HSQC spectral of estra-3,6β,17β-triol (**6**) (DMSO-d_6, 151 MHz)
Fig.S8. COSY spectral of estra-3,6β,17β-triol (**6**) (DMSO-d_6, 151 MHz)
Fig.S9. ^{1}H NMR spectral of 3,6β-dihydroxy-17a-oxa-D-homo-estrone (**7**) (DMSO-d_6, 600 MHz)
Fig.S10. ^{13}C NMR spectral of 3,6β-dihydroxy-17a-oxa-D-homo-estrone (**7**) (DMSO-d_6, 151 MHz)
Fig.S11. HSQC spectral of 3,6β-dihydroxy-17a-oxa-D-homo-estrone (**7**) (DMSO-d_6, 151 MHz)
Fig.S12. COSY spectral of 3,6β-dihydroxy-17a-oxa-D-homo-estrone (**7**) (DMSO-d_6, 151 MHz)
Fig.S13. ^{1}H NMR spectral of 3-(β-D-4’-O-methyloglucosyloxy)-estrone (**8**) (DMSO-d_6, 600 MHz)
Fig.S14. ^{13}C NMR spectral of 3-(β-D-4’-O-methyloglucosyloxy)-estrone (**8**) (DMSO-d_6, 151 MHz)
Fig.S15. HSQC spectral of 3-(β-D-4’-O-methyloglucosyloxy)-estrone (**8**) (DMSO-d_6, 151 MHz)
Fig.S16. COSY spectral of 3-(β-D-4’-O-methyloglucosyloxy)-estrone (**8**) (DMSO-d_6, 151 MHz)
Fig.S17. HMBC spectral of 3-(β-D-4’-O-methyloglucosyloxy)-estrone (**8**) (DMSO-d_6, 151 MHz)
Fig.S18. ^{1}H NMR spectral of 3-(β-D-4’-O-methyloglucosyloxy)-estrone (**8**) ($CDCl_3$, 600 MHz)
Fig.S19. ^{13}C NMR spectral of 3-(β-D-4’-O-methyloglucosyloxy)-estrone (**8**) ($CDCl_3$, 151 MHz)
Fig.S20. ^{1}H NMR spectral of 3-(β-D-4’-O-methyloglucosyloxy)-17a-oxa-D-homo-estr-17-one (**9**) and 3-(β-D-4’-O-methyloglucosyloxy)-17a-oxa-D-homo-estr-9-en-17-one (**10**) ($CDCl_3$, 600 MHz)
Fig.S21. Part of the ^{13}C NMR spectral of 3-(β-D-4’-O-methyloglucosyloxy)-17a-oxa-D-homo-estr-17-one (**9**) and 3-(β-D-4’-O-methyloglucosyloxy)-17a-oxa-D-homo-estr-9-en-17-one (**10**) ($CDCl_3$, 151 MHz)
Fig.S22. Part of the ^{13}C NMR spectral of 3-(β-D-4’-O-methyloglucosyloxy)-17a-oxa-D-homo-estr-17-one (**9**) and 3-(β-D-4’-O-methyloglucosyloxy)-17a-oxa-D-homo-estr-9-en-17-one (**10**) ($CDCl_3$, 151 MHz)
Fig.S23. HSQC spectral of 3-(β-D-4’-O-methyloglucosyloxy)-17a-oxa-D-homo-estr-17-one (**9**) and 3-(β-D-4’-O-methyloglucosyloxy)-17a-oxa-D-homo-estr-9-en-17-one (**10**) ($CDCl_3$, 151 MHz)
Fig.S24. COSY spectral of 3-(β-D-4’-O-methyloglucosyloxy)-17a-oxa-D-homo-estr-17-one (**9**) and 3-(β-D-4’-O-methyloglucosyloxy)-17a-oxa-D-homo-estr-9-en-17-one (**10**) ($CDCl_3$, 151 MHz)
Fig.S25. ^{1}H NMR spectral of 3-(β-D-4’-O-methyloglucosyloxy)-17a-oxa-D-homo-estr-17-

one (**9**), 3-(β-D-4’-O-methyloglucosyloxy)-17a-oxa-D-homo-estr-9-en-17-one (**10**) and 3-(β-D-4’-O-methyloglucosyloxy)-estr-17β-ol (**12**) ($CDCl_3$, 600 MHz)
Fig.S26. Part of the ^{13}C NMR spectral of 3-(β-D-4’-O-methyloglucosyloxy)-17a-oxa-D-homo-estr-17-one (**9**), 3-(β-D-4’-O-methyloglucosyloxy)-17a-oxa-D-homo-estr-9-en-17-one (**10**) and 3-(β-D-4’-O-methyloglucosyloxy)-estr-17β-ol (**12**) ($CDCl_3$, 151 MHz)
Fig.S27. Part of the ^{13}C NMR spectral of 3-(β-D-4’-O-methyloglucosyloxy)-17a-oxa-D-homo-estr-17-one (**9**), 3-(β-D-4’-O-methyloglucosyloxy)-17a-oxa-D-homo-estr-9-en-17-one (**10**) and 3-(β-D-4’-O-methyloglucosyloxy)-estr-17β-ol (**12**) ($CDCl_3$, 151 MHz)
Fig.S28. Part of the ^{13}C NMR spectral of 3-(β-D-4’-O-methyloglucosyloxy)-17a-oxa-D-homo-estr-17-one (**9**), 3-(β-D-4’-O-methyloglucosyloxy)-17a-oxa-D-homo-estr-9-en-17-one (**10**) and 3-(β-D-4’-O-methyloglucosyloxy)-estr-17β-ol (**12**) ($CDCl_3$, 151 MHz)
Fig.S29. HSQC spectral of 3-(β-D-4’-O-methyloglucosyloxy)-17a-oxa-D-homo-estr-17-one (**9**), 3-(β-D-4’-O-methyloglucosyloxy)-17a-oxa-D-homo-estr-9-en-17-one (**10**) and 3-(β-D-4’-O-methyloglucosyloxy)-estr-17β-ol (**12**) ($CDCl_3$, 151 MHz)
Fig.S30. COSY spectral of 3-(β-D-4’-O-methyloglucosyloxy)-17a-oxa-D-homo-estr-17-one (**9**), 3-(β-D-4’-O-methyloglucosyloxy)-17a-oxa-D-homo-estr-9-en-17-one (**10**) and 3-(β-D-4’-O-methyloglucosyloxy)-estr-17β-ol (**12**) ($CDCl_3$, 151 MHz)
Fig.S31. ^{1}H NMR spectral of 3-(β-D-4’-O-methyloglucosyloxy)-17a-oxa-D-homo-estr-17-one (**9**), 3-(β-D-4’-O-methyloglucosyloxy)-17a-oxa-D-homo-estr-9-en-17-one (**10**) and 3,6β-dihydroxy-17a-oxa-D-homo-estrone (**7**) (DMSO-d_6, 600 MHz)
Fig.S32. Part of the ^{13}C NMR spectral of 3-(β-D-4’-O-methyloglucosyloxy)-17a-oxa-D-homo-estr-17-one (**9**), 3-(β-D-4’-O-methyloglucosyloxy)-17a-oxa-D-homo-estr-9-en-17-one (**10**) and 3,6β-dihydroxy-17a-oxa-D-homo-estrone (**7**) (DMSO-d_6, 151 MHz)
Fig.S33. Part of the ^{13}C NMR spectral of 3-(β-D-4’-O-methyloglucosyloxy)-17a-oxa-D-homo-estr-17-one (**9**), 3-(β-D-4’-O-methyloglucosyloxy)-17a-oxa-D-homo-estr-9-en-17-one (**10**) and 3,6β-dihydroxy-17a-oxa-D-homo-estrone (**7**) (DMSO-d_6, 151 MHz)
Fig.S34. Part of the ^{13}C NMR spectral of 3-(β-D-4’-O-methyloglucosyloxy)-17a-oxa-D-homo-estr-17-one (**9**), 3-(β-D-4’-O-methyloglucosyloxy)-17a-oxa-D-homo-estr-9-en-17-one (**10**) and 3,6β-dihydroxy-17a-oxa-D-homo-estrone (**7**) (DMSO-d_6, 151 MHz)
Fig.S35. ^{1}H NMR spectral of 3-O-(β-D-4’-O-methyloglucopyranosyl)-2-hydroxyestrone (**11**) (DMSO-d_6, 600 MHz)
Fig.S36. ^{13}C NMR spectral of 3-O-(β-D-4’-O-methyloglucopyranosyl)-2-hydroxyestrone (**11**) (DMSO-d_6, 151 MHz)
Fig.S37. HSQC spectral of 3-O-(β-D-4’-O-methyloglucopyranosyl)-2-hydroxyestrone (**11**) (DMSO-d_6, 151 MHz)
Fig.S38. COSY spectral of 3-O-(β-D-4’-O-methyloglucopyranosyl)-2-hydroxyestrone (**11**) (DMSO-d_6, 151 MHz)
Fig.S39. ^{1}H NMR spectral of 3-(β-D-4’-O-methyloglucosyloxy)-estr-17β-ol (**12**) and 3-(β-D-4’-O-methyloglucosyloxy)-estr-9-en-17β-ol (**13**) (DMSO-d_6, 600 MHz)
Fig.S40. Part of the ^{13}C NMR spectral of 3-(β-D-4’-O-methyloglucosyloxy)-estr-17β-ol (**12**) and 3-(β-D-4’-O-methyloglucosyloxy)-estr-9-en-17β-ol (**13**) (DMSO-d_6, 151 MHz)
Fig.S41. Part of the ^{13}C NMR spectral of 3-(β-D-4’-O-methyloglucosyloxy)-estr-17β-ol (**12**) and 3-(β-D-4’-O-methyloglucosyloxy)-estr-9-en-17β-ol (**13**) (DMSO-d_6, 151 MHz)
Fig.S42. HSQC spectral of 3-(β-D-4’-O-methyloglucosyloxy)-estr-17β-ol (**12**) and 3-(β-D-4’-O-methyloglucosyloxy)-estr-9-en-17β-ol (**13**) (DMSO-d_6, 151 MHz)
Fig.S43. COSY spectral of 3-(β-D-4’-O-methyloglucosyloxy)-estr-17β-ol (**12**) and 3-(β-D-4’-O-methyloglucosyloxy)-estr-9-en-17β-ol (**13**) (DMSO-d_6, 151 MHz)
Fig.S44. ^{1}H NMR spectral of estra-3,6β,17β-triol (**6**) and 3-(β-D-4’-O-methyloglucosyloxy)-estr-17β-ol (**12**) (DMSO-d_6, 600 MHz)

Fig.S45. Part of the ^{13}C NMR spectral estra-3,6β,17β-triol (**6**) and 3-(β-D-4'-O-methyloglucosyloxy)-estr-17β-ol (**12**) (DMSO-d_6, 600 MHz)
Fig.S46. Part of the ^{13}C NMR spectral of estra-3,6β,17β-triol (**6**) and 3-(β-D-4'-O-methyloglucosyloxy)-estr-17β-ol (**12**) (DMSO-d_6, 600 MHz)
Fig.S47. HSQC spectral of estra-3,6β,17β-triol (**6**) and 3-(β-D-4'-O-methyloglucosyloxy)-estr-17β-ol (**12**) (DMSO-d_6, 600 MHz)
Fig.S48. ^{1}H NMR spectral of 3-(β-D-4'-O-methyloglucosyloxy)-estr-17β-ol (**12**) and 3-(β-D-4'-O-methyloglucosyloxy)-estr-9-en-17β-ol (**13**) (DMSO-d_6, 600 MHz)
Fig.S49. Part of the ^{13}C NMR spectral of 3-(β-D-4'-O-methyloglucosyloxy)-estr-17β-ol (**12**) and 3-(β-D-4'-O-methyloglucosyloxy)-estr-9-en-17β-ol (**13**) (DMSO-d_6, 151 MHz)
Fig.S50. Part of the ^{13}C NMR spectral of 3-(β-D-4'-O-methyloglucosyloxy)-estr-17β-ol (**12**) and 3-(β-D-4'-O-methyloglucosyloxy)-estr-9-en-17β-ol (**13**) (DMSO-d_6, 151 MHz)
Fig.S51. HSQC spectral of 3-(β-D-4'-O-methyloglucosyloxy)-estr-17β-ol (**12**) and 3-(β-D-4'-O-methyloglucosyloxy)-estr-9-en-17β-ol (**13**) (DMSO-d_6, 151 MHz)
Fig.S52. ^{1}H NMR spectral of 3,6β-dihydroxy-17a-oxa-D-homo-estrone (**7**), 3-(β-D-4'-O-methyloglucosyloxy)-estrone (**8**) and 3-(β-D-4'-O-methyloglucosyloxy)-estr-9-en-17-on (**14**) (DMSO-d_6, 600 MHz)
Fig.S53. Part of the ^{13}C NMR spectral of 3,6β-dihydroxy-17a-oxa-D-homo-estrone (**7**), 3-(β-D-4'-O-methyloglucosyloxy)-estrone (**8**) and 3-(β-D-4'-O-methyloglucosyloxy)-estr-9-en-17-on (**14**) (DMSO-d_6, 600 MHz)
Fig.S54. Part of the ^{13}C NMR spectral of 3,6β-dihydroxy-17a-oxa-D-homo-estrone (**7**), 3-(β-D-4'-O-methyloglucosyloxy)-estrone (**8**) and 3-(β-D-4'-O-methyloglucosyloxy)-estr-9-en-17-on (**14**) (DMSO-d_6, 600 MHz)
Fig.S55. Part of the ^{13}C NMR spectral of 3,6β-dihydroxy-17a-oxa-D-homo-estrone (**7**), 3-(β-D-4'-O-methyloglucosyloxy)-estrone (**8**) and 3-(β-D-4'-O-methyloglucosyloxy)-estr-9-en-17-on (**14**) (DMSO-d_6, 600 MHz)
Fig.S56. HSQC spectral of 3,6β-dihydroxy-17a-oxa-D-homo-estrone (**7**), 3-(β-D-4'-O-methyloglucosyloxy)-estrone (**8**) and 3-(β-D-4'-O-methyloglucosyloxy)-estr-9-en-17-on (**14**) (DMSO-d_6, 600 MHz)
Fig.S57. ^{1}H NMR spectral of 17-ethynyloestra-3,6β,17β-triol (**15**) (DMSO-d_6, 600 MHz)
Fig.S58. ^{13}C NMR spectral of 17-ethynyloestra-3,6β,17β-triol (**15**) (DMSO-d_6, 151 MHz)
Fig.S59. HSQC spectral of 17-ethynyloestra-3,6β,17β-triol (**15**) (DMSO-d_6, 151 MHz)
Fig.S60. COSY spectral of 17-ethynyloestra-3,6β,17β-triol (**15**) (DMSO-d_6, 151 MHz)
Fig.S61. ^{1}H NMR spectral of 3-(β-D-4'-O-methyloglucosyloxy)-17-ethynyloestr-17β-ol (**16**) (DMSO-d_6, 600 MHz)
Fig.S62. ^{13}C NMR spectral of 3-(β-D-4'-O-methyloglucosyloxy)-17-ethynyloestr-17β-ol (**16**) (DMSO-d_6, 151 MHz)
Fig.S63. HSQC spectral of 3-(β-D-4'-O-methyloglucosyloxy)-17-ethynyloestr-17β-ol (**16**) (DMSO-d_6, 151 MHz)
Fig.S64. COSY spectral of 3-(β-D-4'-O-methyloglucosyloxy)-17-ethynyloestr-17β-ol (**16**) (DMSO-d_6, 151 MHz)
Fig.S65. HMBC spectral of 3-(β-D-4'-O-methyloglucosyloxy)-17-ethynyloestr-17β-ol (**16**) (DMSO-d_6,, 151 MHz)
Table 1. R_f values of substrates and biotransformation products
FigS67. TLC plates with substrates and biotransformation products (hexane:acetone 2:1 (*v/v*))
FigS66. TLC plates with substrates and biotransformation products (chloroform:methanol 9:1 (*v/v*))

Fig.S1. ^{1}H NMR spectral of 6β-hydroxyestrone (**5**) (DMSO-d_6, 600 MHz)

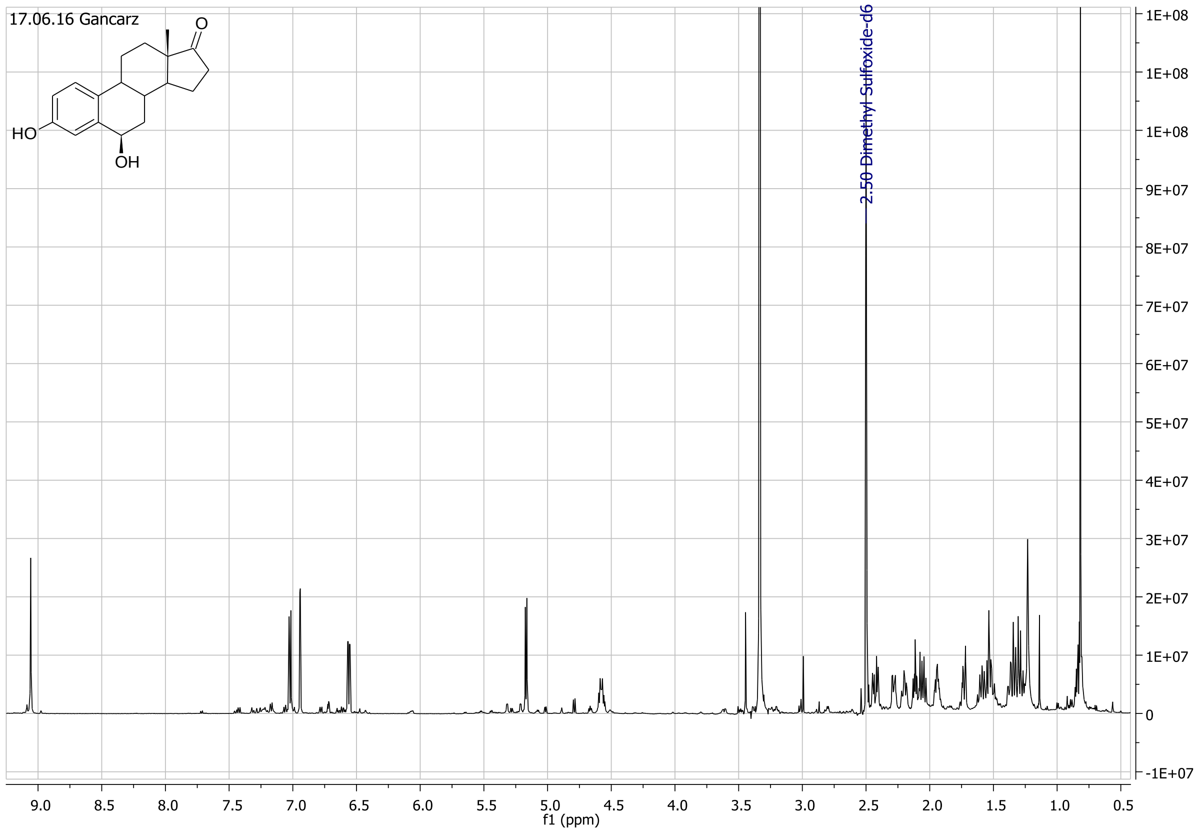

Fig.S2. ^{13}C NMR spectral of 6β-hydroxyestrone (**5**) (DMSO-d_6, 151 MHz)

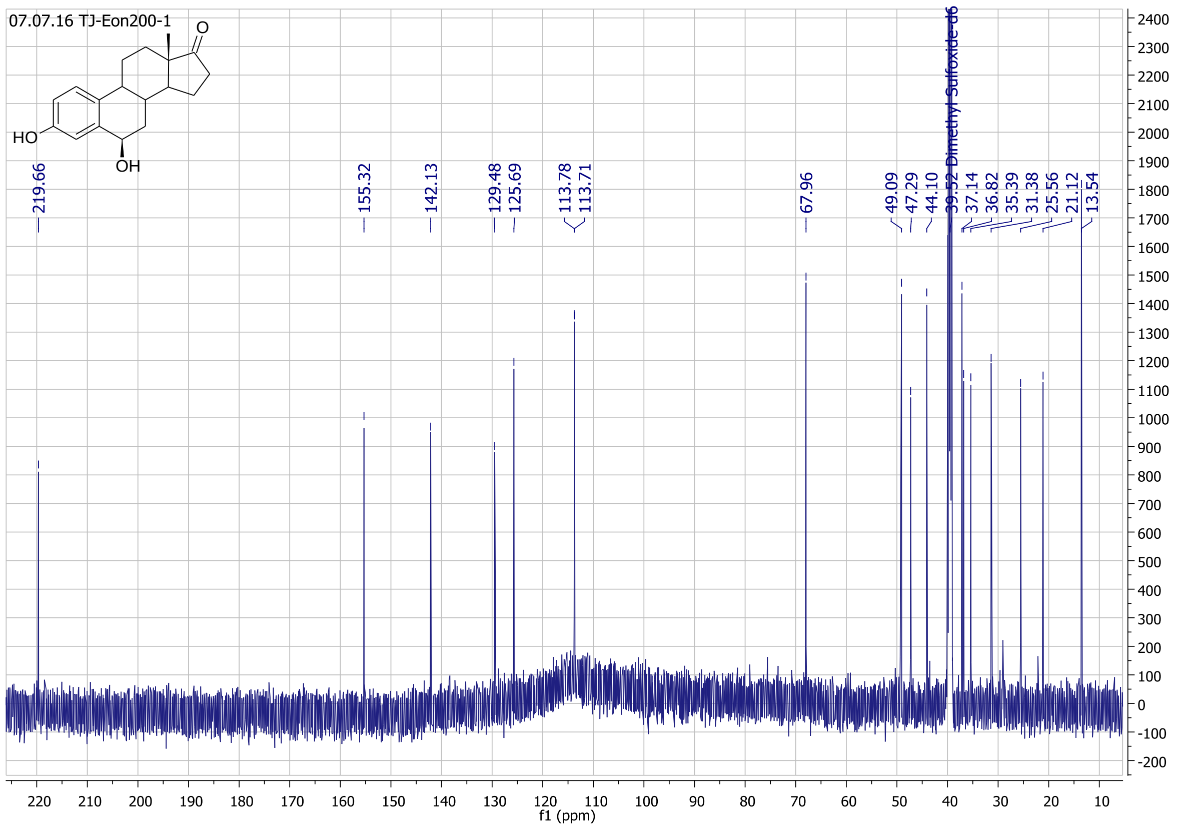

Fig.S3. HSQC spectral of 6β-hydroxyestrone (**5**) (DMSO-d_6, 151 MHz)

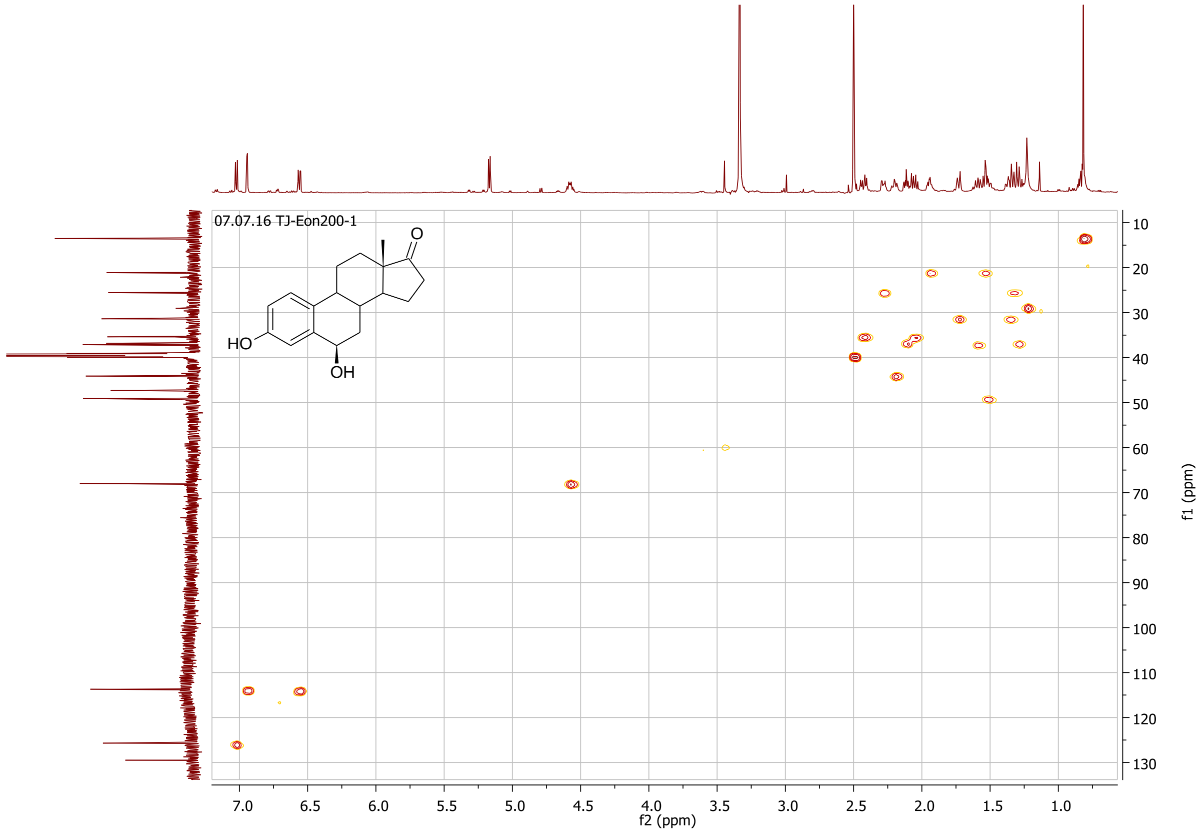

Fig.S4. COSY spectral of 6β-hydroxyestrone (**5**) (DMSO-d_6,, 151 MHz)

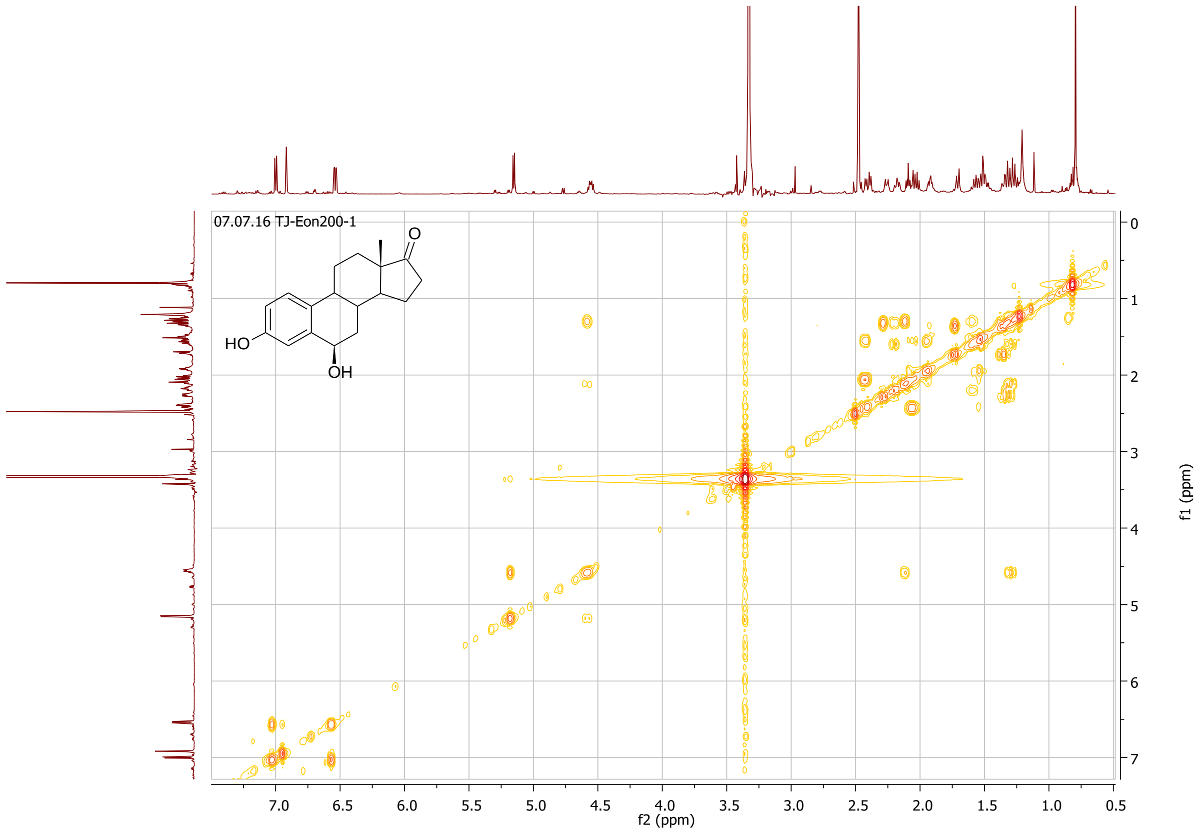

Fig.S5. ^{1}H NMR spectral of estra-3,6β,17β-triol (**6**) (DMSO-d_6, 600 MHz)

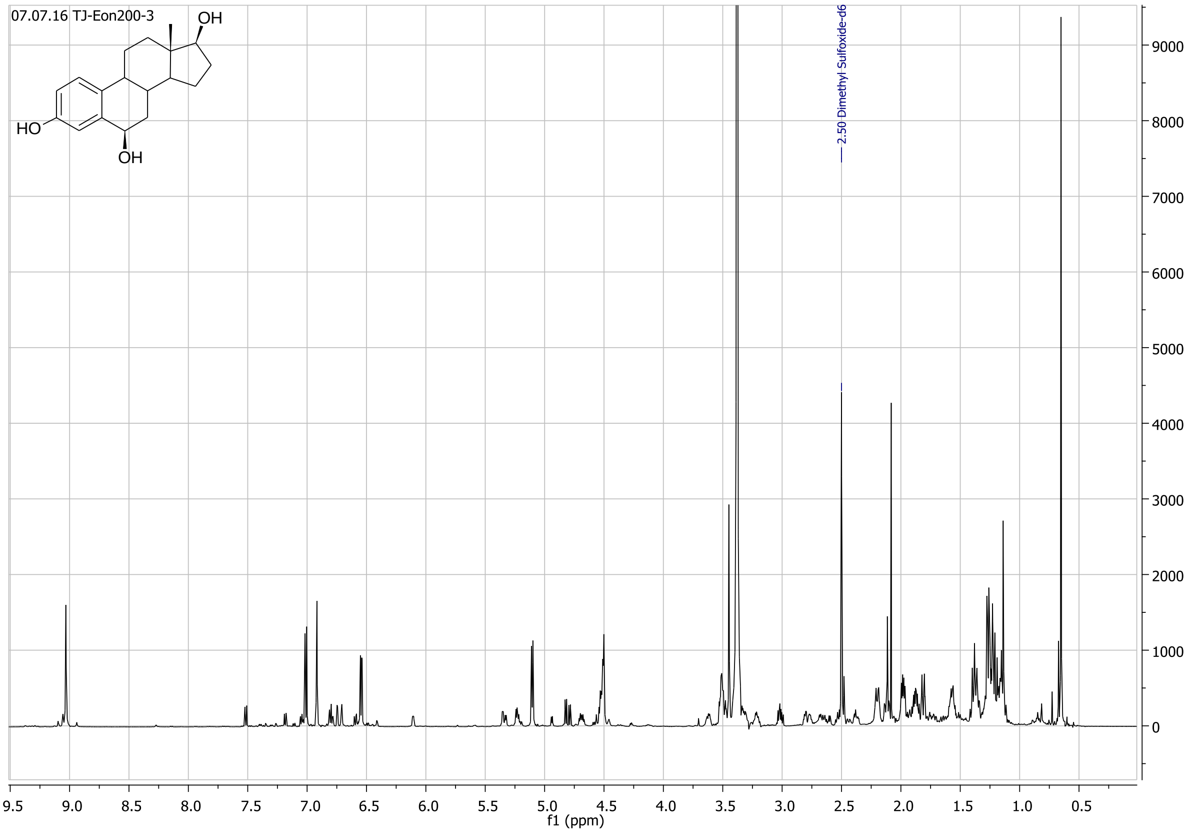

Fig.S6. ^{13}C NMR spectral of estra-3,6β,17β-triol (**6**) (DMSO-d_6, 151 MHz)

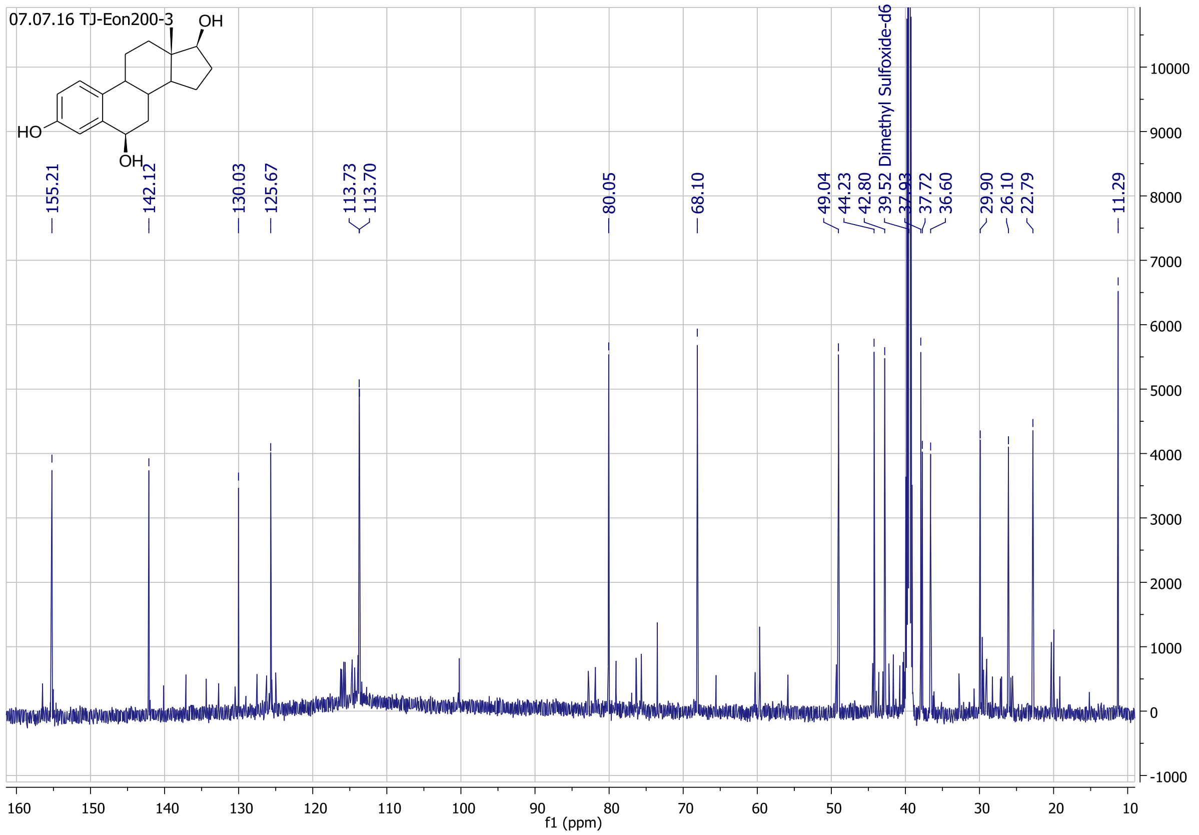

Fig.S7. HSQC spectral of estra-3,6β,17β-triol (**6**) (DMSO-d_6, 151 MHz)

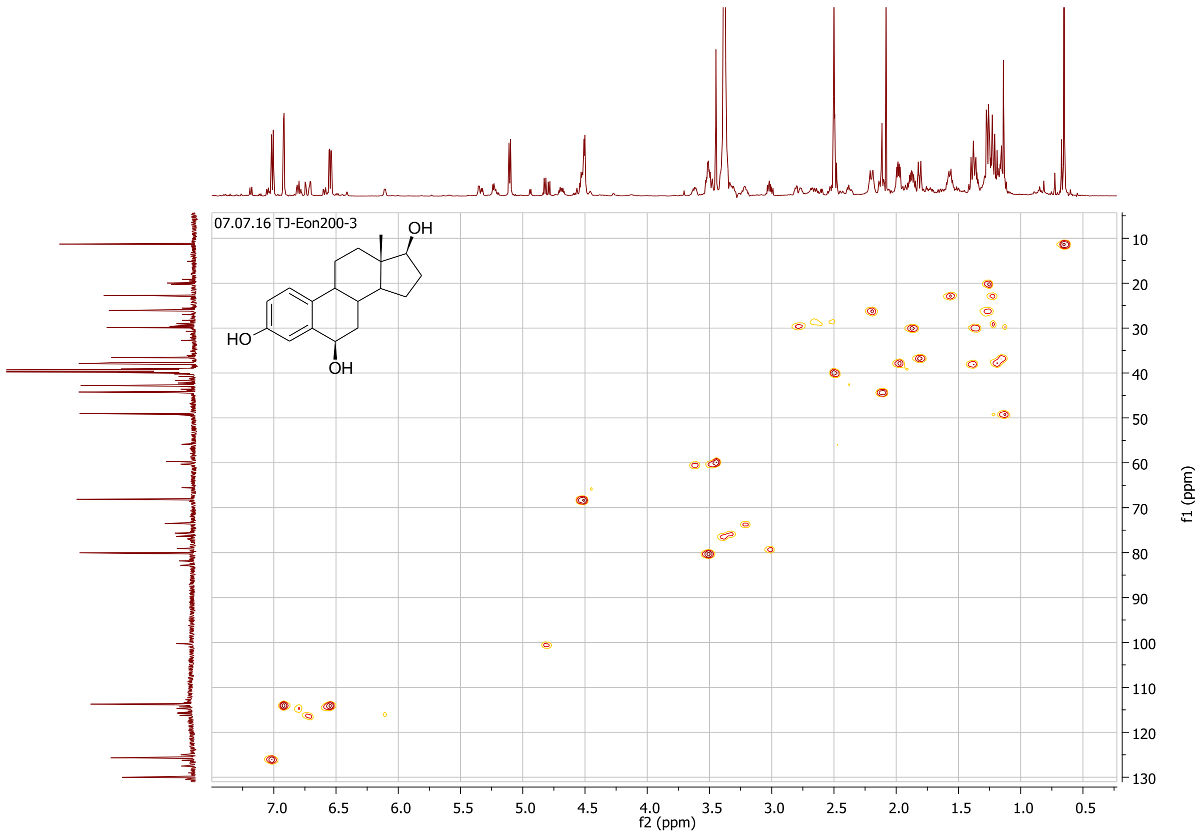

Fig.S8. COSY spectral of estra-3,6β,17β-triol (**6**) (DMSO-d_6,, 151 MHz)

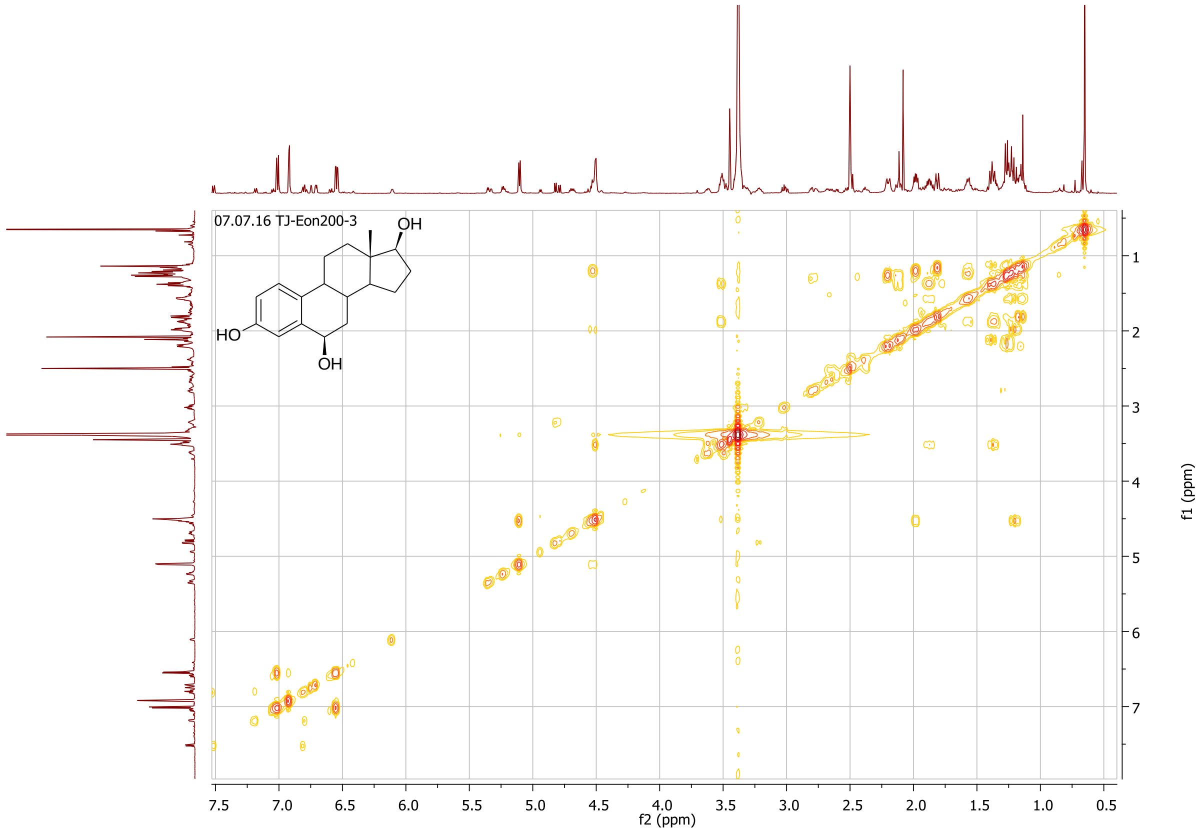

Fig.S9. ^{1}H NMR spectral of 3,6β-dihydroxy-17a-oxa-D-homo-estrone (**7**) (DMSO-d_6, 600 MHz)

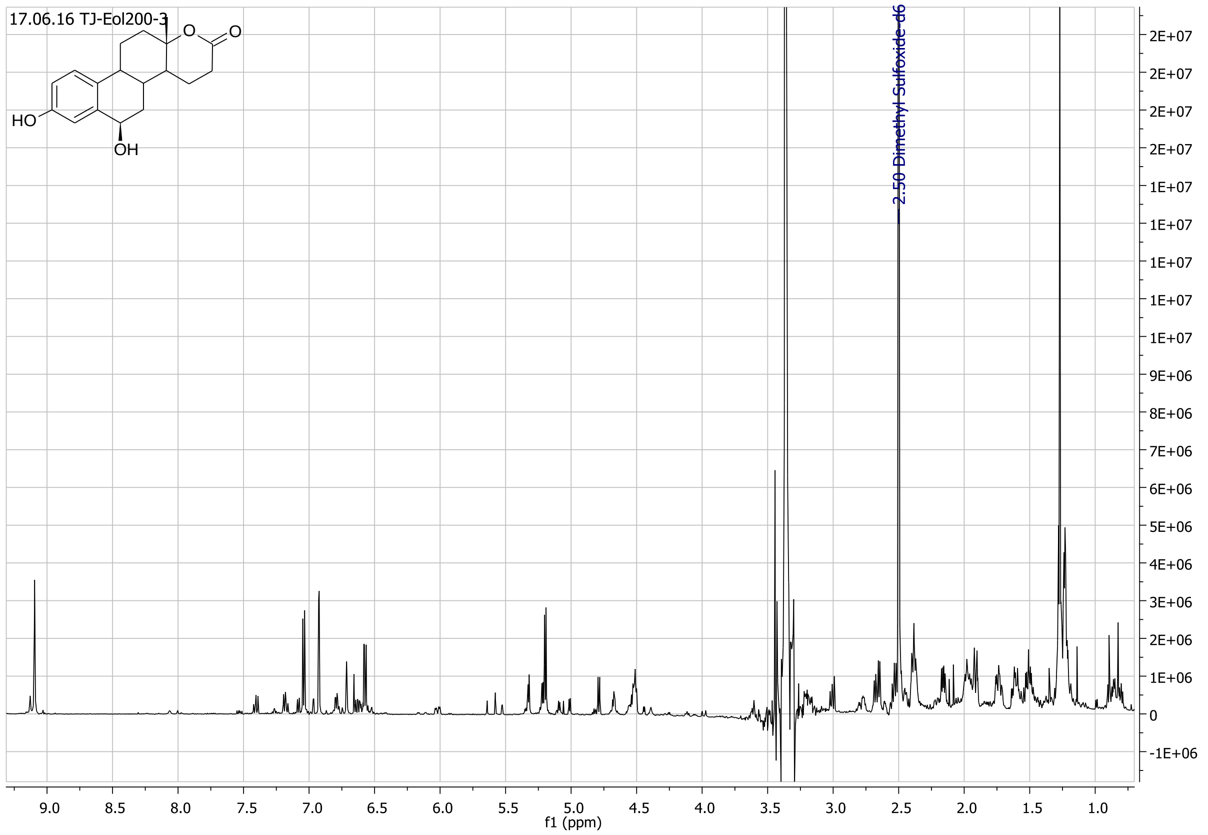

Fig.S10. ^{13}C NMR spectral of 3,6β-dihydroxy-17a-oxa-D-homo-estrone (**7**) (DMSO-d_6, 151 MHz)

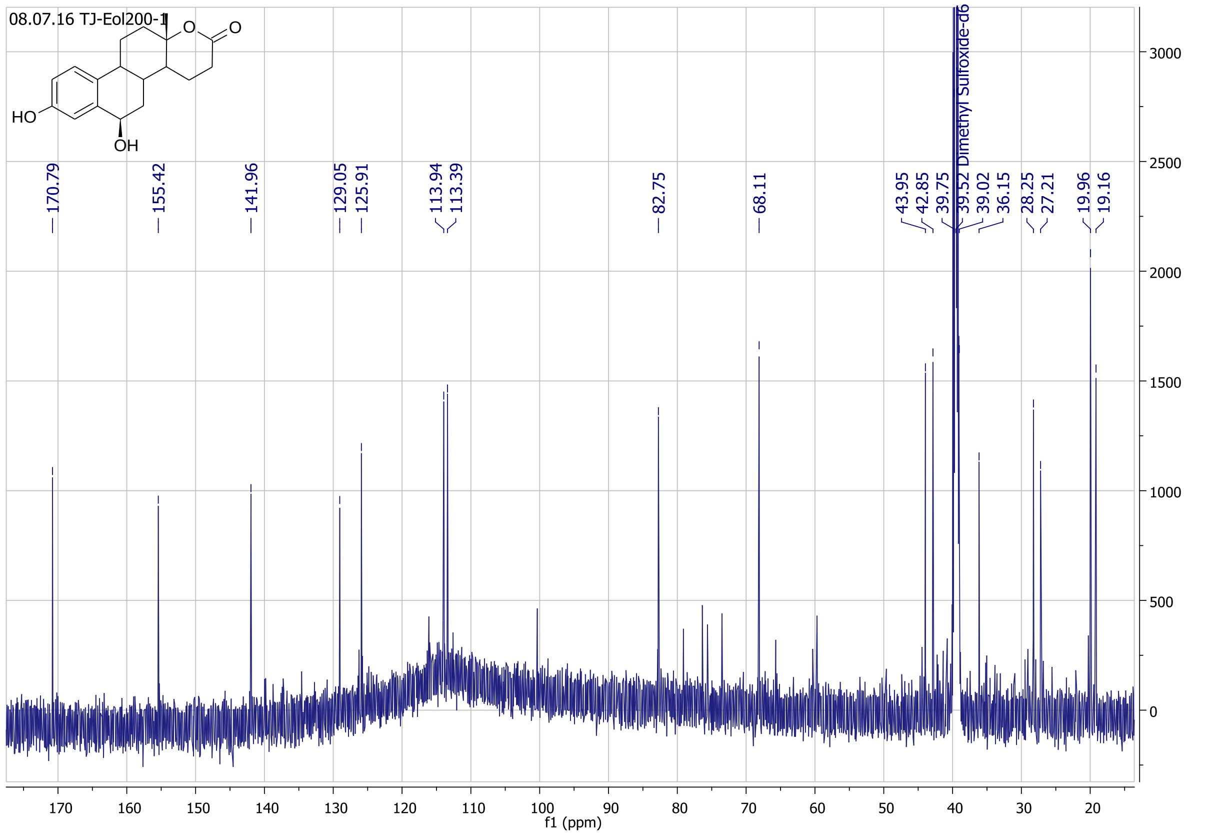

Fig.S11. HSQC spectral of 3,6β-dihydroxy-17a-oxa-D-homo-estrone (**7**) (DMSO-d_6, 151 MHz)

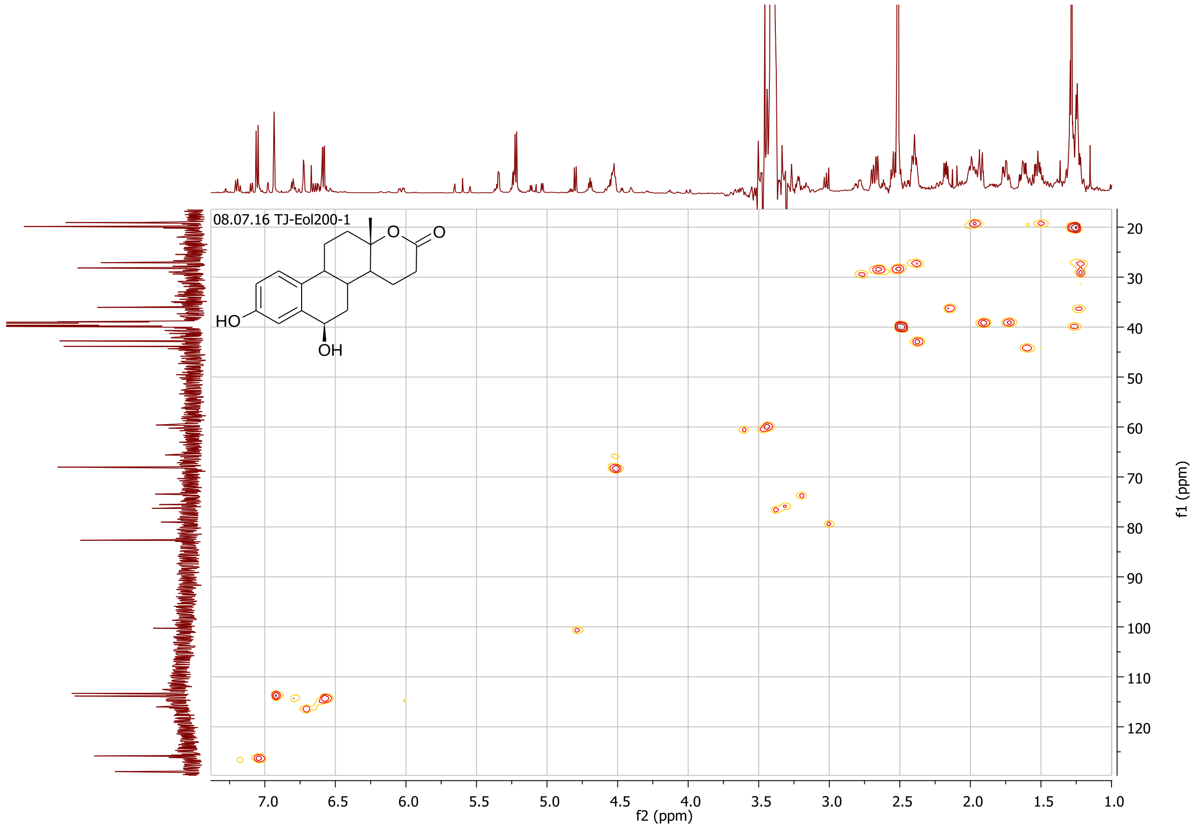

Fig.S12. COSY spectral of 3,6β-dihydroxy-17a-oxa-D-homo-estrone (**7**) (DMSO-d_6,, 151 MHz)

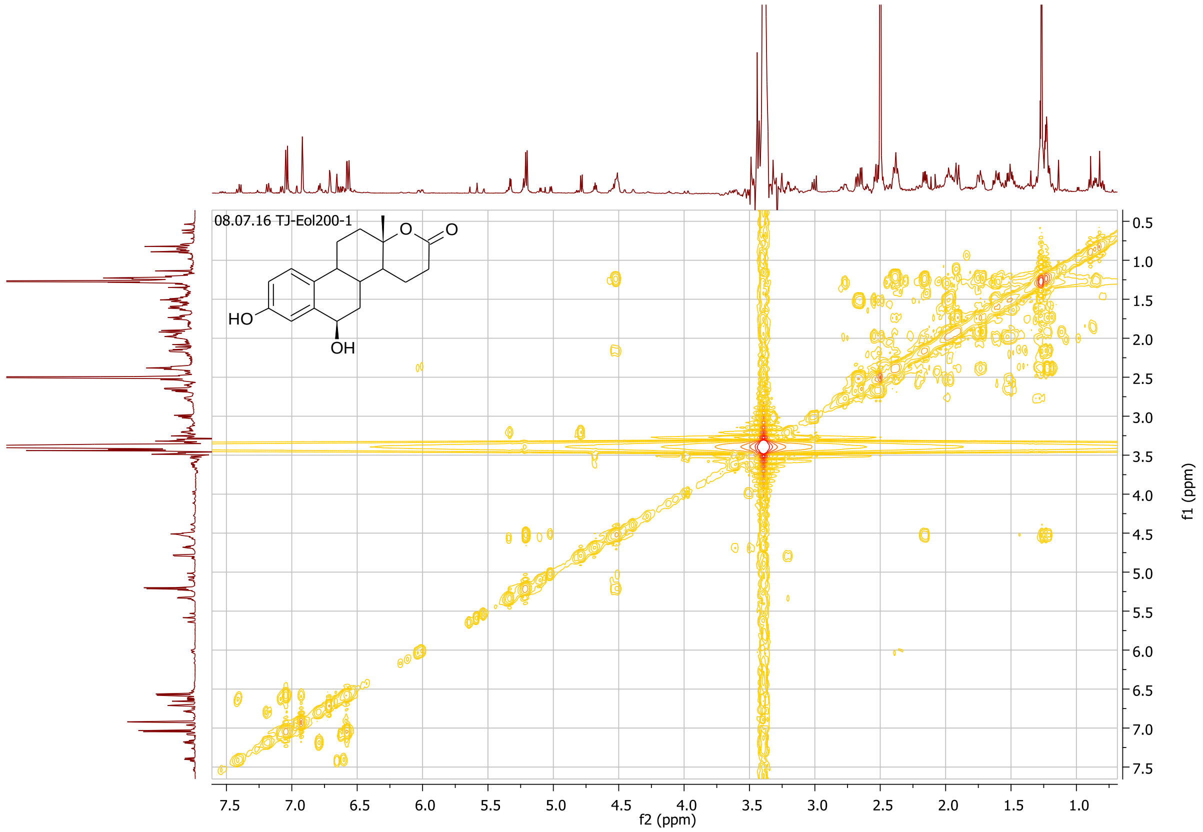

Fig.S13. ^{1}H NMR spectral of 3-(β-D-4'-O-methyloglucosyloxy)-estrone (**8**) (DMSO-d_6, 600 MHz)

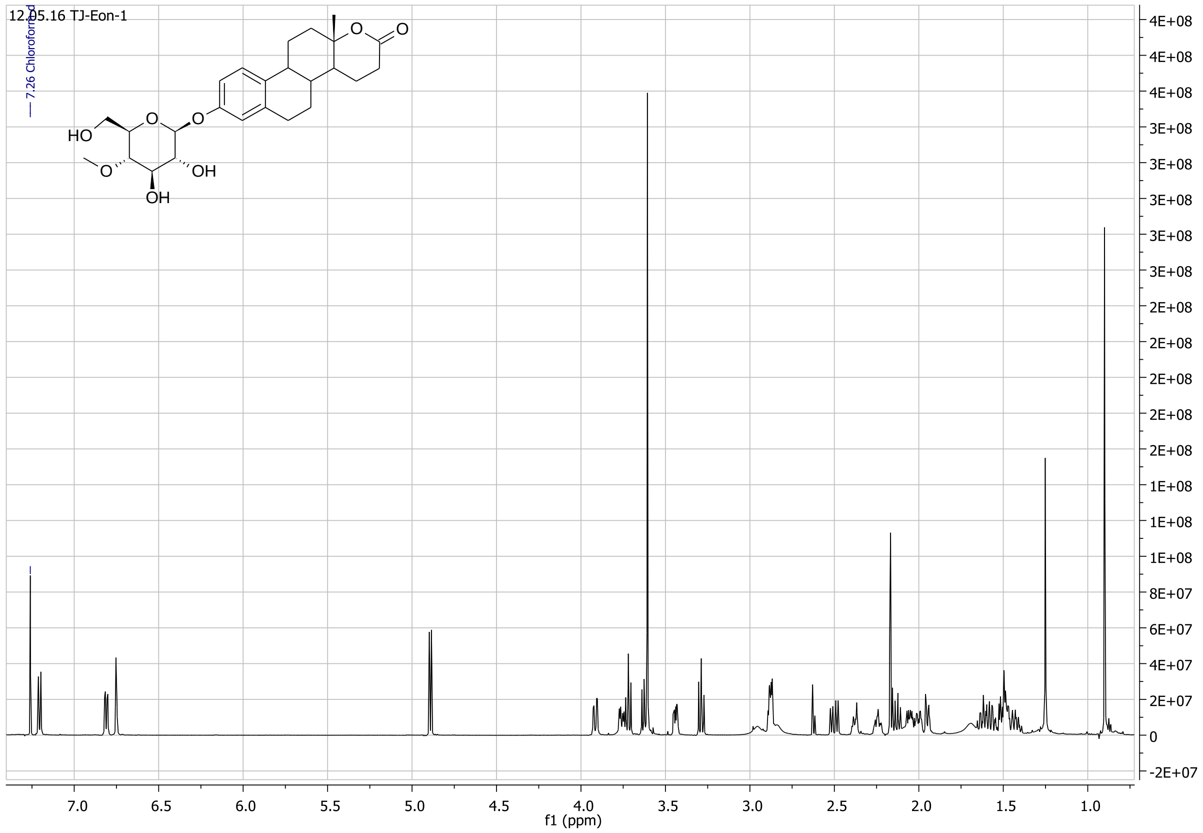

Fig.S14. ^{13}C NMR spectral of 3-(β-D-4'-O-methyloglucosyloxy)-estrone (**8**) (DMSO-d_6, 151 MHz)

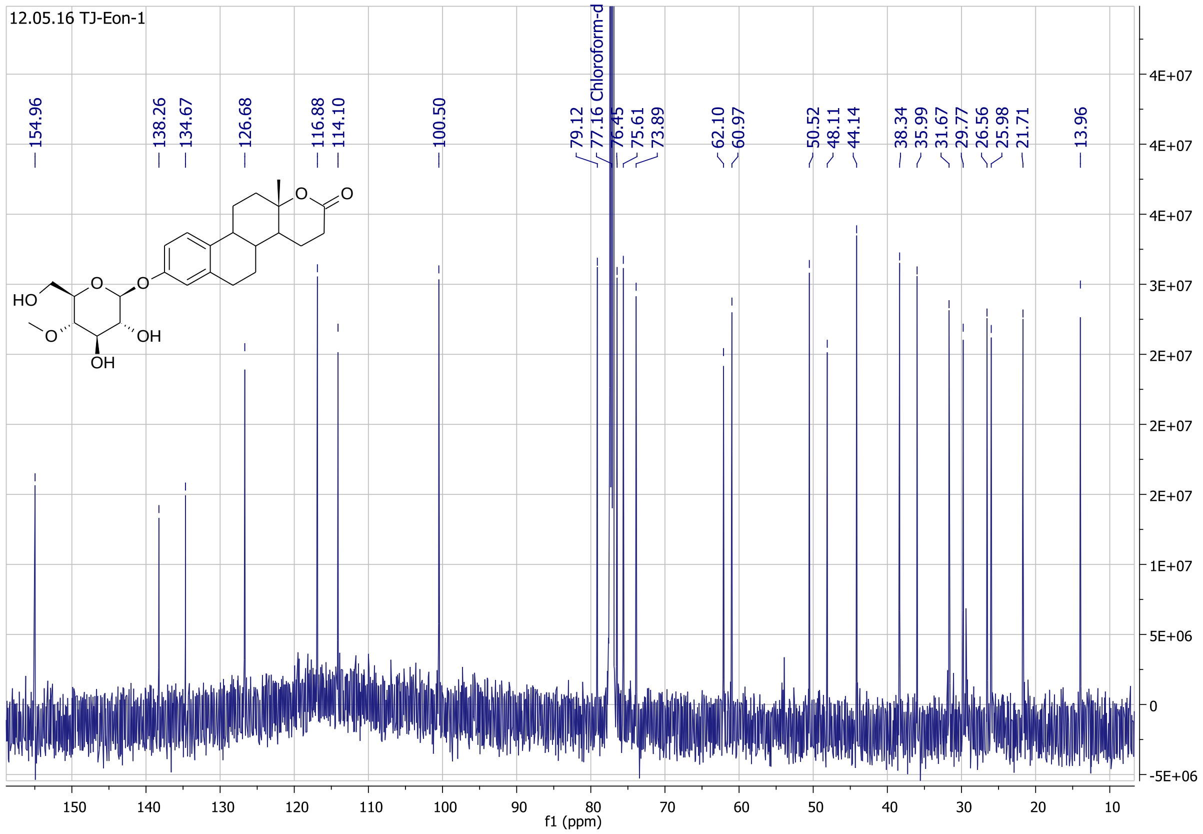

Fig.S15. HSQC spectral of 3-(β-D-4'-O-methyloglucosyloxy)-estrone (**8**) (DMSO-d_6, 151 MHz)

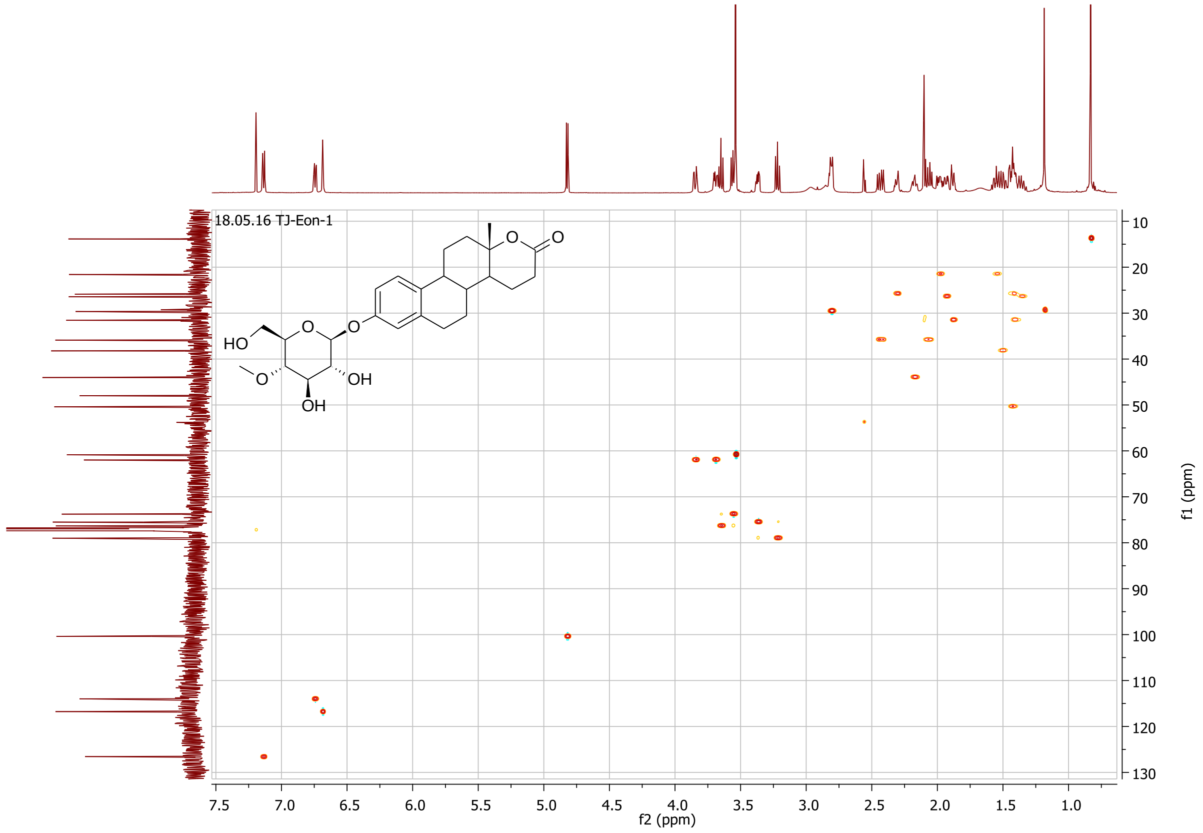

Fig.S16. COSY spectral of 3-(β-D-4'-O-methyloglucosyloxy)-estrone (**8**) (DMSO-d_6,, 151 MHz)

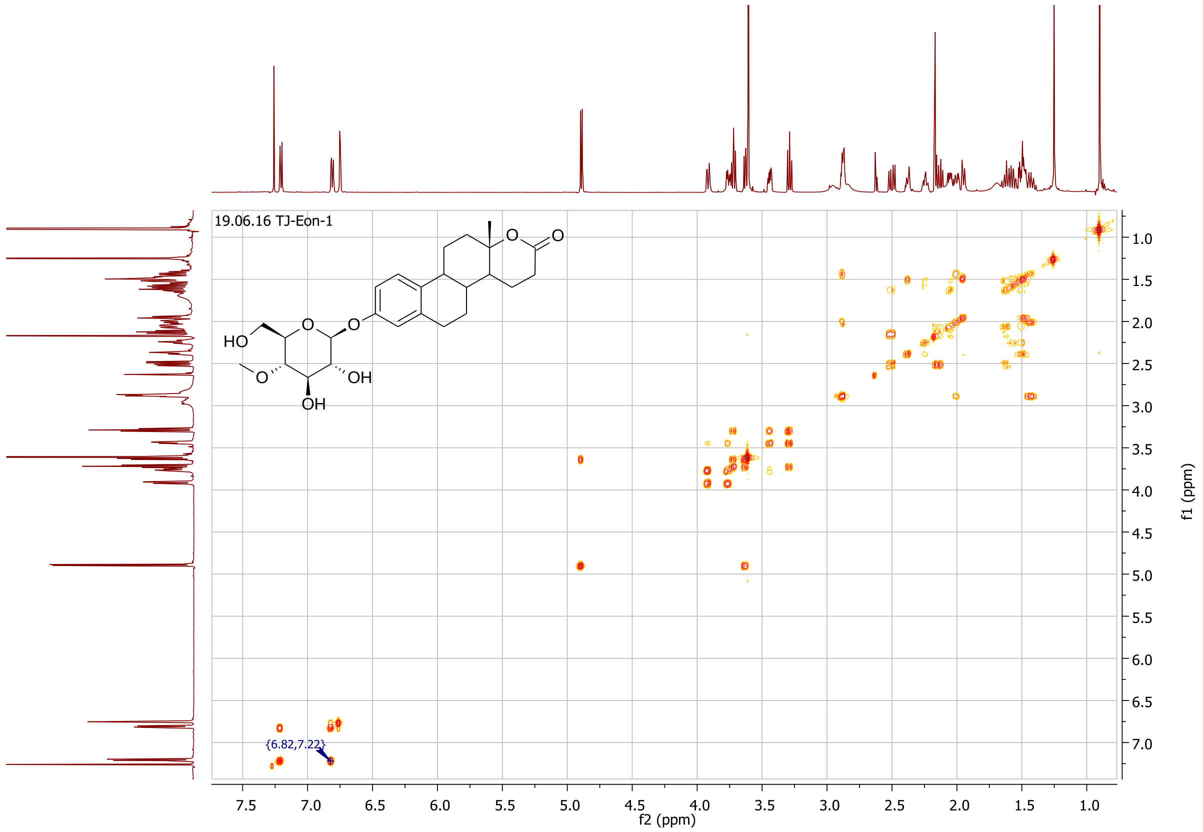

Fig.S17. HMBC spectral of 3-(β-D-4’-O-methyloglucosyloxy)-estrone (**8**) (DMSO-*d*$_6$,, 151 MHz)

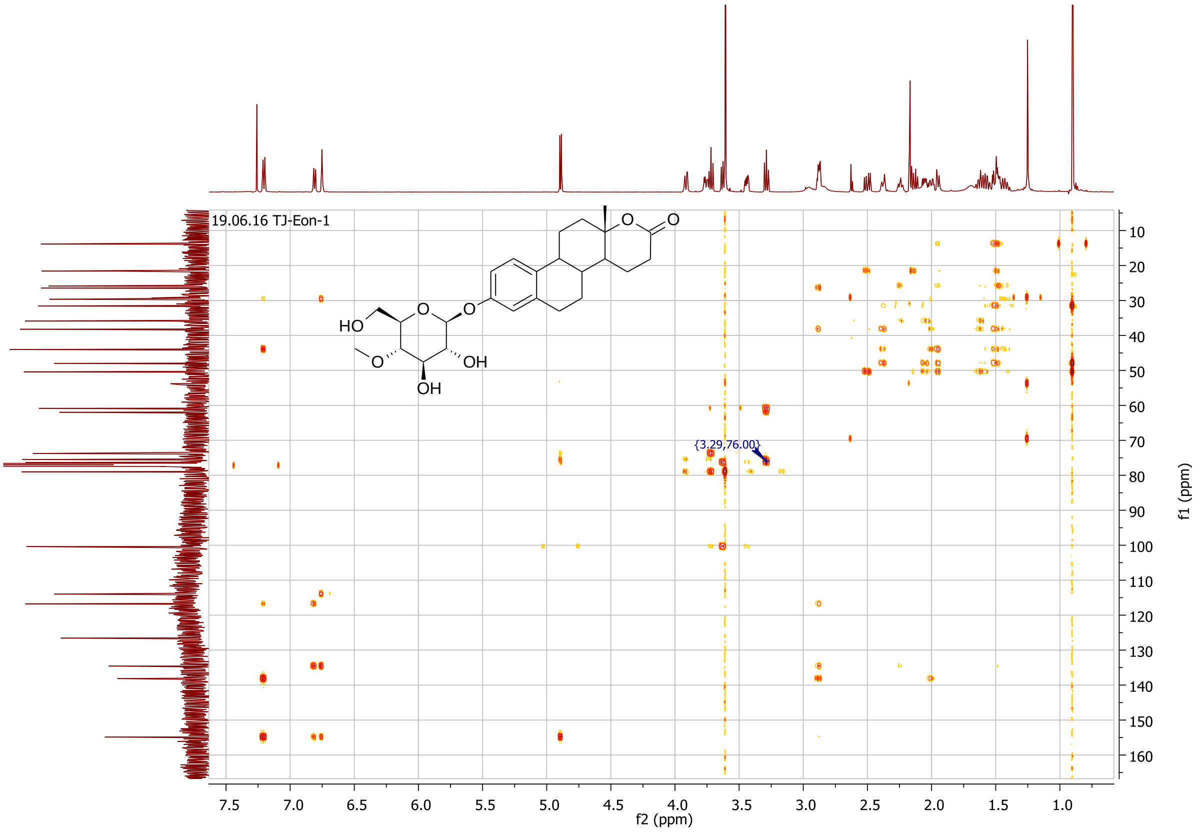

Fig.S18. ^{1}H NMR spectral of 3-(β-D-4’-O-methyloglucosyloxy)-estrone (**8**) (CDCl$_3$, 600 MHz)

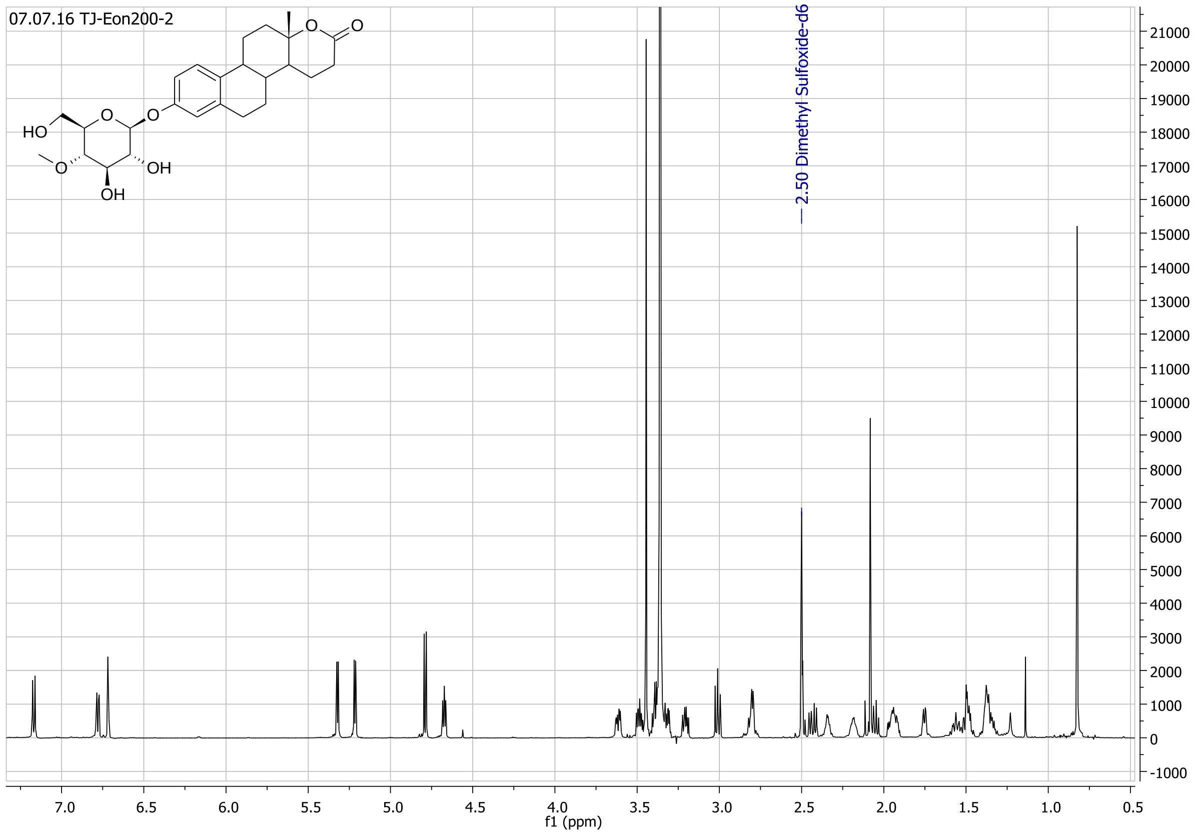

Fig.S19. ^{13}C NMR spectral of 3-(β-D-4'-O-methyloglucosyloxy)-estrone (**8**) ($CDCl_3$, 151 MHz)

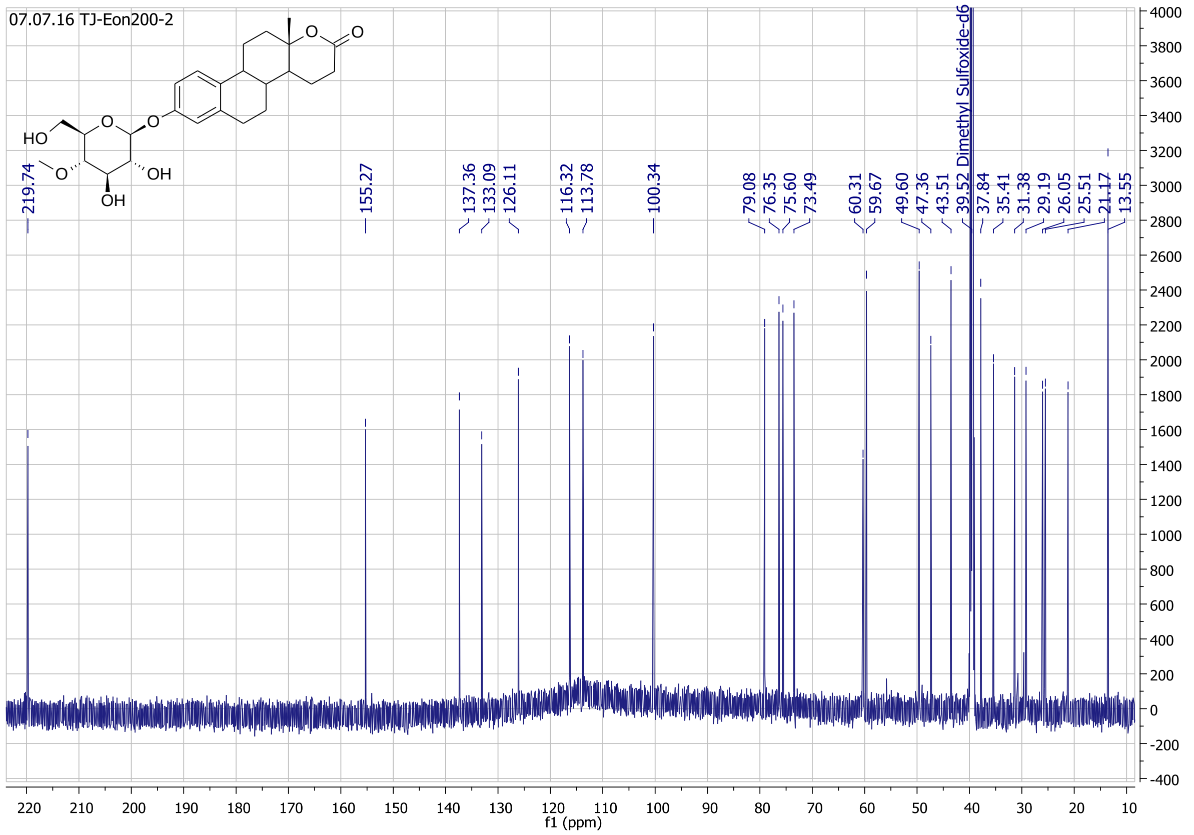

Fig.S20. ^{1}H NMR spectral of 3-(β-D-4'-O-methyloglucosyloxy)-17a-oxa-D-homo-estr-17-one (**9**) and 3-(β-D-4'-O-methyloglucosyloxy)-17a-oxa-D-homo-estr-9-en-17-one (**10**) ($CDCl_3$, 600 MHz)

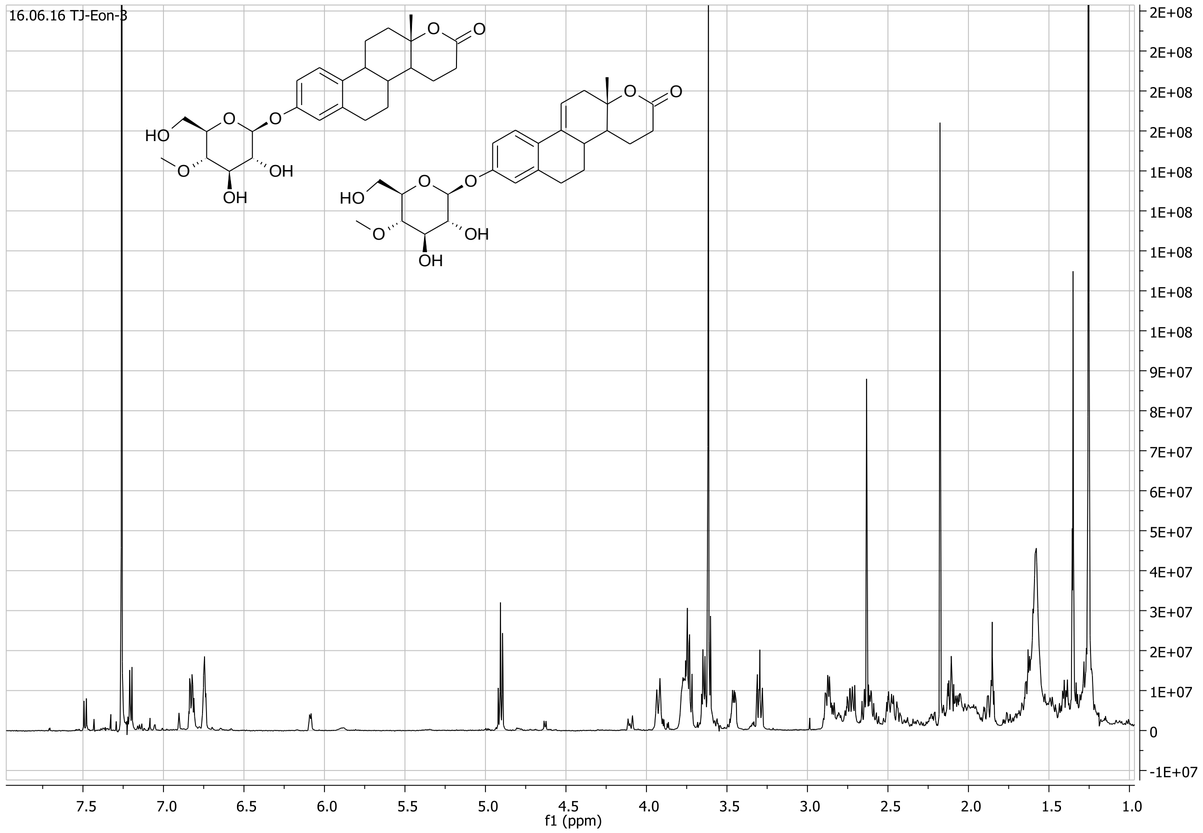

Fig.S21. Part of the ^{13}C NMR spectral of 3-(β-D-4'-O-methyloglucosyloxy)-17a-oxa-D-homo-estr-17-one (**9**) and 3-(β-D-4'-O-methyloglucosyloxy)-17a-oxa-D-homo-estr-9-en-17-one (**10**) ($CDCl_3$, 151 MHz)

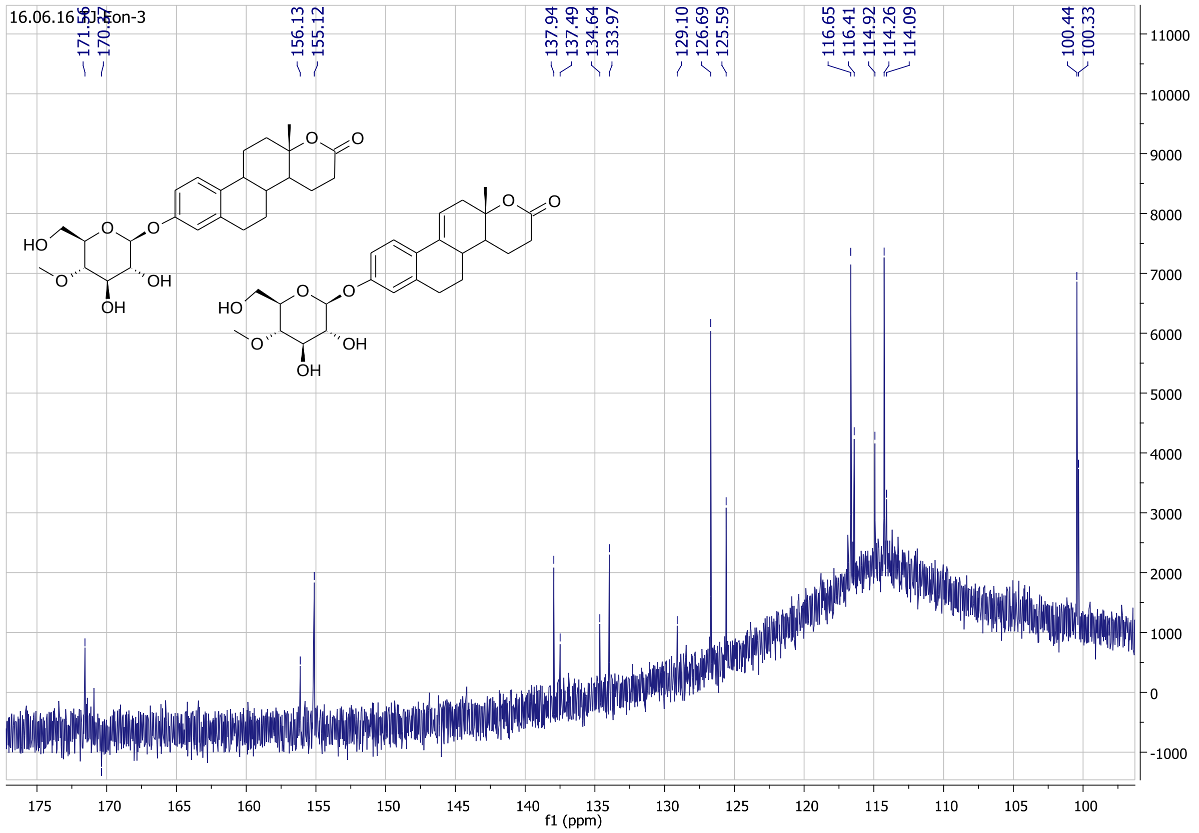

Fig.S22. Part of the ^{13}C NMR spectral of 3-(β-D-4'-O-methyloglucosyloxy)-17a-oxa-D-homo-estr-17-one (**9**) and 3-(β-D-4'-O-methyloglucosyloxy)-17a-oxa-D-homo-estr-9-en-17-one (**10**) ($CDCl_3$, 151 MHz)

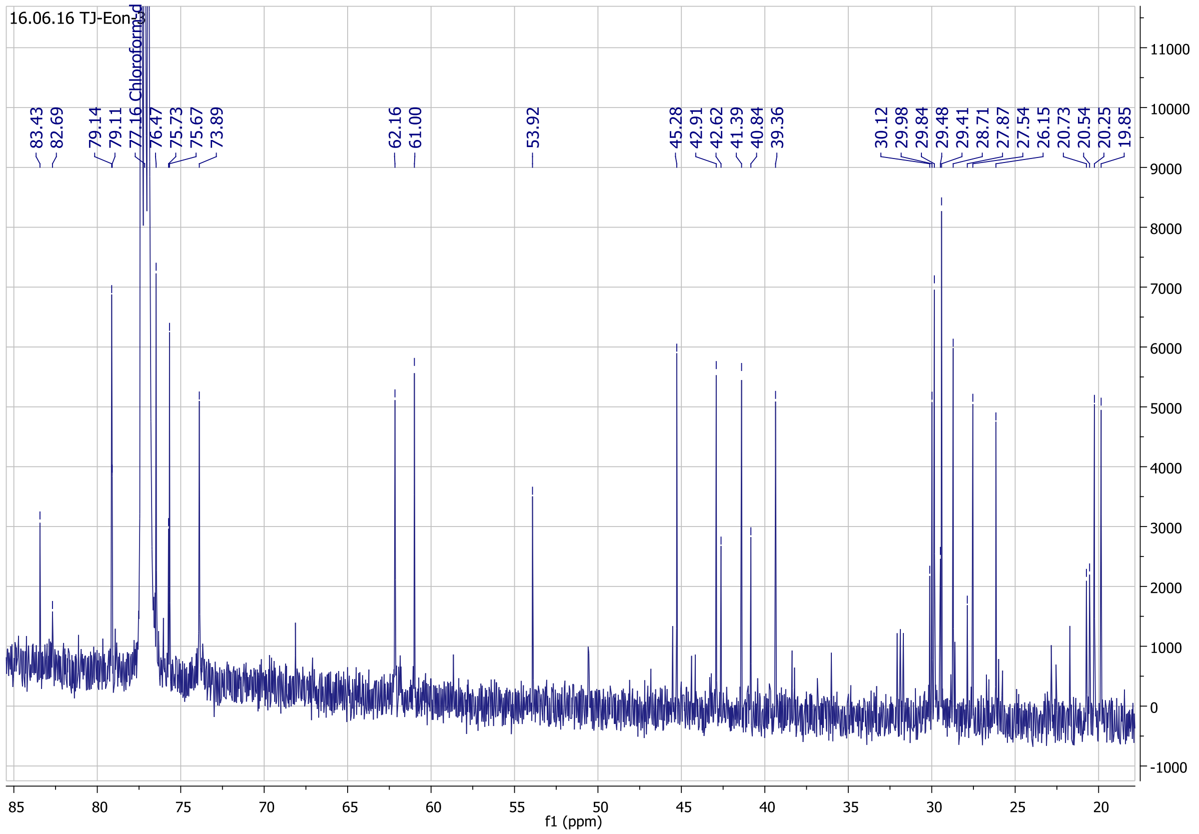

Fig.S23. HSQC spectral of 3-(β-D-4'-O-methyloglucosyloxy)-17a-oxa-D-homo-estr-17-one (**9**) and 3-(β-D-4'-O-methyloglucosyloxy)-17a-oxa-D-homo-estr-9-en-17-one (**10**) ($CDCl_3$, 151 MHz)

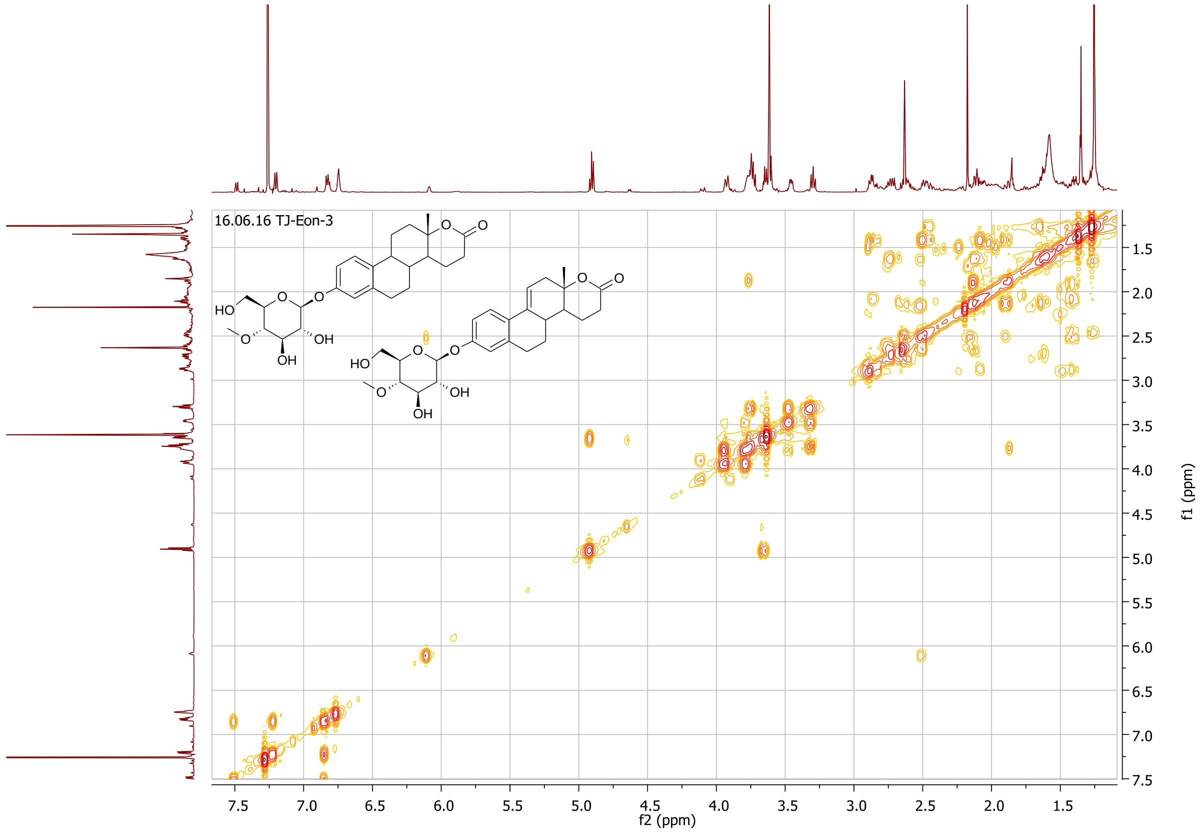

Fig.S24. COSY spectral of 3-(β-D-4'-O-methyloglucosyloxy)-17a-oxa-D-homo-estr-17-one (**9**) and 3-(β-D-4'-O-methyloglucosyloxy)-17a-oxa-D-homo-estr-9-en-17-one (**10**) ($CDCl_3$, 151 MHz)

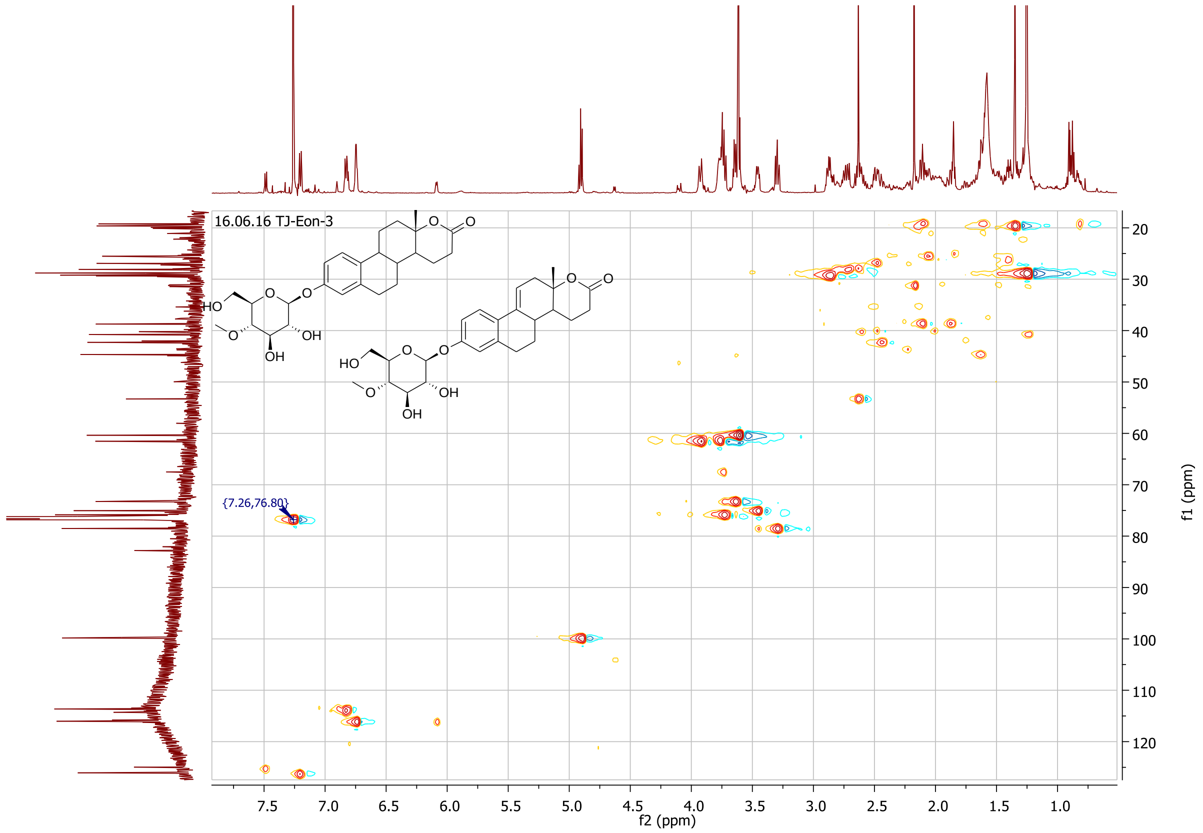

Fig.S25. ^{1}H NMR spectral of 3-(β-D-4'-O-methyloglucosyloxy)-17a-oxa-D-homo-estr-17-one (**9**), 3-(β-D-4'-O-methyloglucosyloxy)-17a-oxa-D-homo-estr-9-en-17-one (**10**) and 3-(β-D-4'-O-methyloglucosyloxy)-estr-17β-ol (**12**) ($CDCl_3$, 600 MHz)

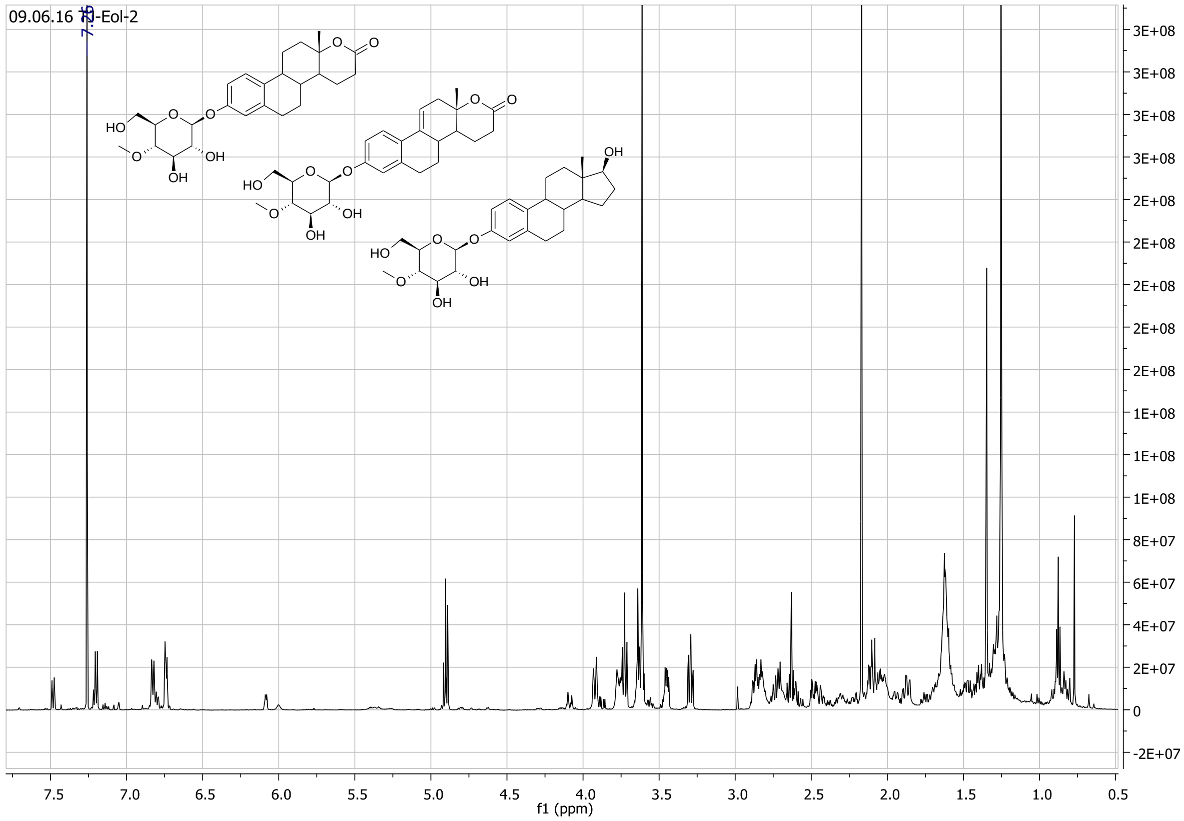

Fig.S26. Part of the ^{13}C NMR spectral of 3-(β-D-4'-O-methyloglucosyloxy)-17a-oxa-D-homo-estr-17-one (**9**), 3-(β-D-4'-O-methyloglucosyloxy)-17a-oxa-D-homo-estr-9-en-17-one (**10**) and 3-(β-D-4'-O-methyloglucosyloxy)-estr-17β-ol (**12**) ($CDCl_3$, 151 MHz)

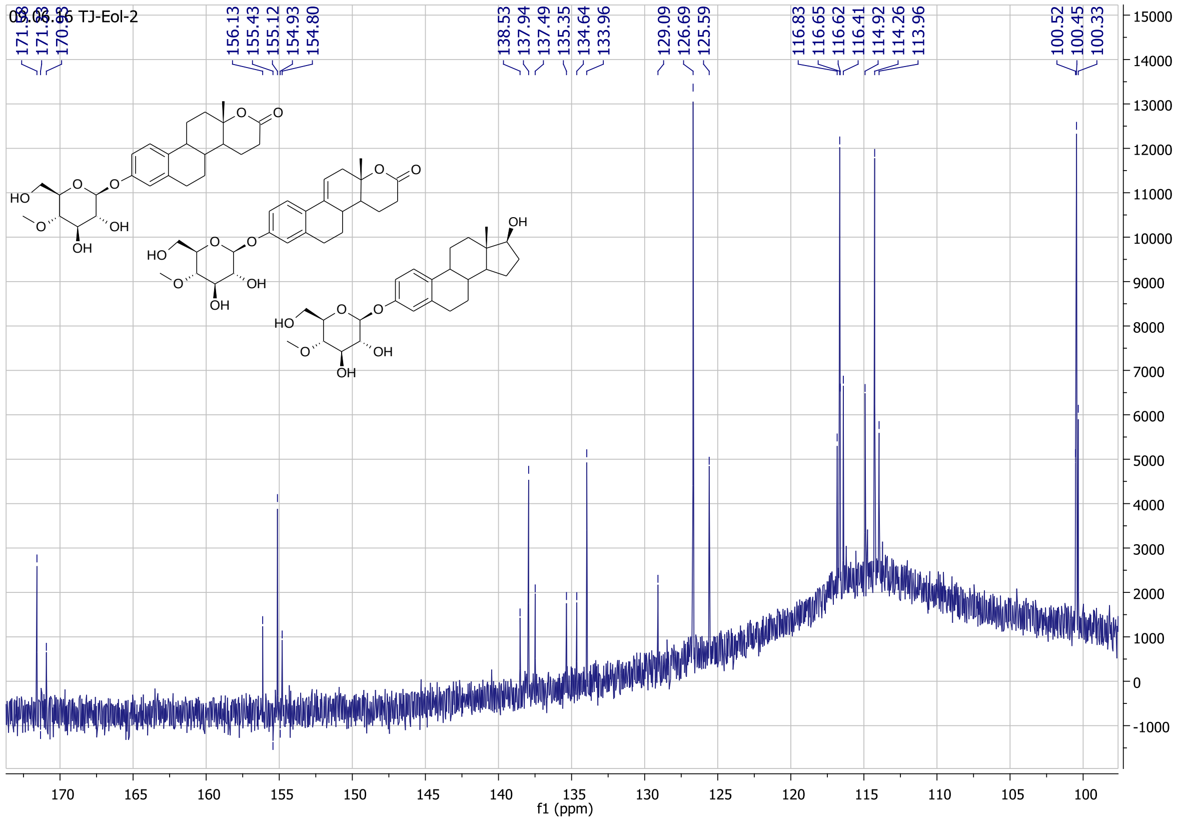

Fig.S27. Part of the ^{13}C NMR spectral of 3-(β-D-4'-O-methyloglucosyloxy)-17a-oxa-D-homo-estr-17-one (**9**), 3-(β-D-4'-O-methyloglucosyloxy)-17a-oxa-D-homo-estr-9-en-17-one (**10**) and 3-(β-D-4'-O-methyloglucosyloxy)-estr-17β-ol (**12**) ($CDCl_3$, 151 MHz)

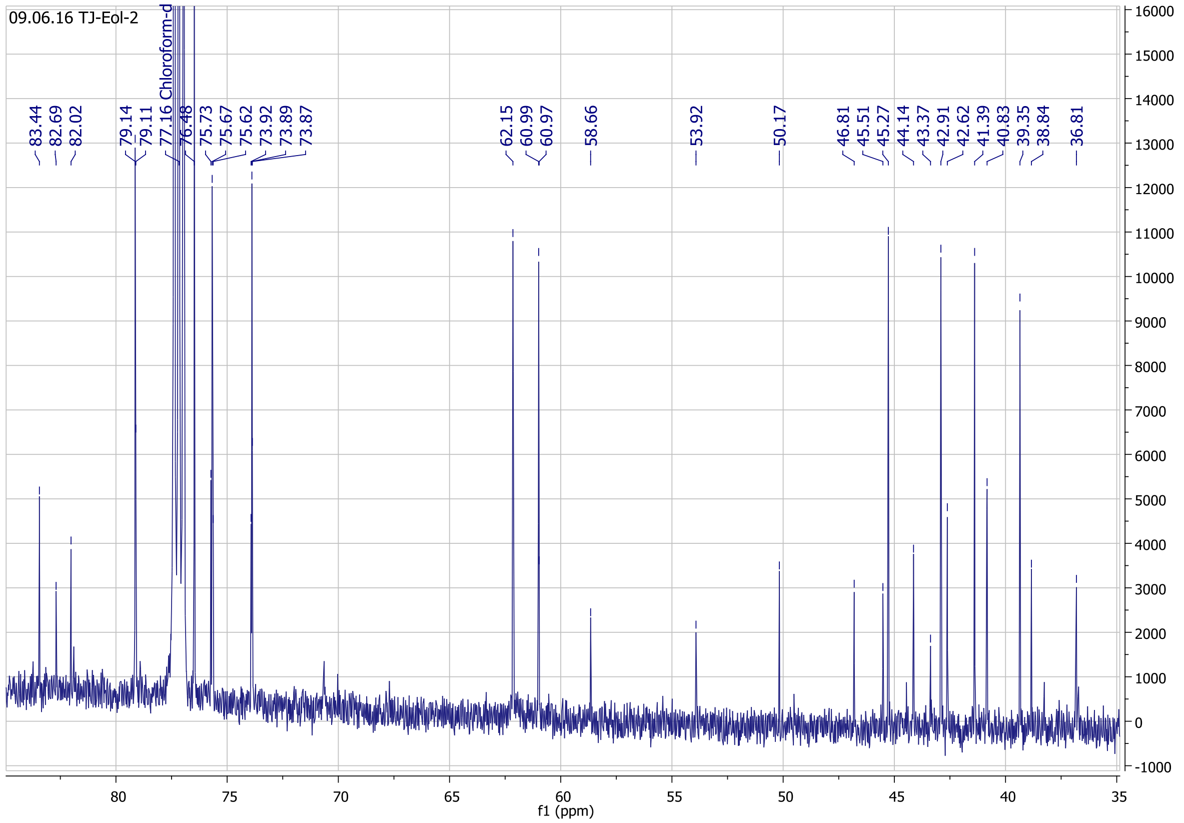

Fig.S28. Part of the ^{13}C NMR spectral of 3-(β-D-4'-O-methyloglucosyloxy)-17a-oxa-D-homo-estr-17-one (**9**), 3-(β-D-4'-O-methyloglucosyloxy)-17a-oxa-D-homo-estr-9-en-17-one (**10**) and 3-(β-D-4'-O-methyloglucosyloxy)-estr-17β-ol (**12**) ($CDCl_3$, 151 MHz)

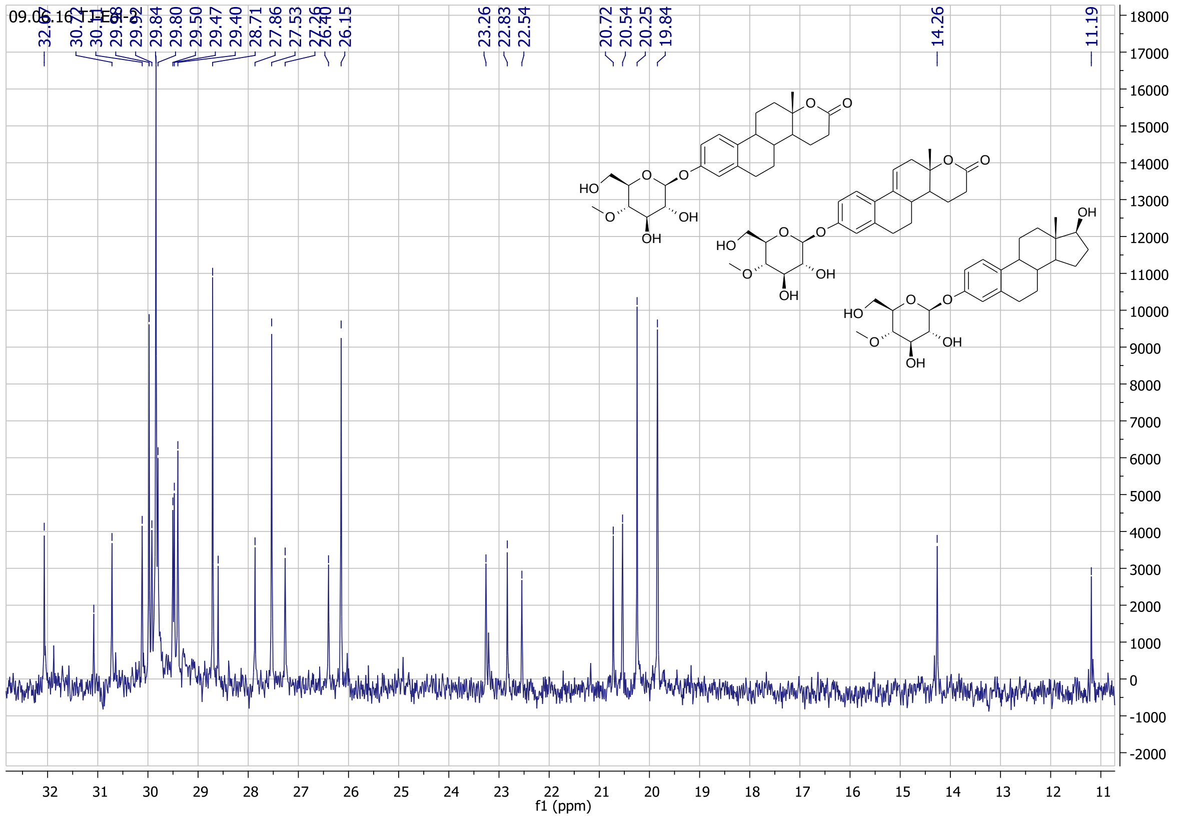

Fig.S29. HSQC spectral of 3-(β-D-4’-O-methylglucosyloxy)-17a-oxa-D-homo-estr-17-one (**9**), 3-(β-D-4’-O-methylglucosyloxy)-17a-oxa-D-homo-estr-9-en-17-one (**10**) and 3-(β-D-4’-O-methylglucosyloxy)-estr-17β-ol (**12**) ($CDCl_3$, 151 MHz)

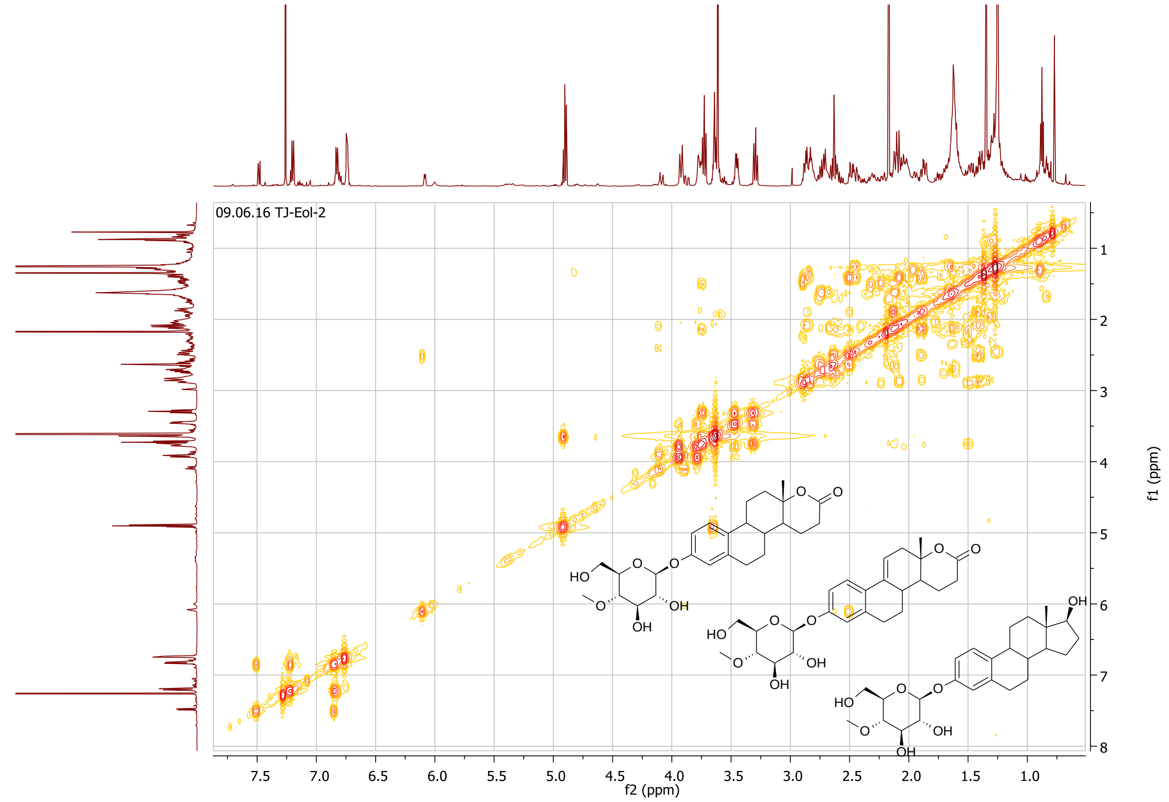

Fig.S30. COSY spectral of 3-(β-D-4’-O-methylglucosyloxy)-17a-oxa-D-homo-estr-17-one (**9**), 3-(β-D-4’-O-methylglucosyloxy)-17a-oxa-D-homo-estr-9-en-17-one (**10**) and 3-(β-D-4’-O-methylglucosyloxy)-estr-17β-ol (**12**) ($CDCl_3$, 151 MHz)

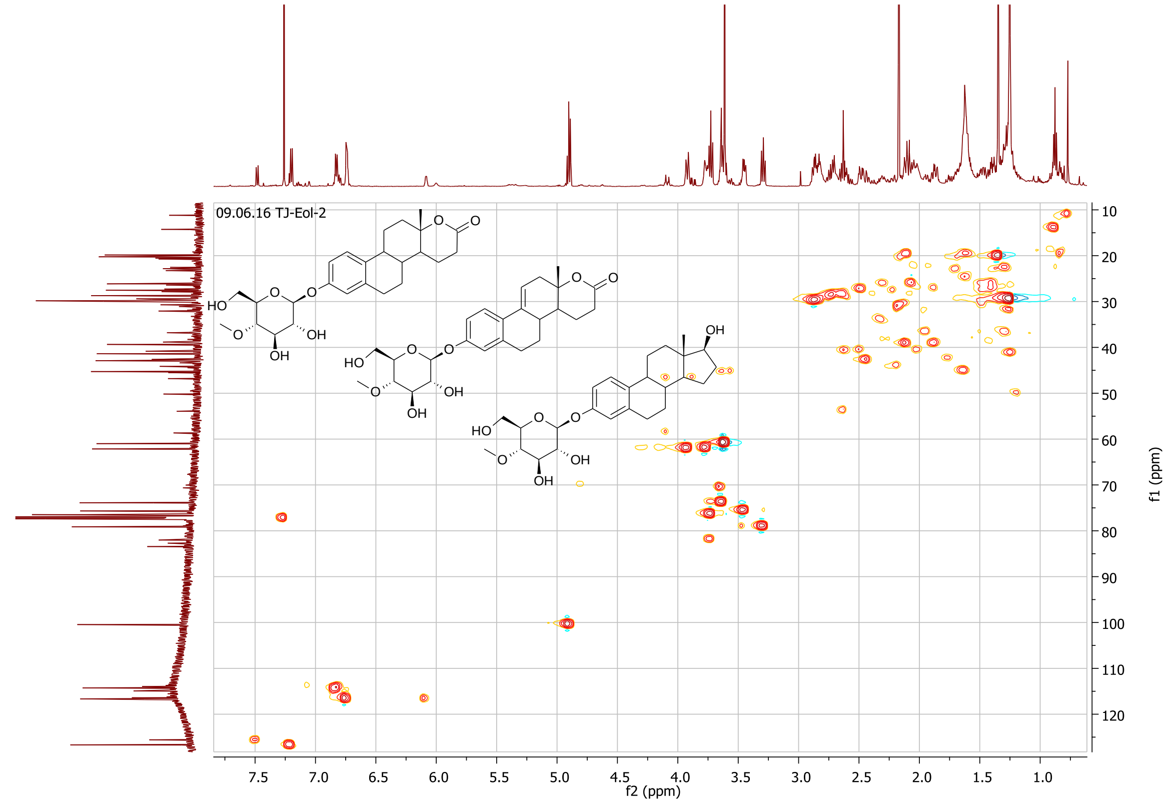

Fig.S31. ^{1}H NMR spectral of 3-(β-D-4’-O-methyloglucosyloxy)-17a-oxa-D-homo-estr-17-one (**9**), 3-(β-D-4’-O-methyloglucosyloxy)-17a-oxa-D-homo-estr-9-en-17-one (**10**) and 3,6β-dihydroxy-17a-oxa-D-homo-estrone (**7**) (DMSO-d_6, 600 MHz)

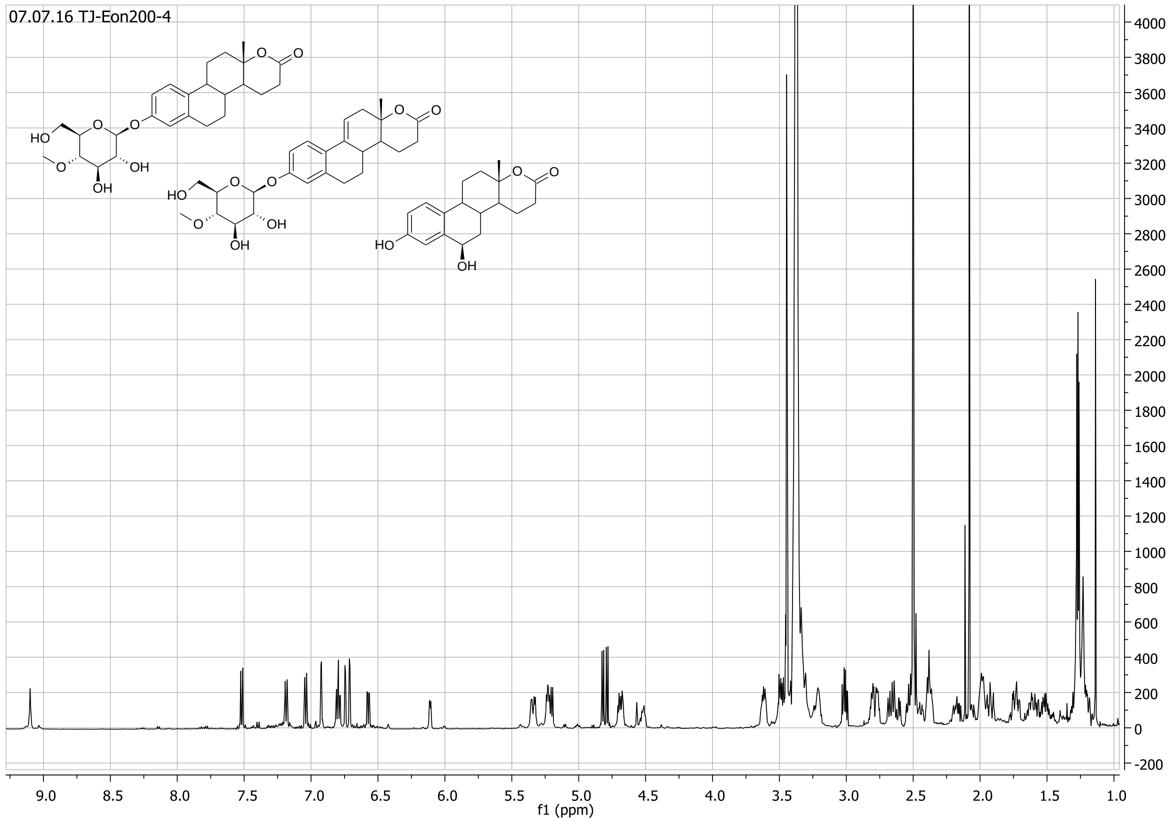

Fig.S32. Part of the ^{13}C NMR spectral of 3-(β-D-4’-O-methyloglucosyloxy)-17a-oxa-D-homo-estr-17-one (**9**), 3-(β-D-4’-O-methyloglucosyloxy)-17a-oxa-D-homo-estr-9-en-17-one (**10**) and 3,6β-dihydroxy-17a-oxa-D-homo-estrone (**7**) (DMSO-d_6, 151 MHz)

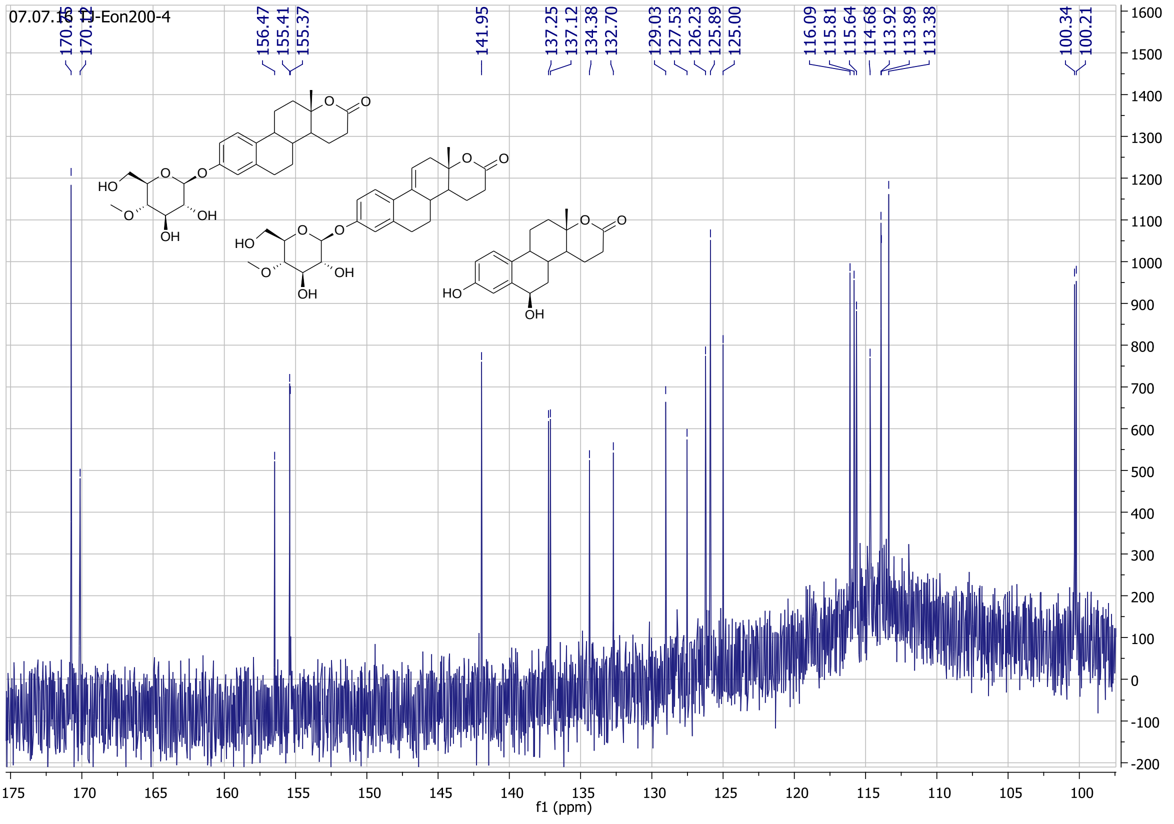

Fig.S33. Part of the ^{13}C NMR spectral of 3-(β-D-4'-O-methyloglucosyloxy)-17a-oxa-D-homo-estr-17-one (**9**), 3-(β-D-4'-O-methyloglucosyloxy)-17a-oxa-D-homo-estr-9-en-17-one (**10**) and 3,6β-dihydroxy-17a-oxa-D-homo-estrone (**7**) (DMSO-d_6, 151 MHz)

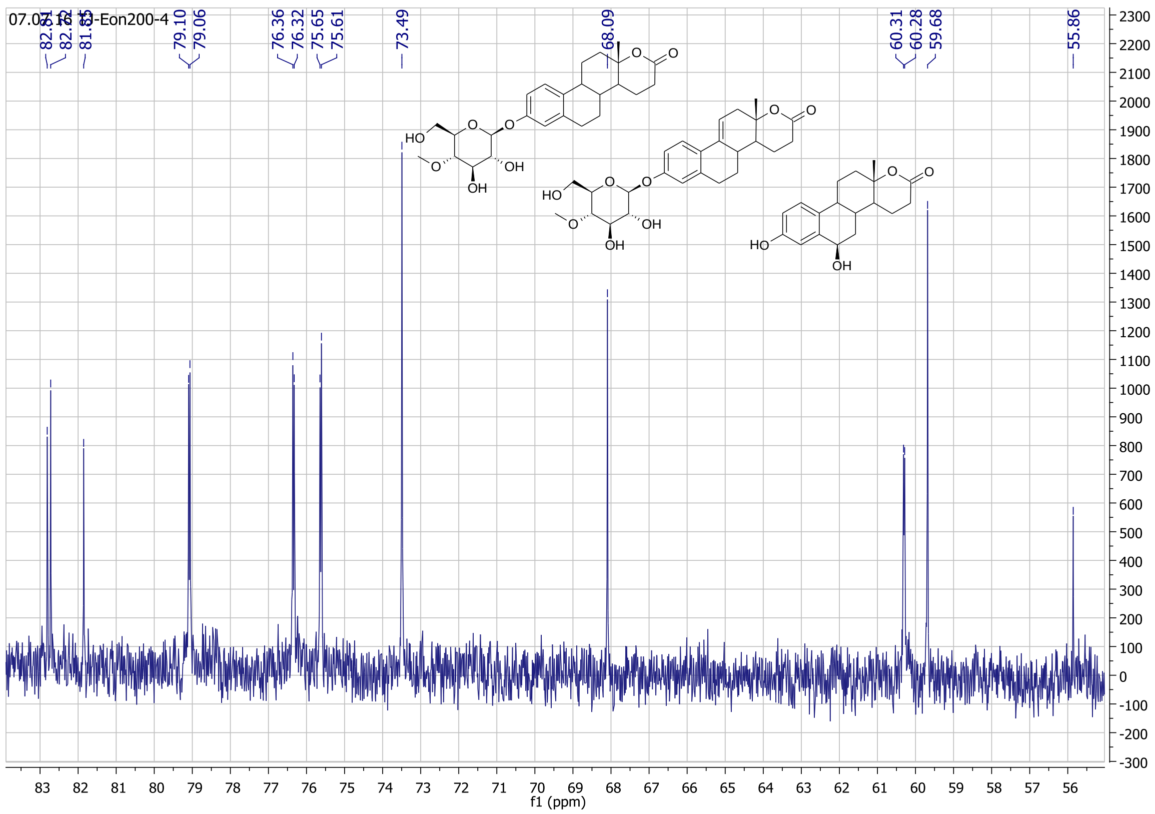

Fig.S34. Part of the ^{13}C NMR spectral of 3-(β-D-4'-O-methyloglucosyloxy)-17a-oxa-D-homo-estr-17-one (**9**), 3-(β-D-4'-O-methyloglucosyloxy)-17a-oxa-D-homo-estr-9-en-17-one (**10**) and 3,6β-dihydroxy-17a-oxa-D-homo-estrone (**7**) (DMSO-d_6, 151 MHz)

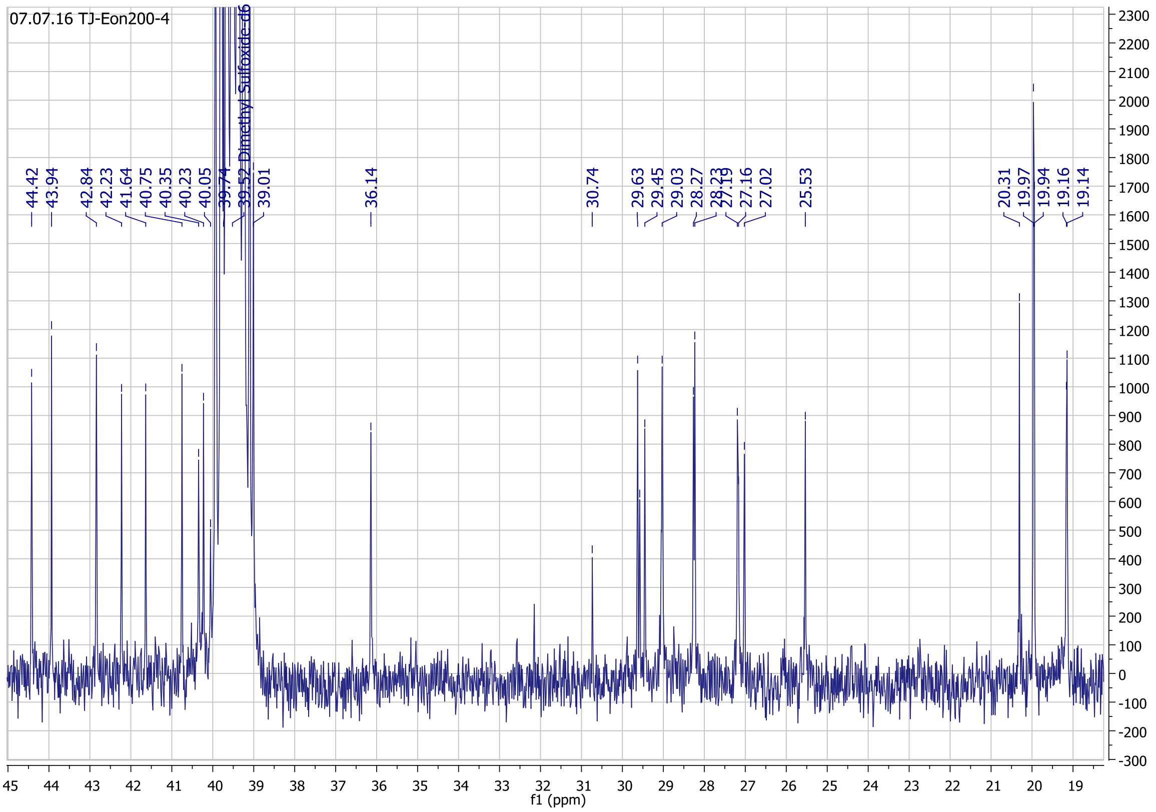

Fig.S35. ^{1}H NMR spectral of 3-O-(β-D-4'-O-methyloglucopyranosyl)-2-hydroxyestrone (**11**) (DMSO-d_6, 600 MHz)

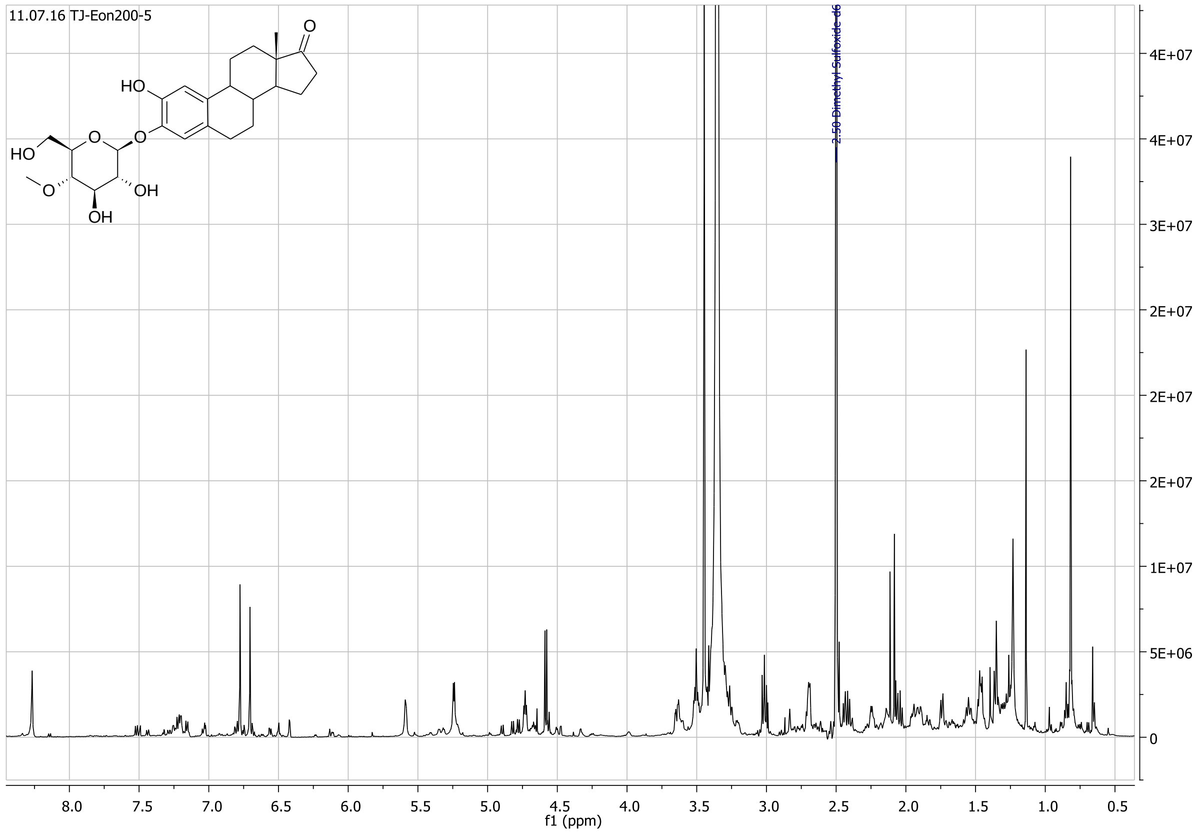

Fig.S36. ^{13}C NMR spectral of 3-O-(β-D-4'-O-methyloglucopyranosyl)-2-hydroxyestrone (**11**) (DMSO-d_6, 151 MHz)

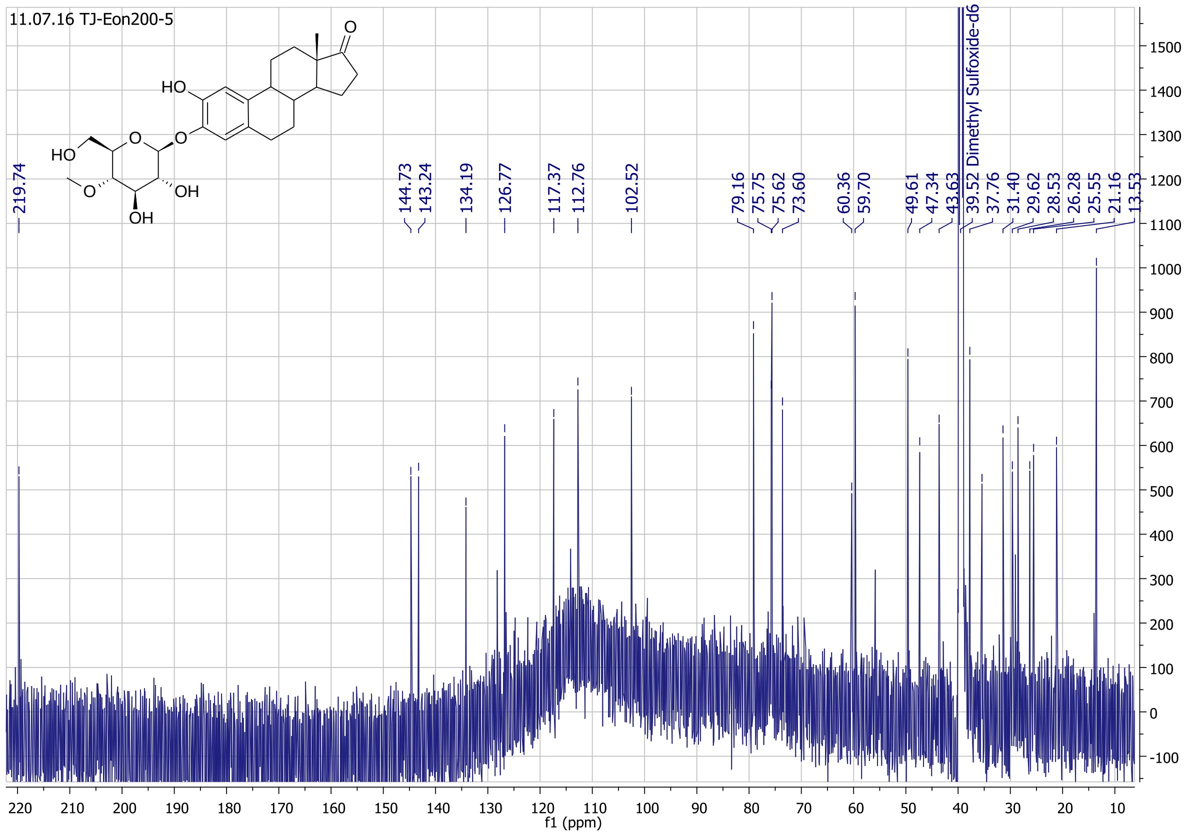

Fig.S37. HSQC spectral of 3-O-(β-D-4’-O-methyloglucopyranosyl)-2-hydroxyestrone (**11**) (DMSO-d_6, 151 MHz)

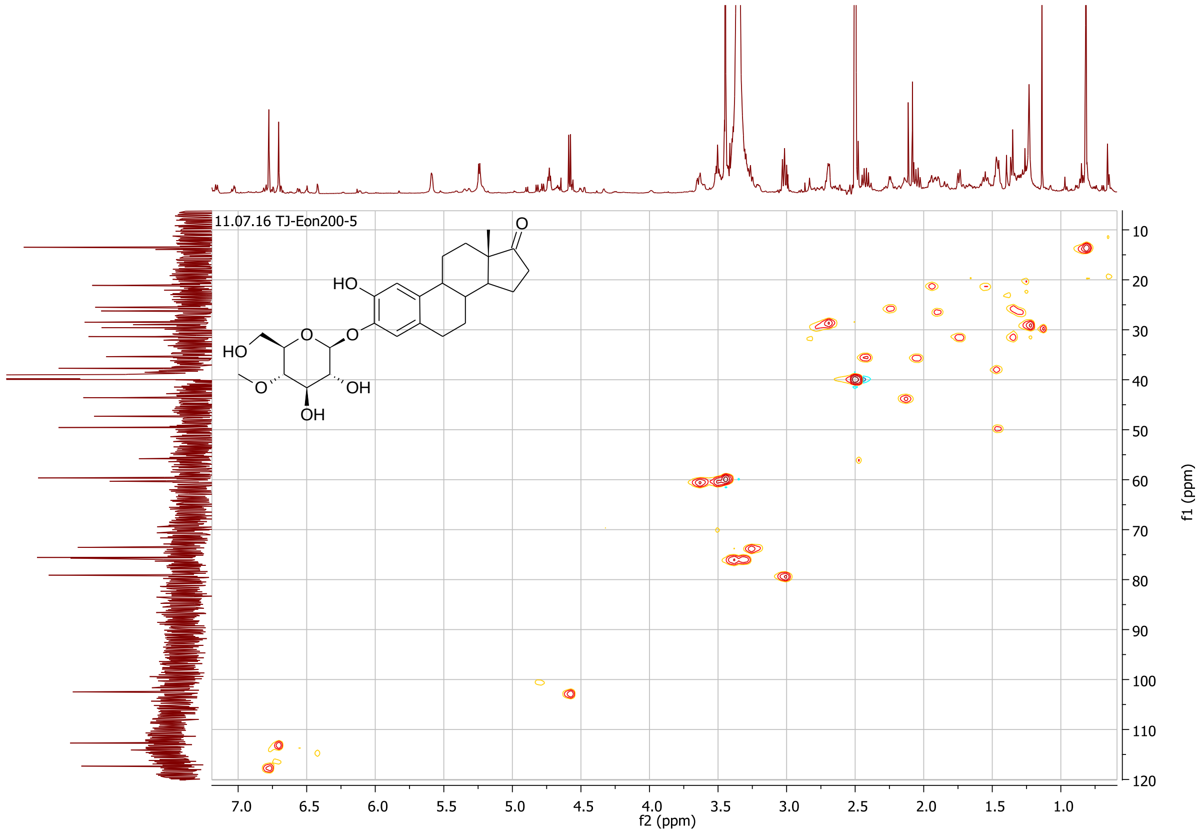

Fig.S38. COSY spectral of 3-O-(β-D-4’-O-methyloglucopyranosyl)-2-hydroxyestrone (**11**) (DMSO-d_6, 151 MHz)

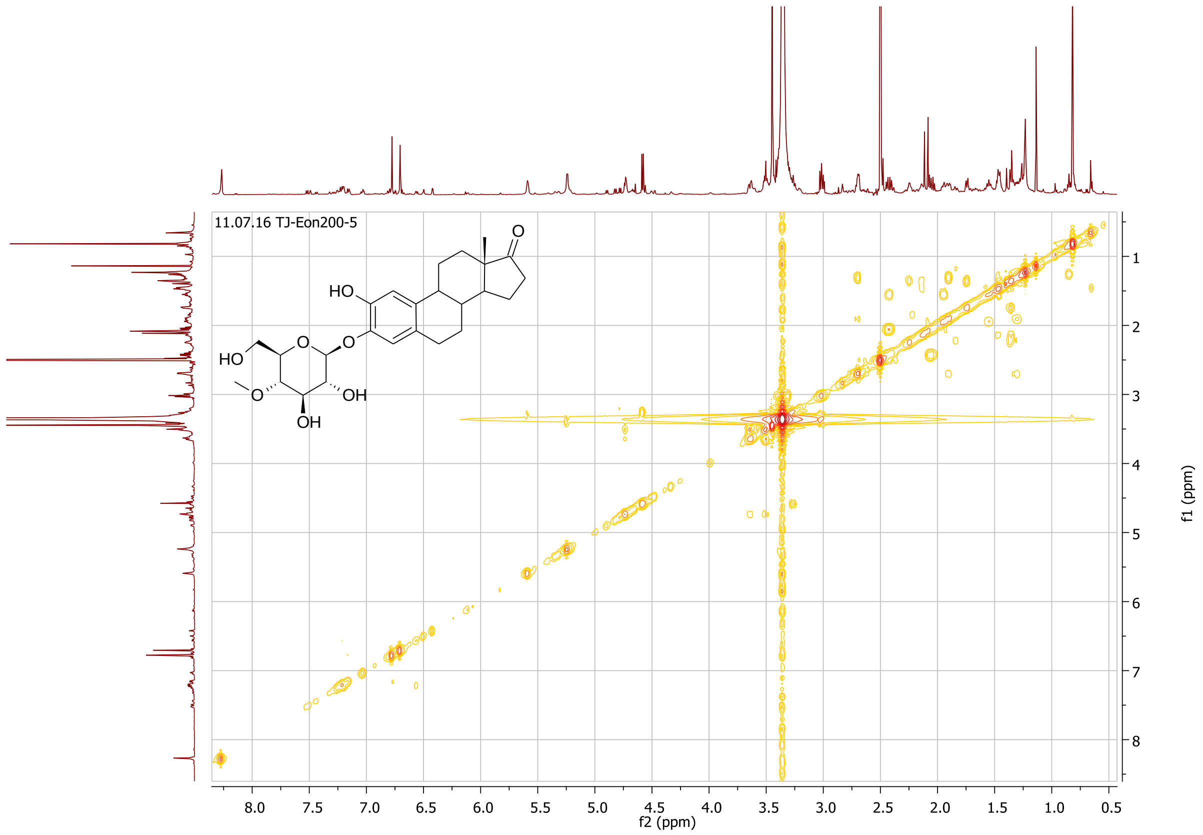

Fig.S39. ^{1}H NMR spectral of 3-(β-D-4’-O-methyloglucosyloxy)-estr-17β-ol (**12**) and 3-(β-D-4’-O-methyloglucosyloxy)-estr-9-en-17β-ol (**13**) (DMSO-d_6, 600 MHz)

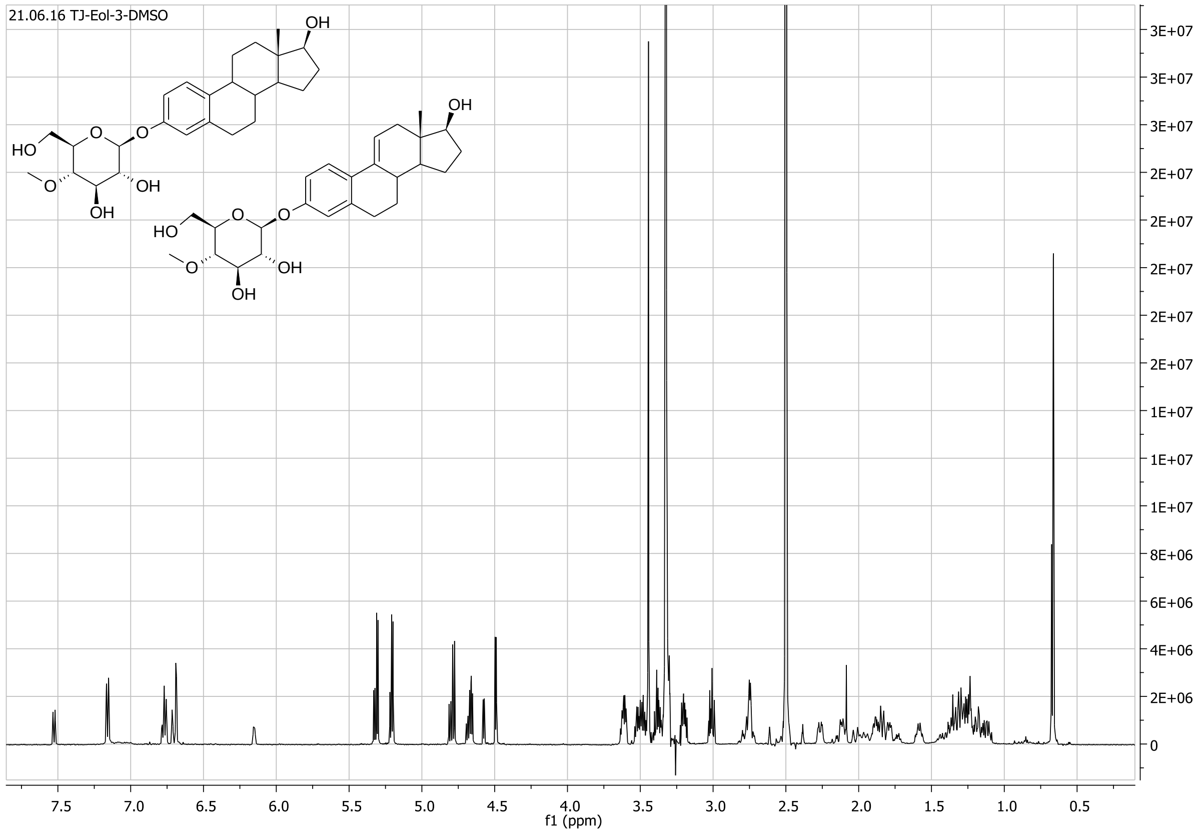

Fig.S40. Part of the ^{13}C NMR spectral of 3-(β-D-4’-O-methyloglucosyloxy)-estr-17β-ol (**12**) and 3-(β-D-4’-O-methyloglucosyloxy)-estr-9-en-17β-ol (**13**) (DMSO-d_6, 151 MHz)

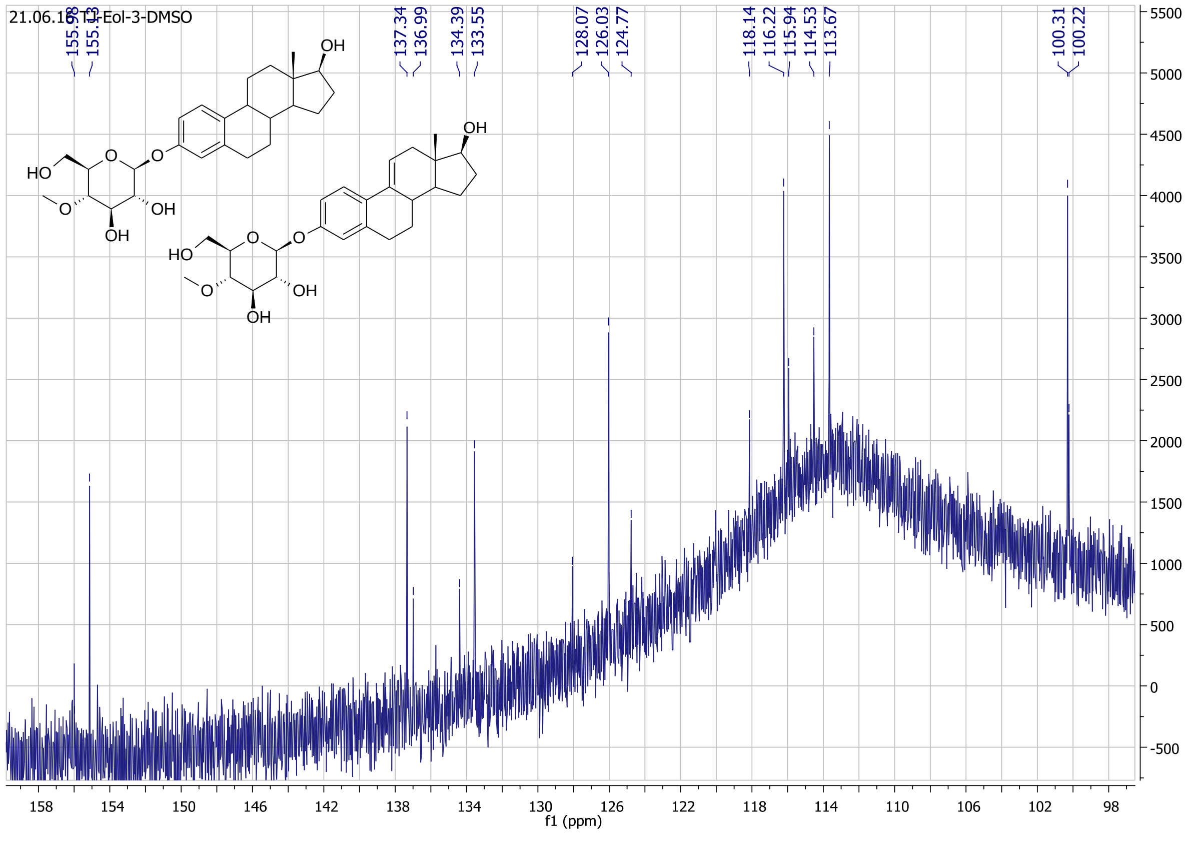

Fig.S41. Part of the ^{13}C NMR spectral of 3-(β-D-4’-O-methyloglucosyloxy)-estr-17β-ol (**12**) and 3-(β-D-4’-O-methyloglucosyloxy)-estr-9-en-17β-ol (**13**) (DMSO-d_6, 151 MHz)

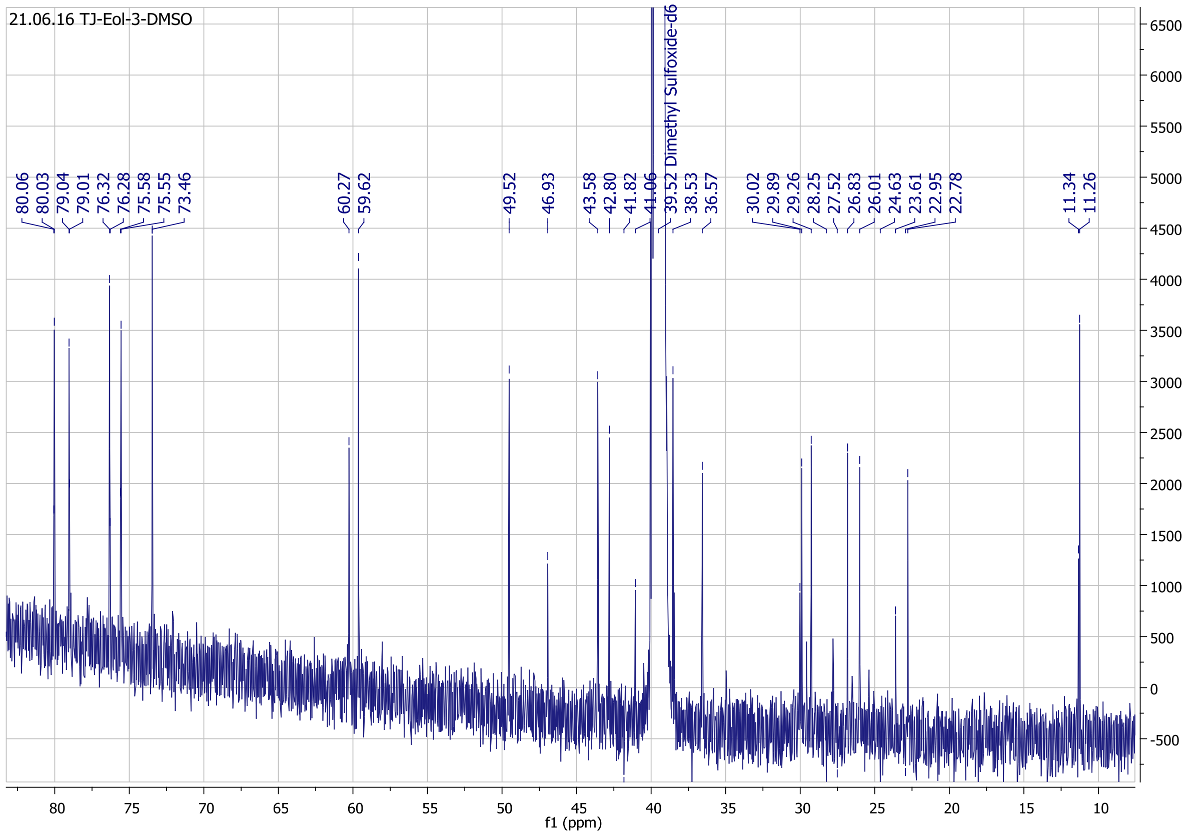

Fig.S42. HSQC spectral of 3-(β-D-4’-O-methyloglucosyloxy)-estr-17β-ol (**12**) and 3-(β-D-4’-O-methyloglucosyloxy)-estr-9-en-17β-ol (**13**) (DMSO-d_6, 151 MHz)

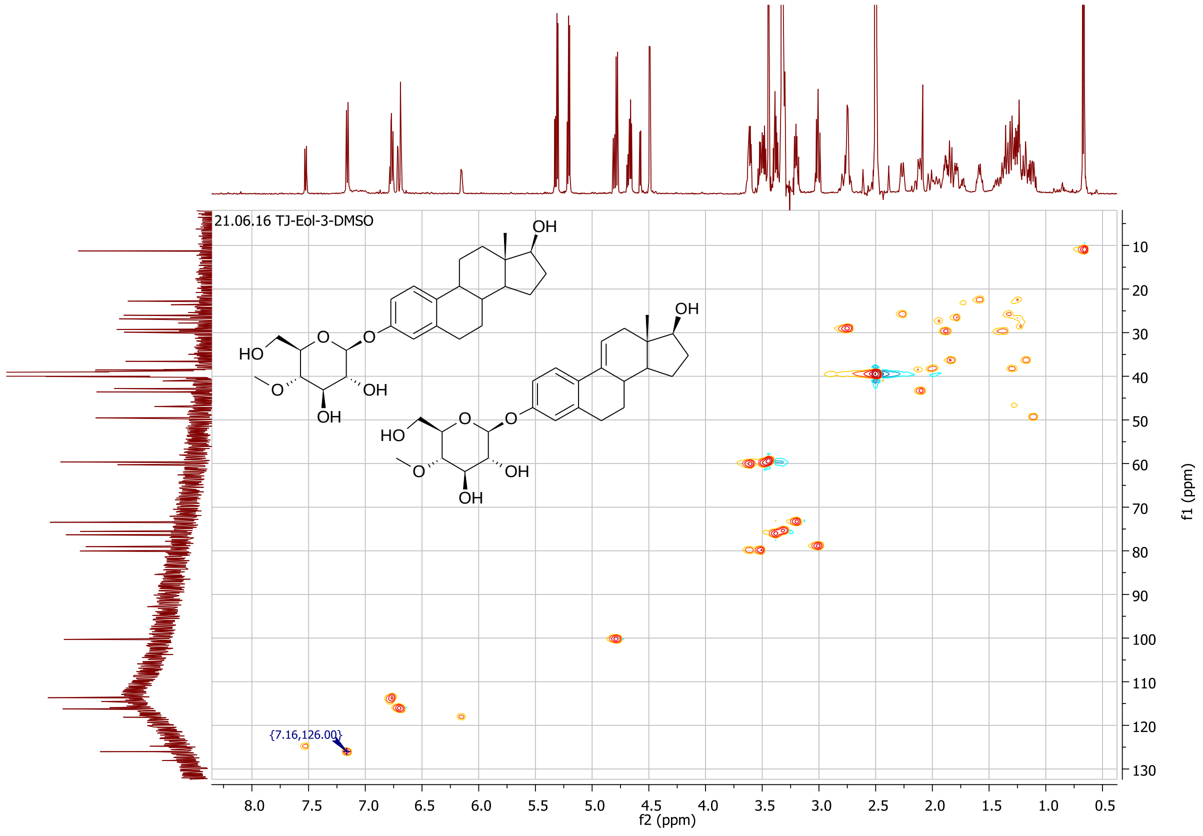

Fig.S43. COSY spectral of 3-(β-D-4’-O-methyloglucosyloxy)-estr-17β-ol (**12**) and 3-(β-D-4’-O-methyloglucosyloxy)-estr-9-en-17β-ol (**13**) (DMSO-d_6, 151 MHz)

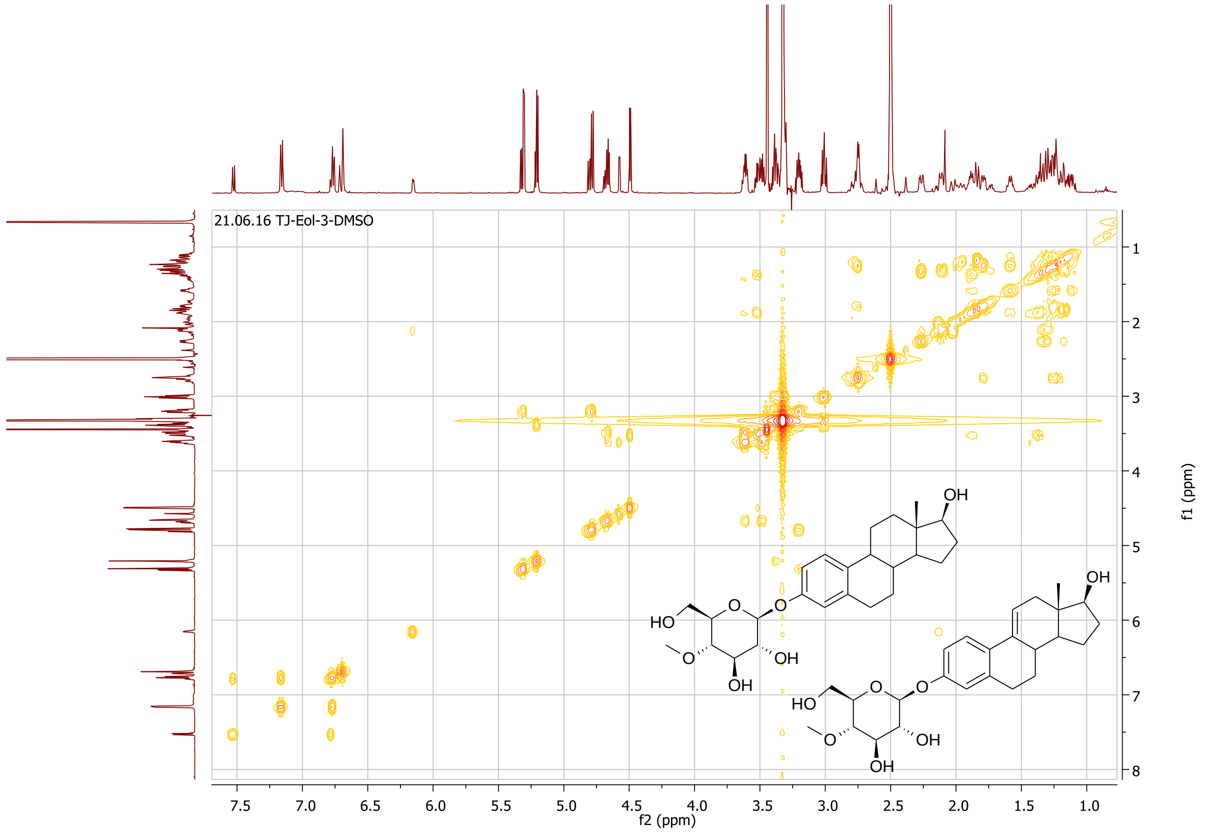

Fig.S44. ^{1}H NMR spectral of estra-3,6β,17β-triol (**6**) and 3-(β-D-4’-O-methyloglucosyloxy)-estr-17β-ol (**12**) (DMSO-d_6, 600 MHz)

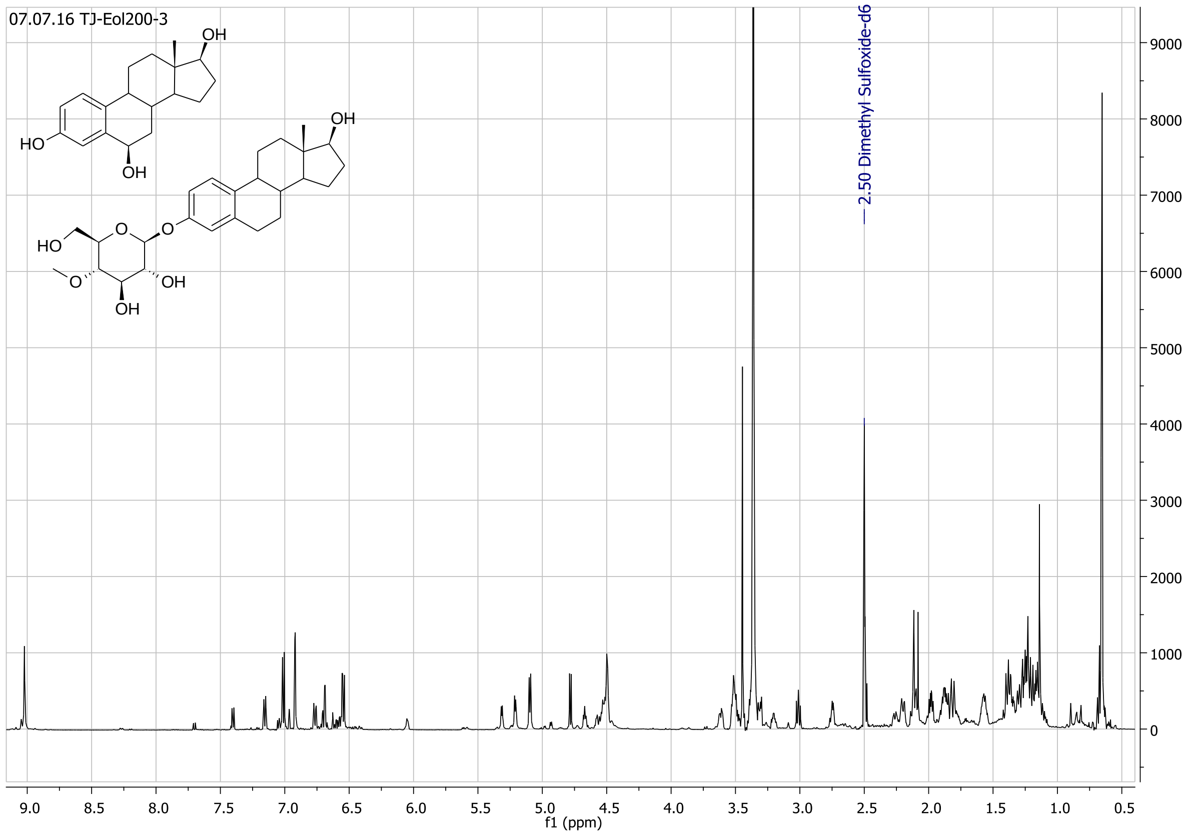

Fig.S45. Part of the ^{13}C NMR spectral estra-3,6β,17β-triol (**6**) and 3-(β-D-4'-O-methyloglucosyloxy)-estr-17β-ol (**12**) (DMSO-d_6, 600 MHz)

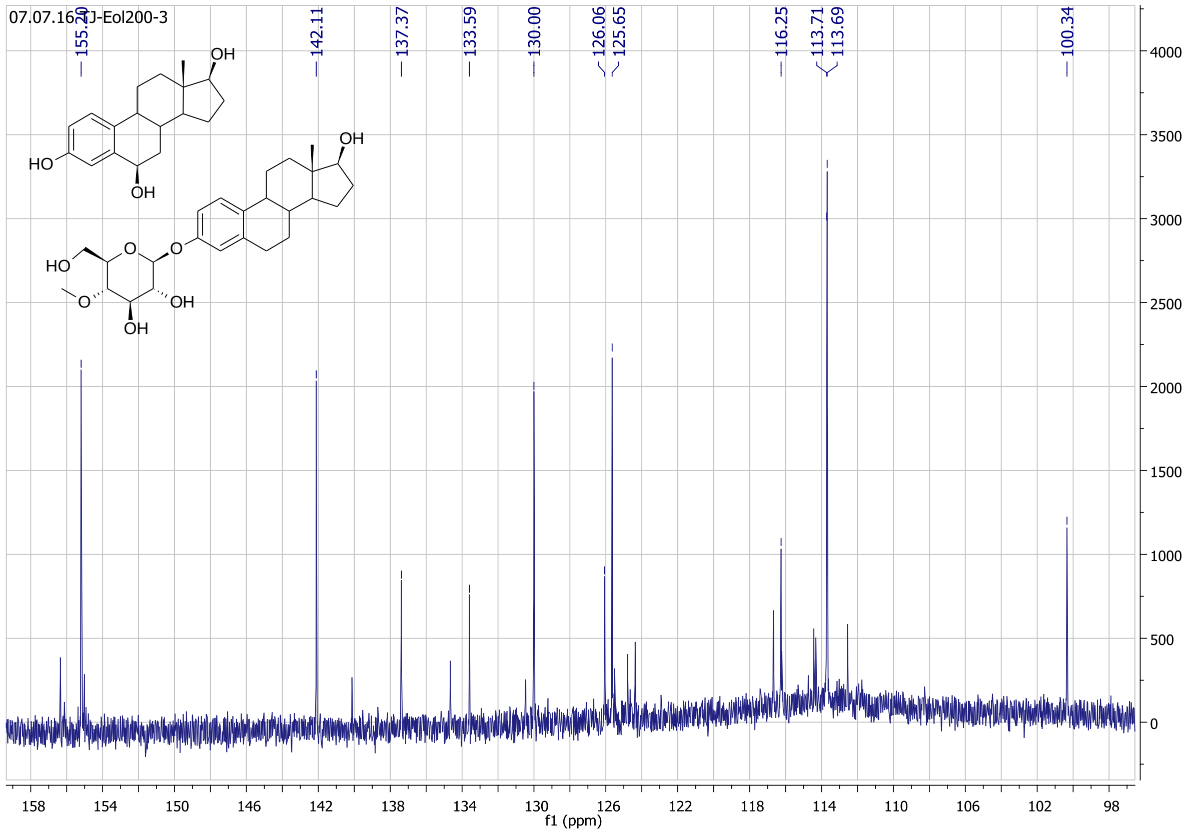

Fig.S46. Part of the ^{13}C NMR spectral of estra-3,6β,17β-triol (**6**) and 3-(β-D-4'-O-methyloglucosyloxy)-estr-17β-ol (**12**) (DMSO-d_6, 600 MHz)

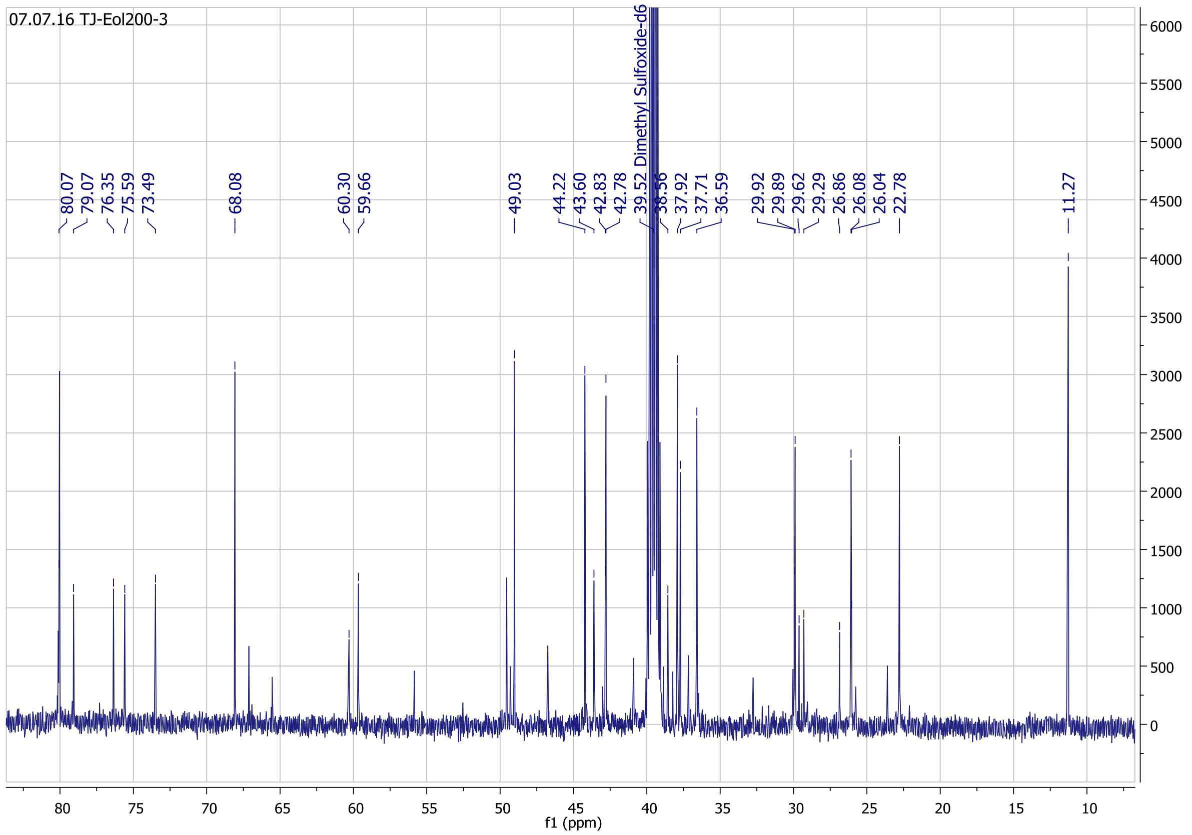

Fig.S47. HSQC spectral of estra-3,6β,17β-triol (**6**) and 3-(β-D-4'-O-methyloglucosyloxy)-estr-17β-ol (**12**) (DMSO-d_6, 600 MHz)

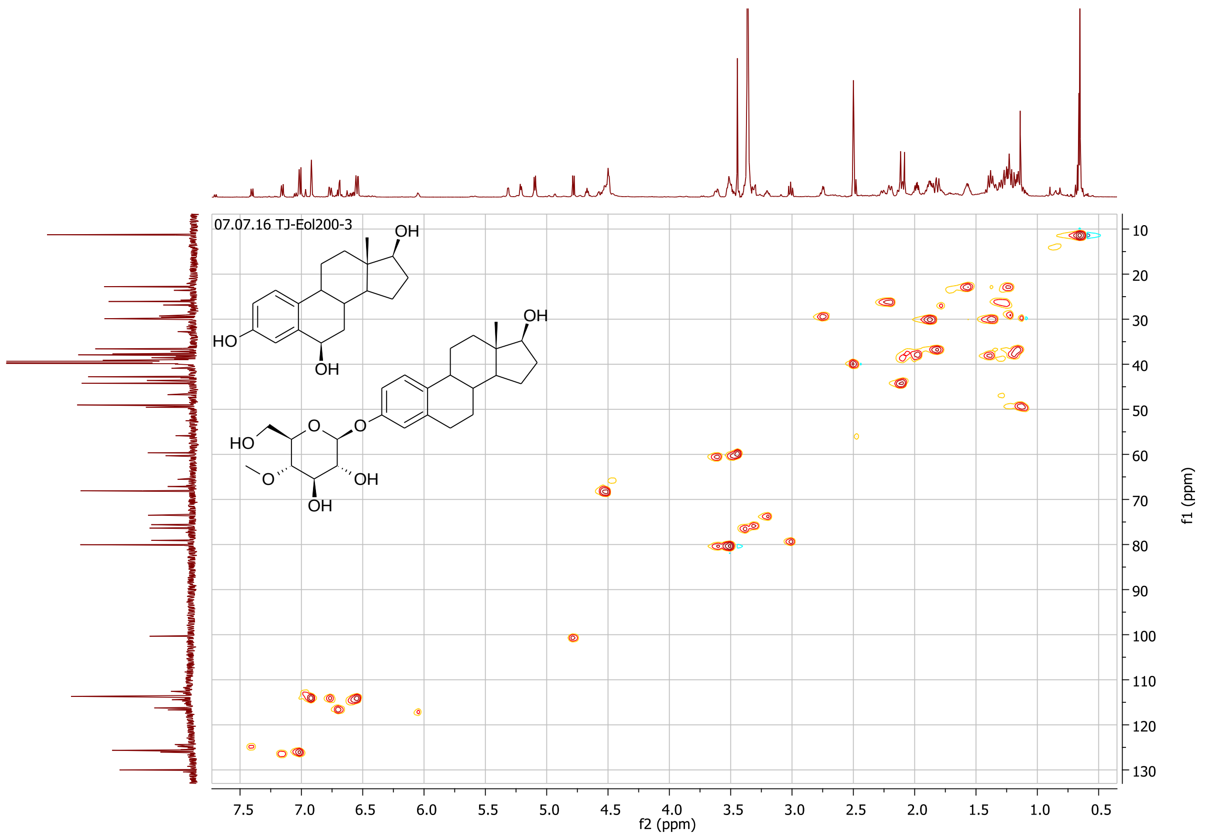

Fig.S48. ^{1}H NMR spectral of 3-(β-D-4'-O-methyloglucosyloxy)-estr-17β-ol (**12**) and 3-(β-D-4'-O-methyloglucosyloxy)-estr-9-en-17β-ol (**13**) (DMSO-d_6, 600 MHz)

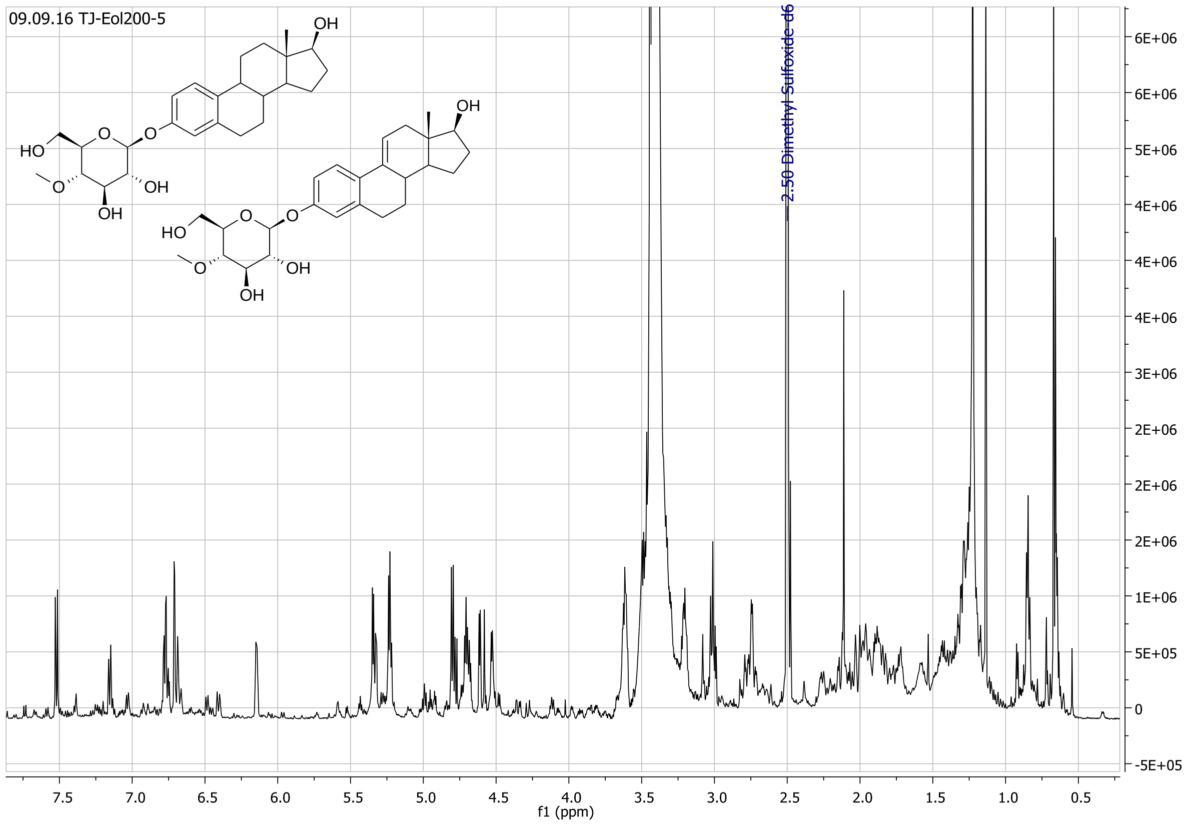

Fig.S49. Part of the ^{13}C NMR spectral of 3-(β-D-4’-O-methyloglucosyloxy)-estr-17β-ol (**12**) and 3-(β-D-4’-O-methyloglucosyloxy)-estr-9-en-17β-ol (**13**) (DMSO-d_6, 151 MHz)

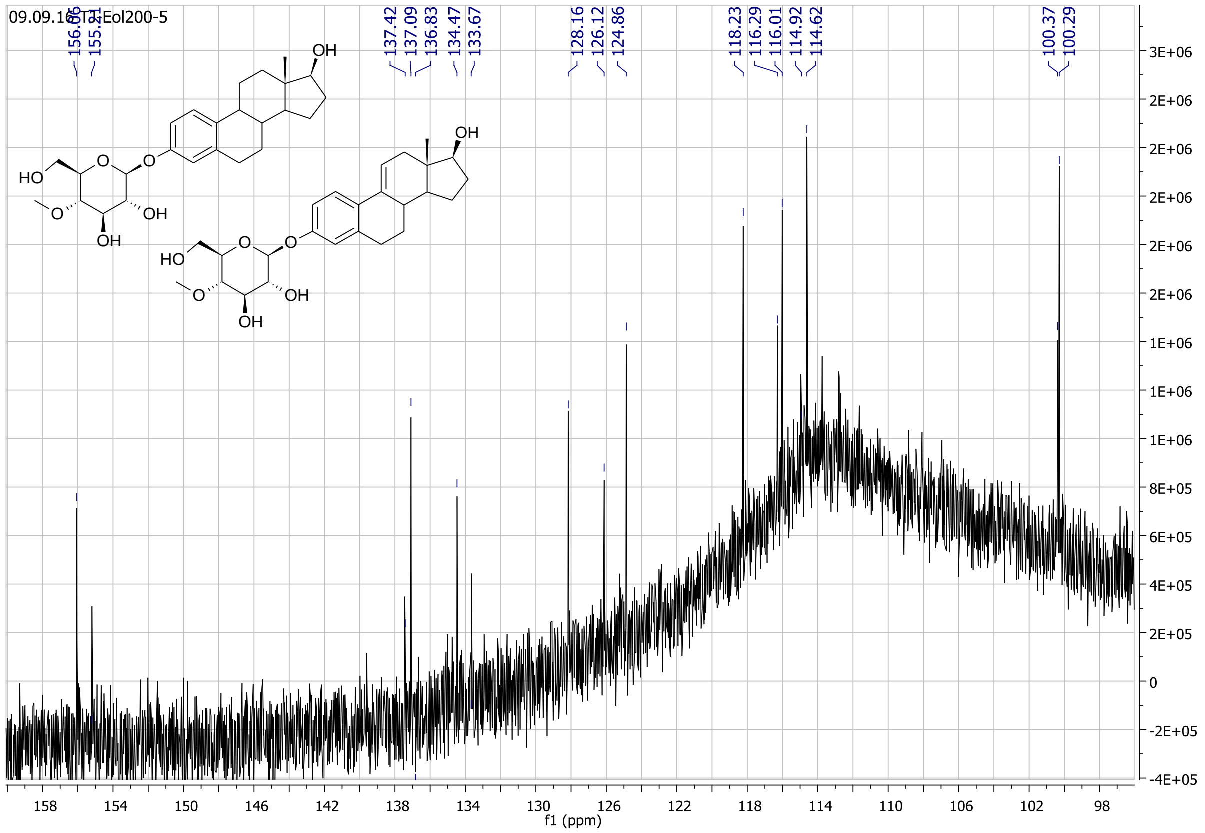

Fig.S50. Part of the ^{13}C NMR spectral of 3-(β-D-4’-O-methyloglucosyloxy)-estr-17β-ol (**12**) and 3-(β-D-4’-O-methyloglucosyloxy)-estr-9-en-17β-ol (**13**) (DMSO-d_6, 151 MHz)

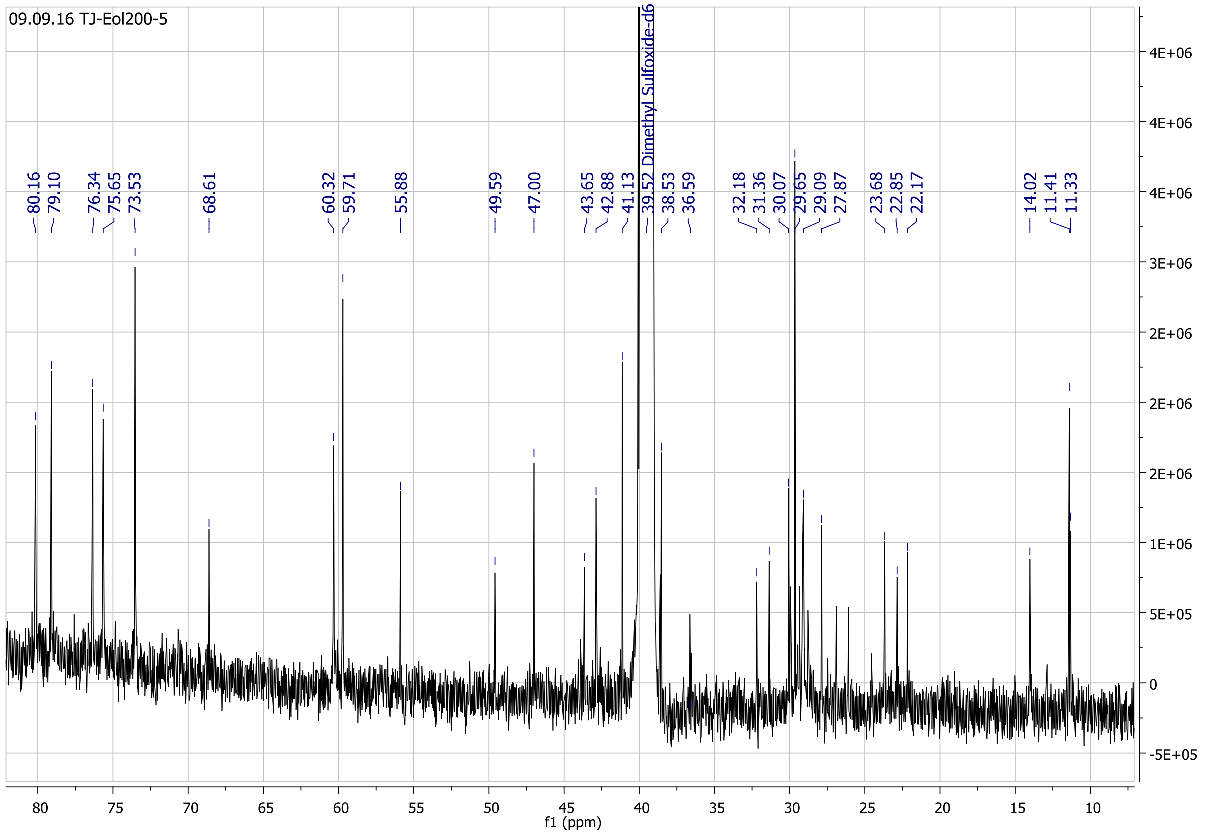

Fig.S51. HSQC spectral of 3-(β-D-4’-O-methyloglucosyloxy)-estr-17β-ol (**12**) and 3-(β-D-4’-O-methyloglucosyloxy)-estr-9-en-17β-ol (**13**) (DMSO-d_6, 151 MHz)

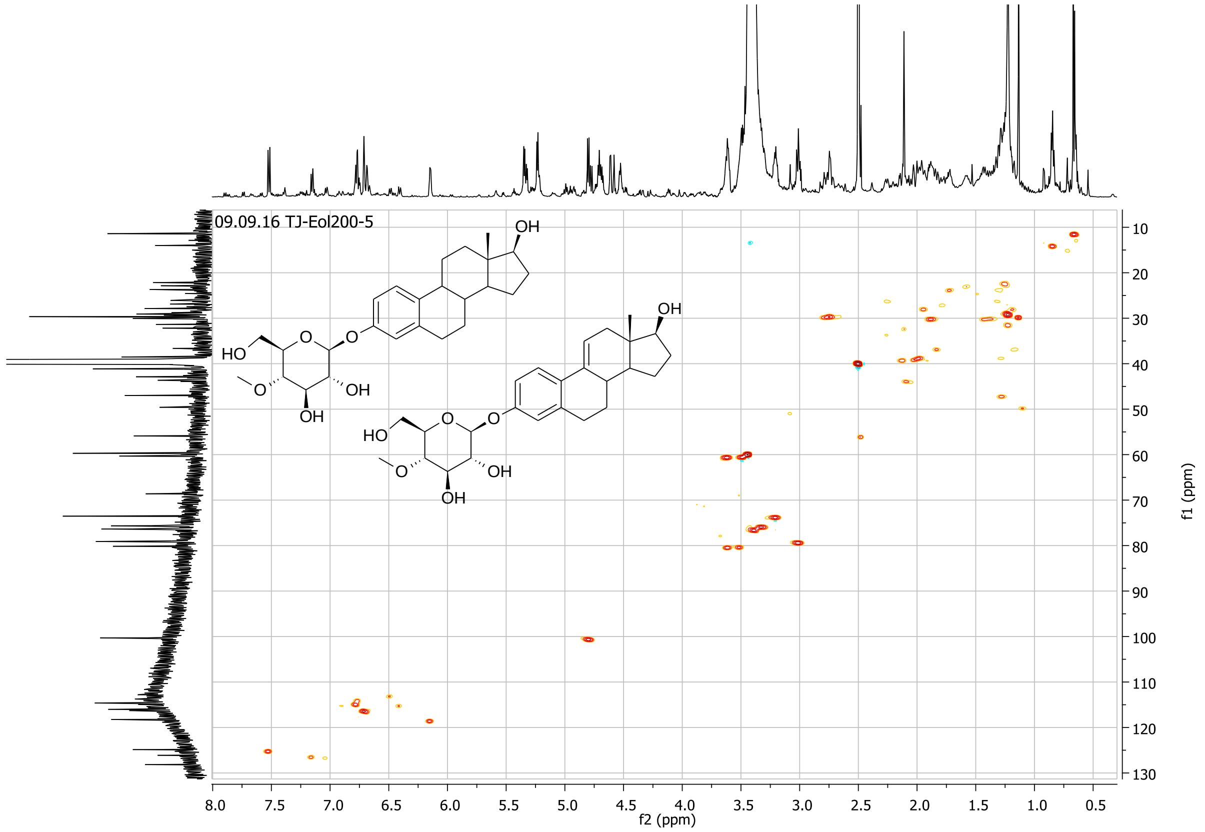

Fig.S52. ^{1}H NMR spectral of 3,6β-dihydroxy-17a-oxa-D-homo-estrone (**7**), 3-(β-D-4’-O-methyloglucosyloxy)-estrone (**8**) and 3-(β-D-4’-O-methyloglucosyloxy)-estr-9-en-17-on (**14**) (DMSO-d_6, 600 MHz)

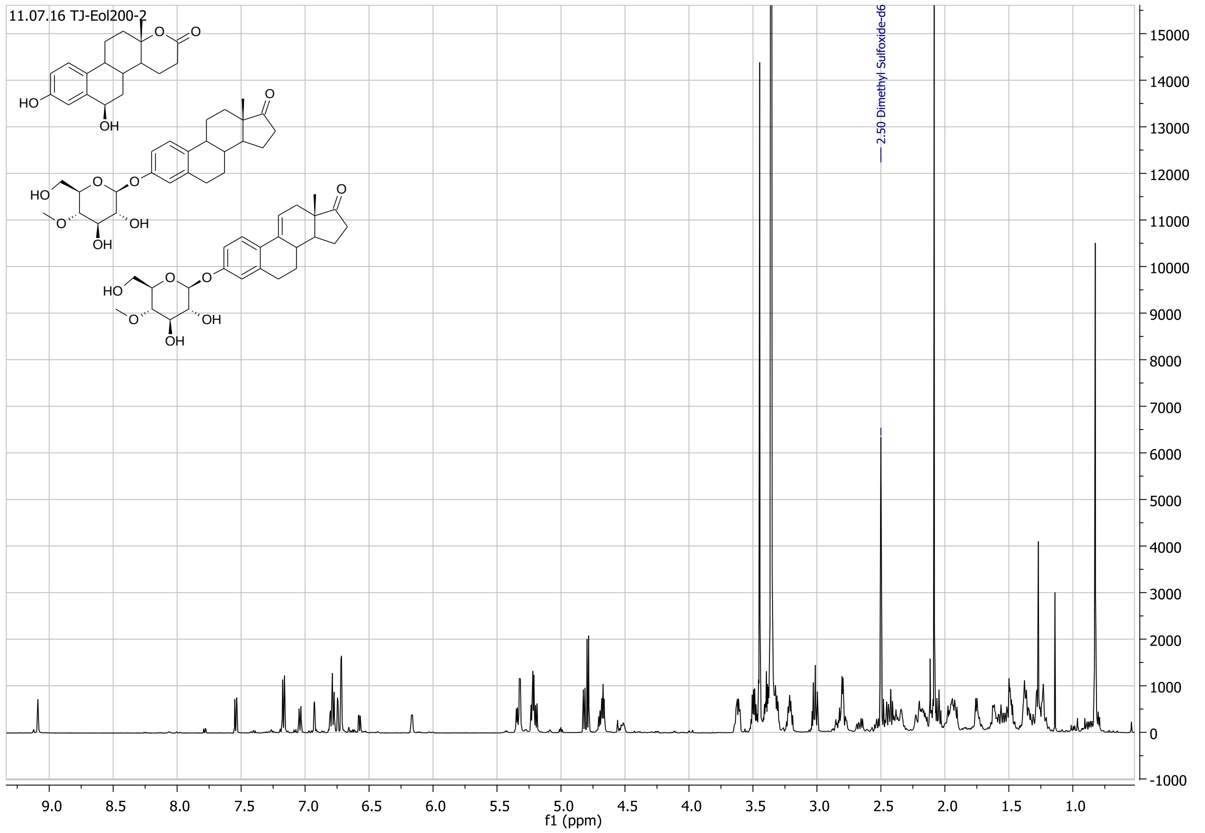

Fig.S53. Part of the ^{13}C NMR spectral of 3,6β-dihydroxy-17a-oxa-D-homo-estrone (**7**), 3-(β-D-4’-O-methyloglucosyloxy)-estrone (**8**) and 3-(β-D-4’-O-methyloglucosyloxy)-estr-9-en-17-on (**14**) (DMSO-d_6, 600 MHz)

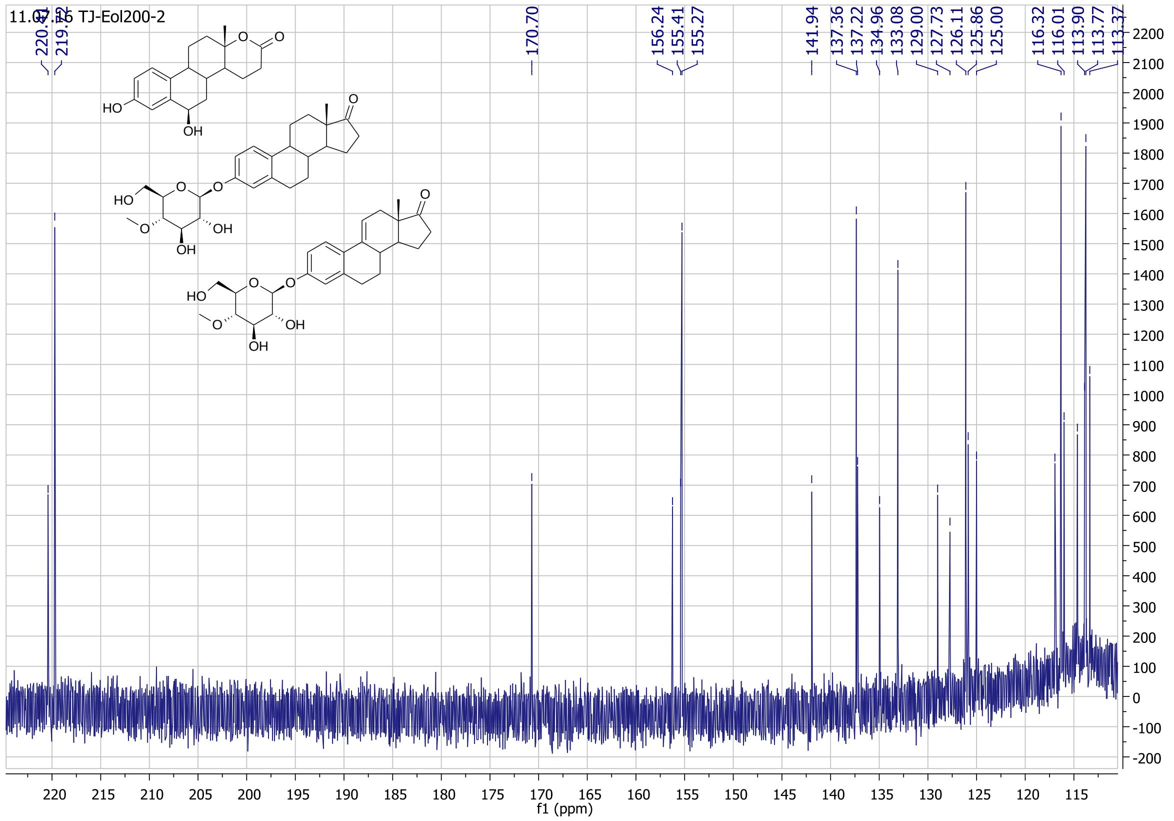

Fig.S54. Part of the ^{13}C NMR spectral of 3,6β-dihydroxy-17a-oxa-D-homo-estrone (**7**), 3-(β-D-4’-O-methyloglucosyloxy)-estrone (**8**) and 3-(β-D-4’-O-methyloglucosyloxy)-estr-9-en-17-on (**14**) (DMSO-d_6, 600 MHz)

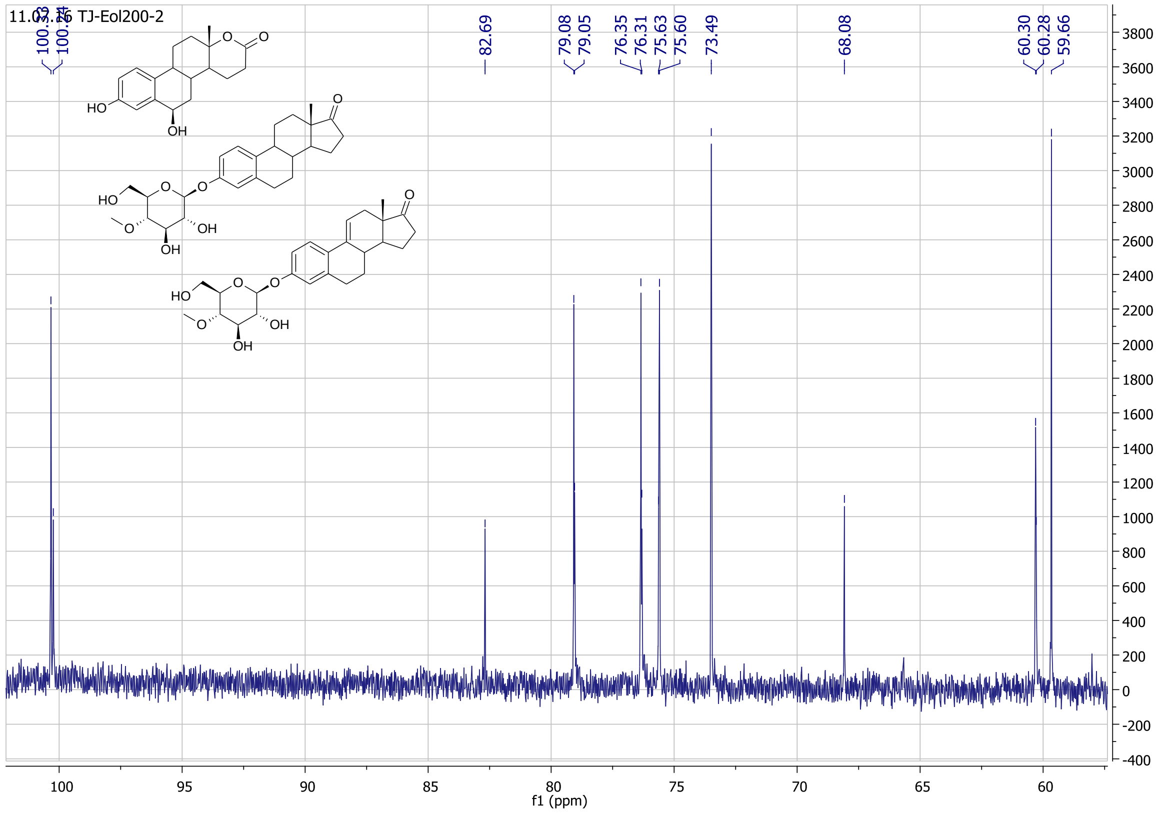

Fig.S55. Part of the ^{13}C NMR spectral of 3,6β-dihydroxy-17a-oxa-D-homo-estrone (**7**), 3-(β-D-4'-O-methyloglucosyloxy)-estrone (**8**) and 3-(β-D-4'-O-methyloglucosyloxy)-estr-9-en-17-on (**14**) (DMSO-d_6, 600 MHz)

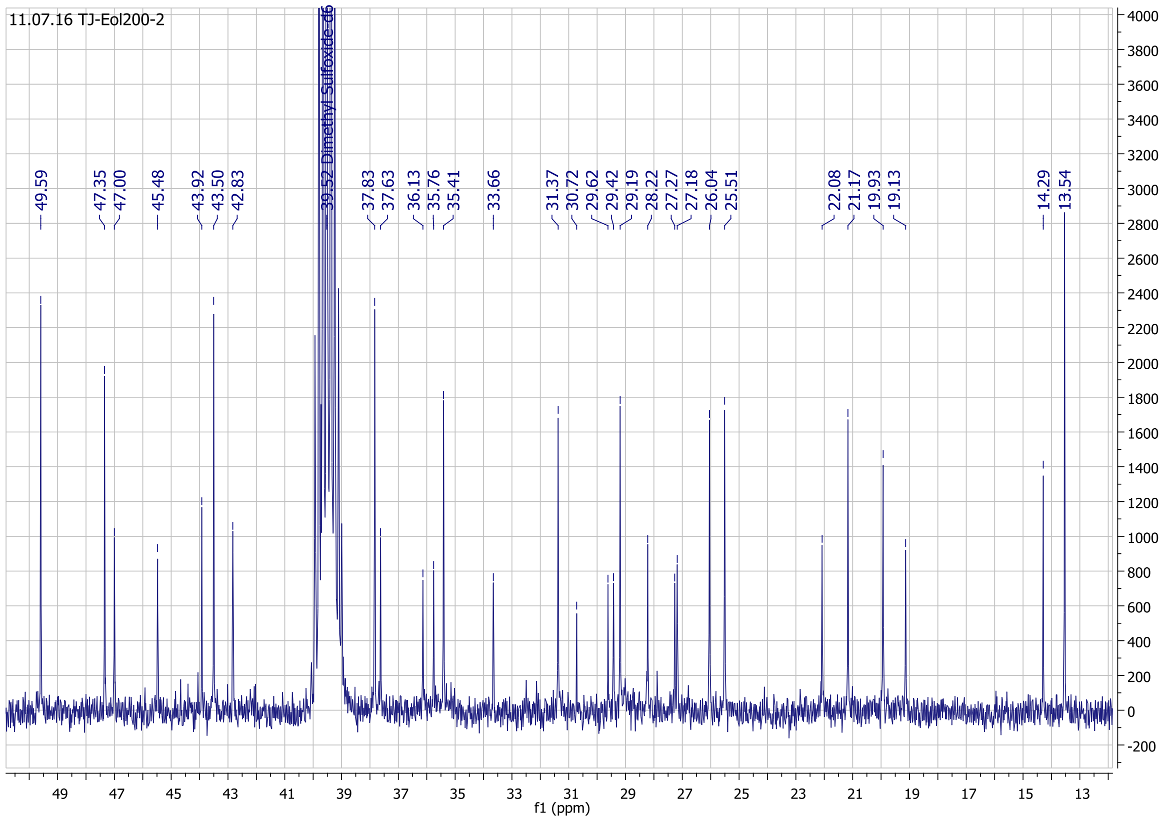

Fig.S56. HSQC spectral of 3,6β-dihydroxy-17a-oxa-D-homo-estrone (**7**), 3-(β-D-4'-O-methyloglucosyloxy)-estrone (**8**) and 3-(β-D-4'-O-methyloglucosyloxy)-estr-9-en-17-on (**14**) (DMSO-d_6, 600 MHz)

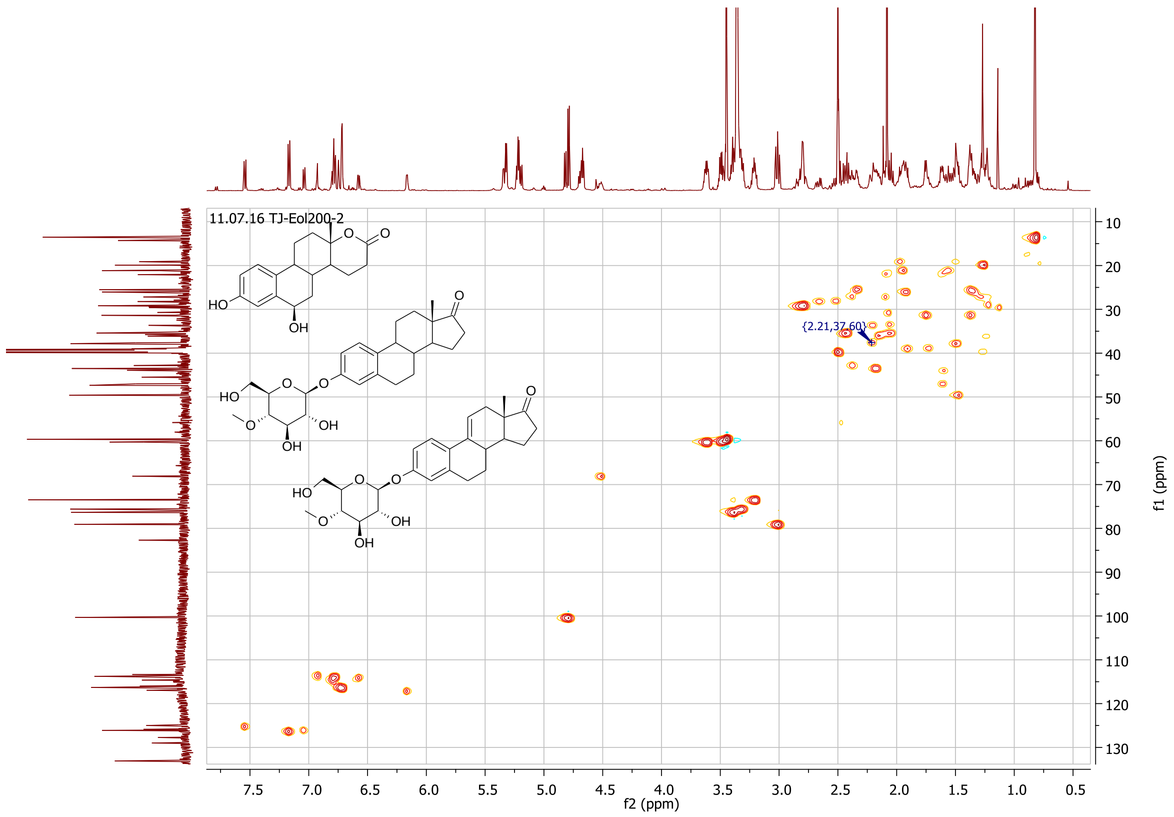

Fig.S57. ^{1}H NMR spectral of 17-ethynyloestra-3,6β,17β-triol (**15**) (DMSO-d_6, 600 MHz)

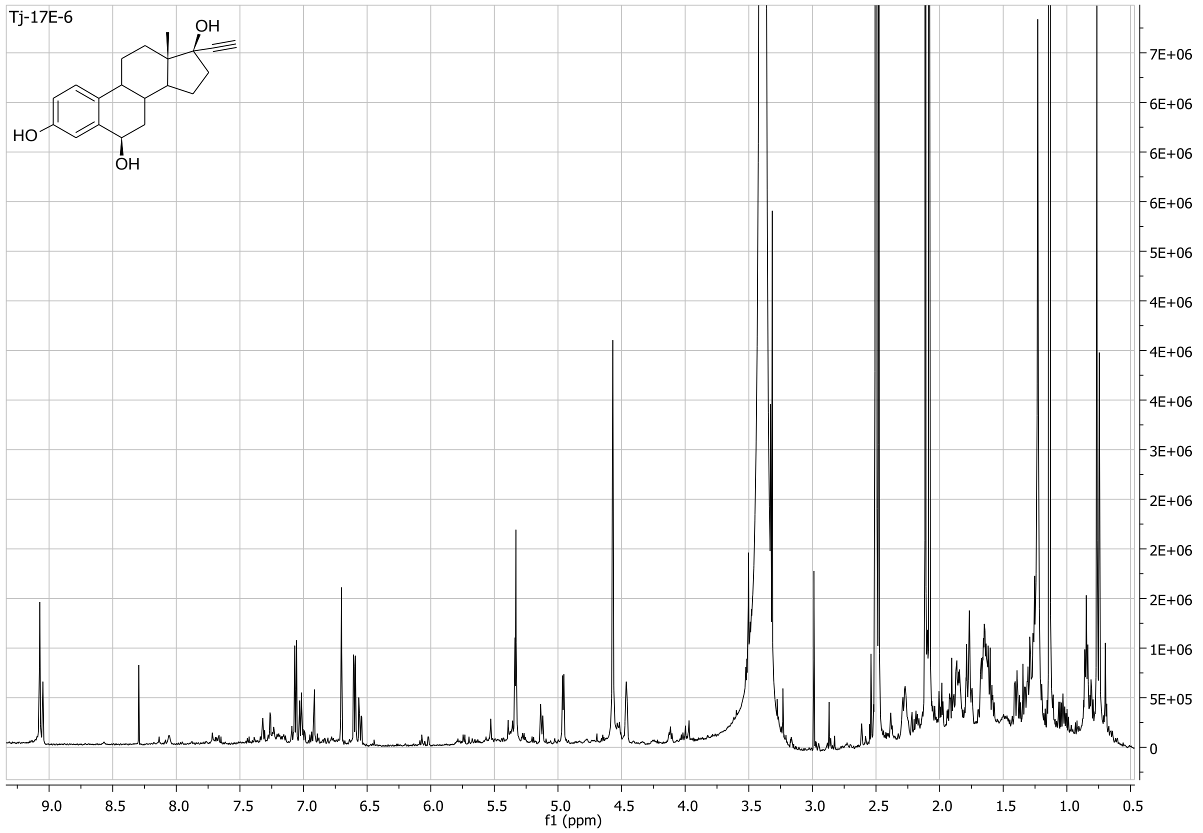

Fig.S58. ^{13}C NMR spectral of 17-ethynyloestra-3,6β,17β-triol (**15**) (DMSO-d_6, 151 MHz)

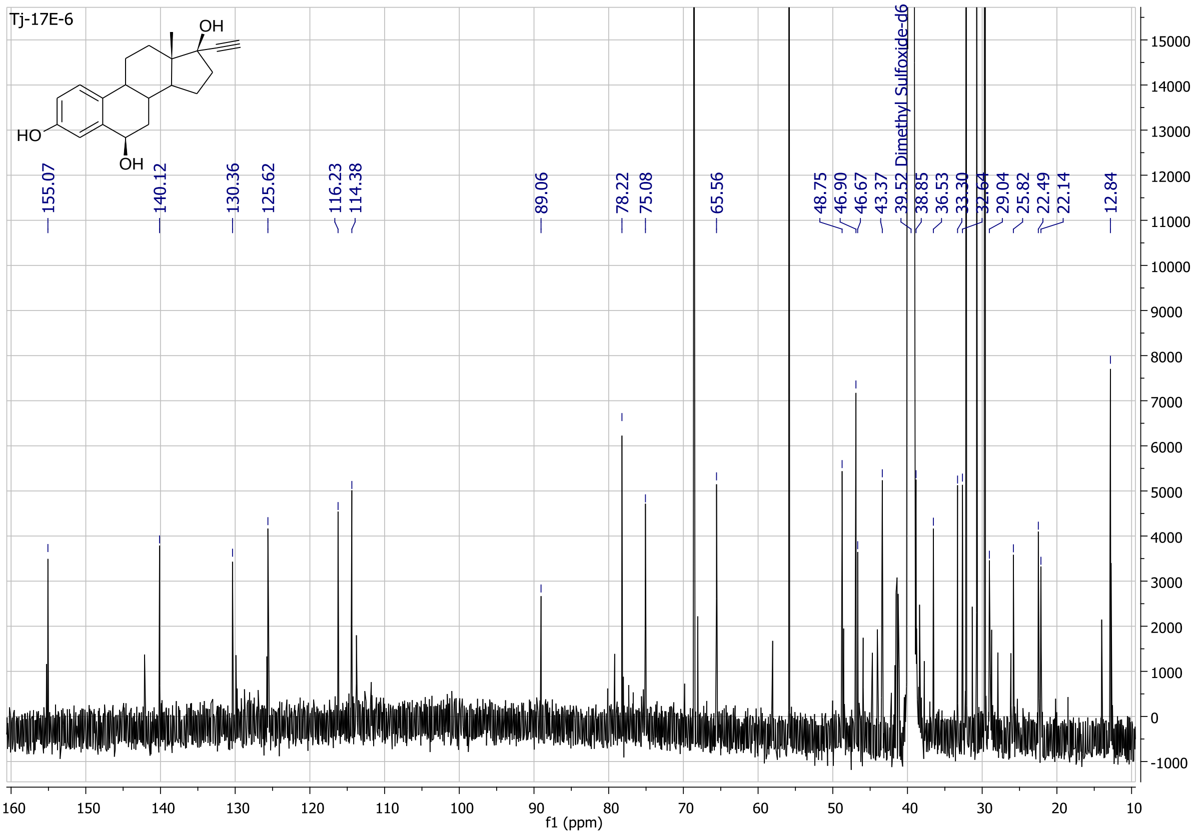

Fig.S59. HSQC spectral of 17-ethynyloestra-3,6β,17β-triol (**15**) (DMSO-d_6, 151 MHz)

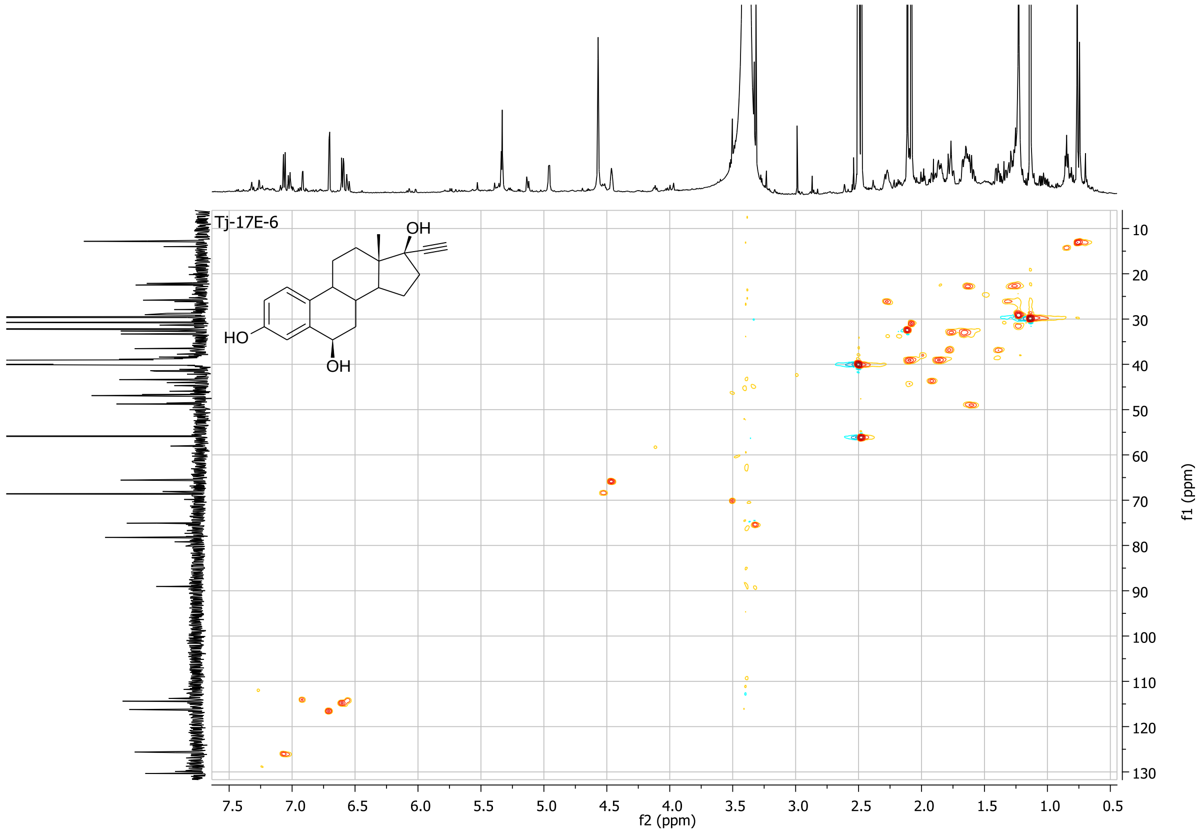

Fig.S60. COSY spectral of 17-ethynyloestra-3,6β,17β-triol (**15**) (DMSO-d_6, 151 MHz)

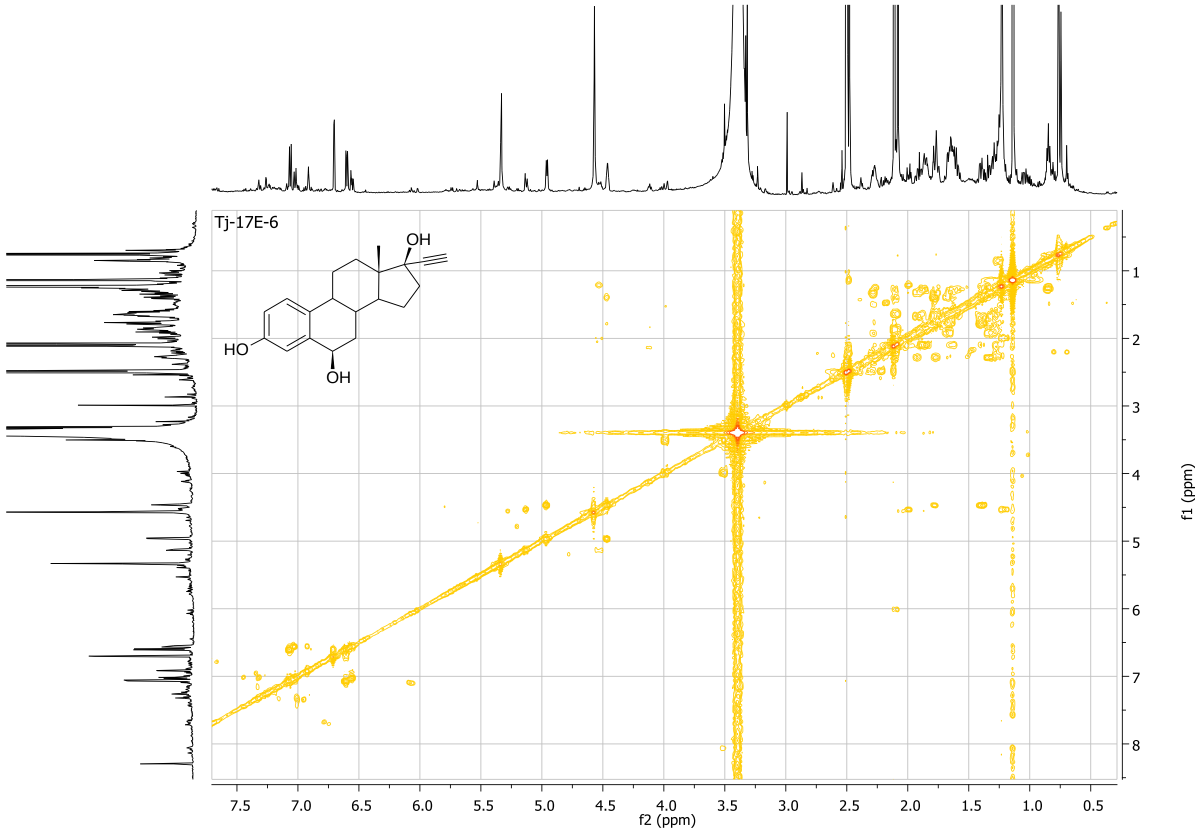

Fig.S61. ^{1}H NMR spectral of 3-(β-D-4'-O-methyloglucosyloxy)-17-ethynyloestr-17β-ol (**16**) (DMSO-*d*$_6$, 600 MHz)

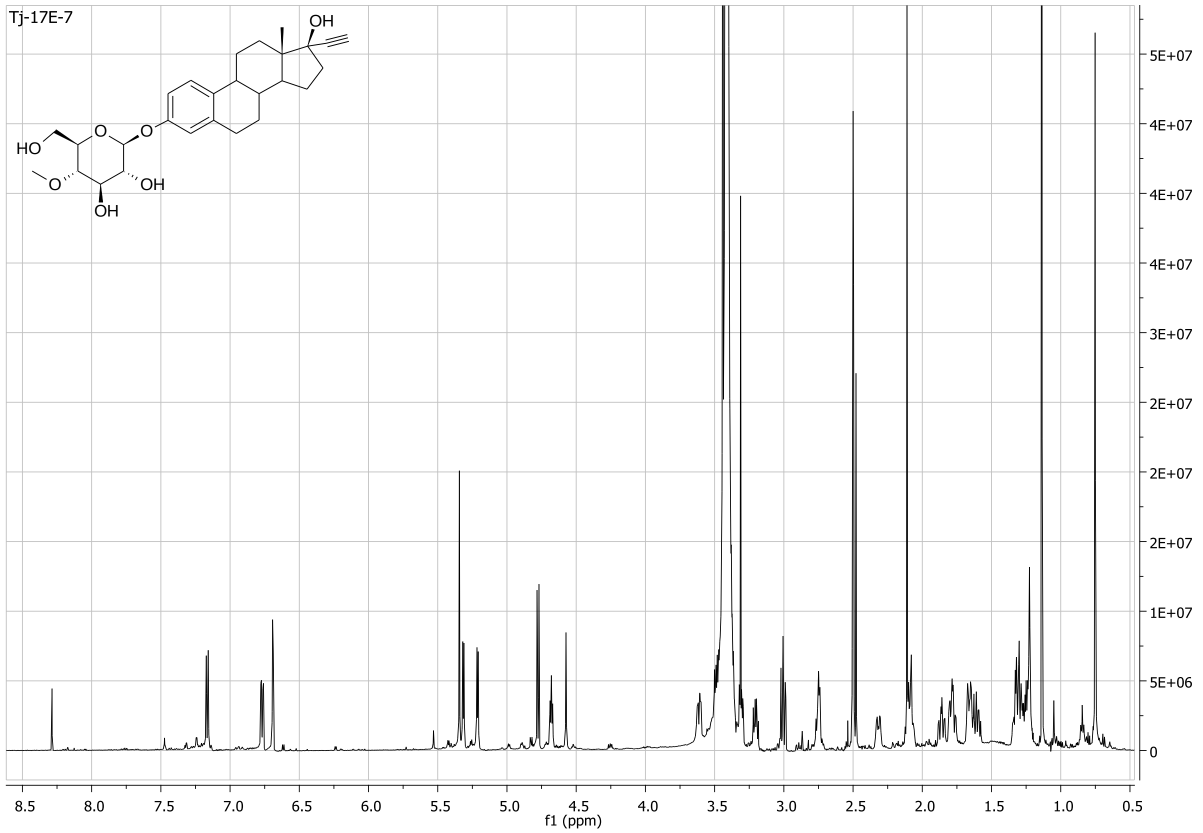

Fig.S62. ^{13}C NMR spectral of 3-(β-D-4'-O-methyloglucosyloxy)-17-ethynyloestr-17β-ol (**16**) (DMSO-*d*$_6$, 151 MHz)

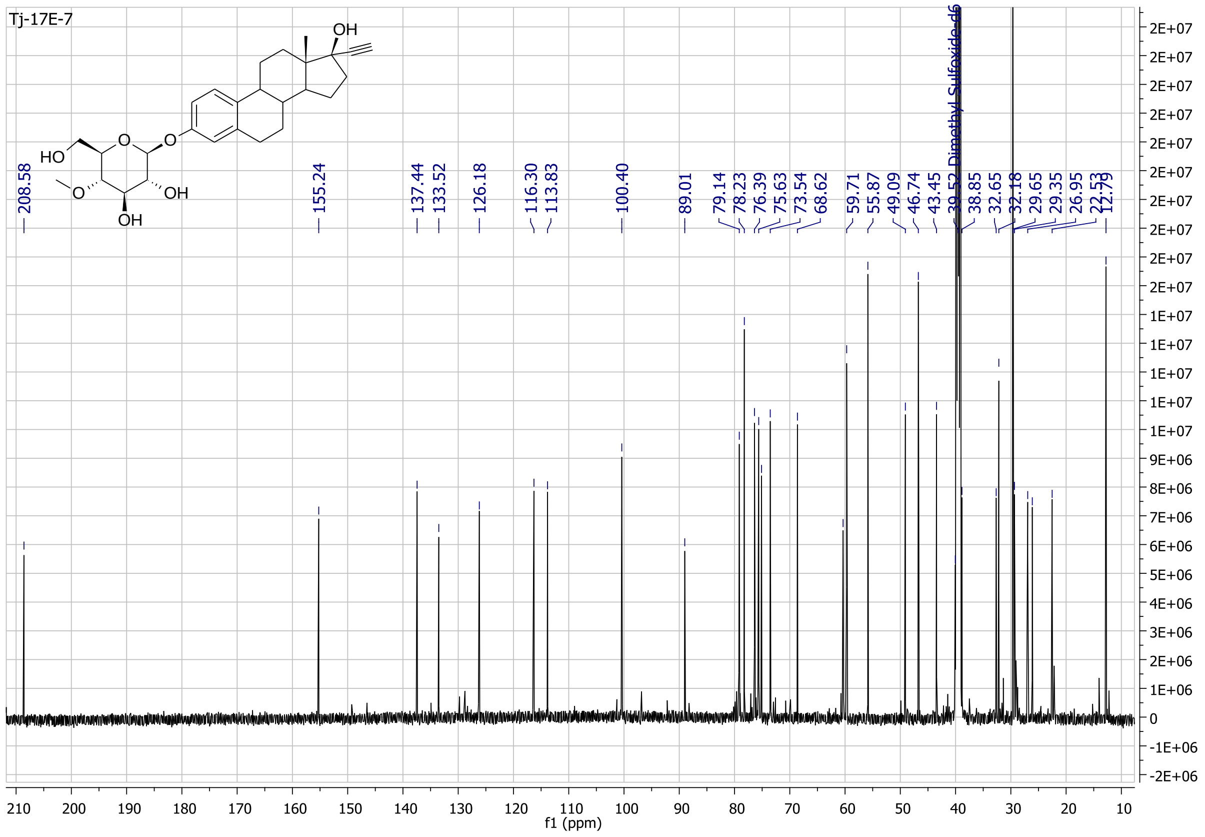

Fig.S63. HSQC spectral of 3-(β-D-4'-O-methyloglucosyloxy)-17-ethynyloestr-17β-ol (**16**) (DMSO-d_6, 151 MHz)

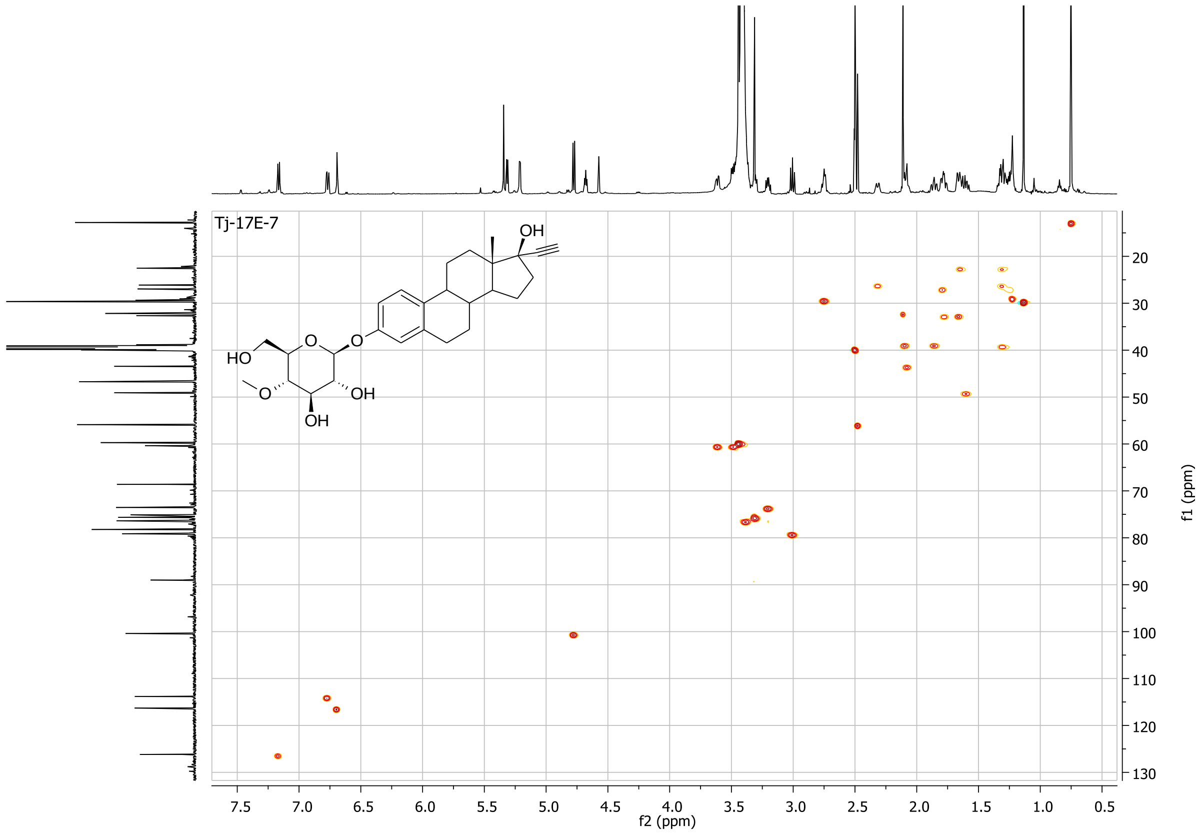

Fig.S64. COSY spectral of 3-(β-D-4'-O-methyloglucosyloxy)-17-ethynyloestr-17β-ol (**16**) (DMSO-d_6, 151 MHz)

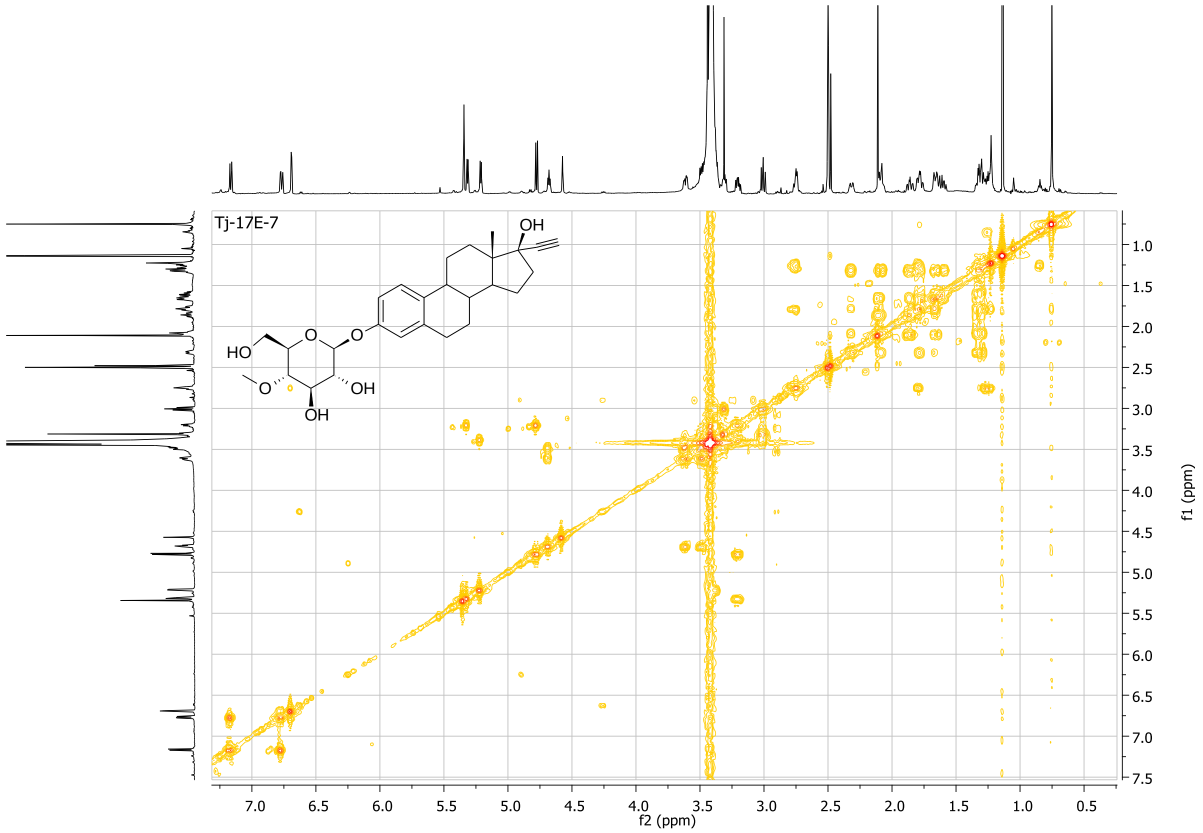

Fig.S65. HMBC spectral of 3-(β-D-4’-O-methyloglucosyloxy)-17-ethynyloestr-17β-ol (**16**) (DMSO-*d*$_6$,, 151 MHz)

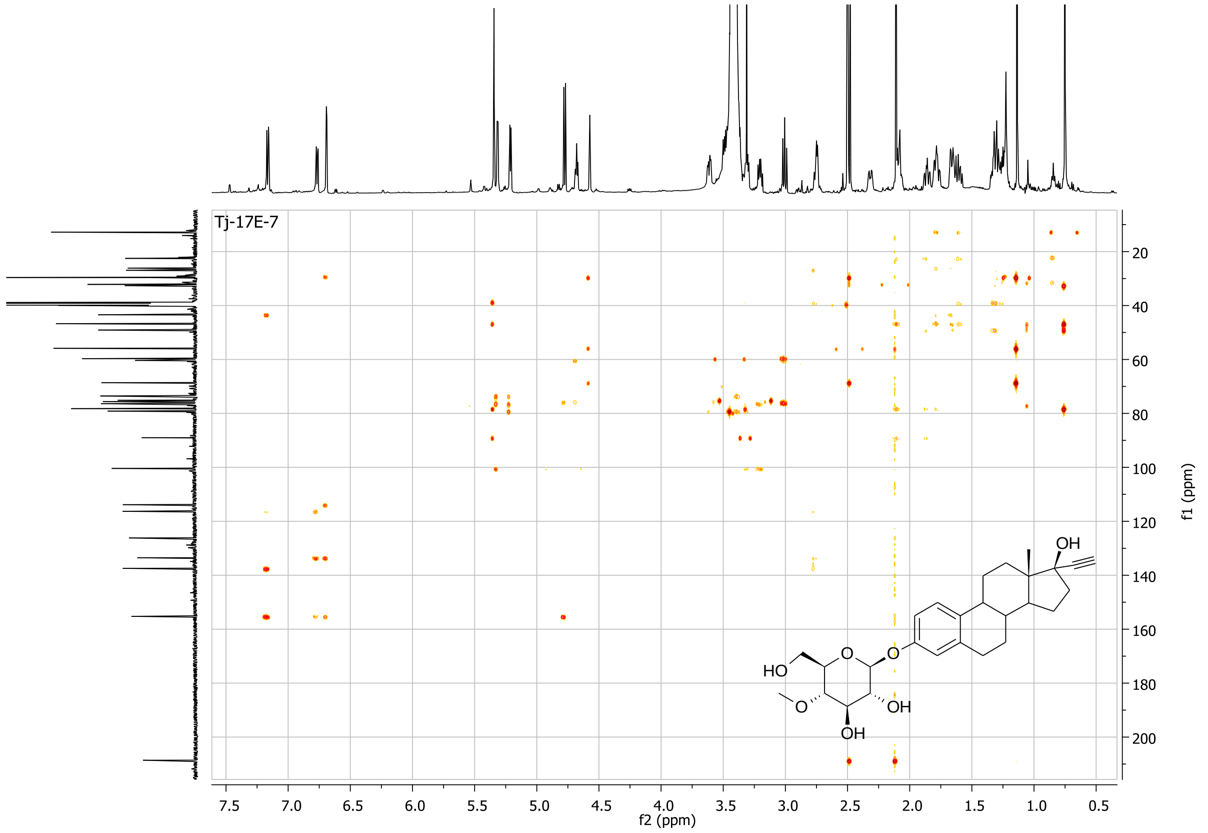

Table S1. R_f values of substrates and biotransformation products

Eluent	Compound number													
	**1**	**2**	**3**	**5**	**6**	**7**	**8**	**9**	**10**	**11**	**12**	**13**	**14**	**15**	**16**
chloroform:methanol 9:1 (*v/v*)	0,819	0,399	0,729	0,606	0,420	0,500	0,473	0,441	0,427	0,383	0,399	0,378	0,390	0,404	0,372
hexane:acetone 2:1 (*v/v*)	0,349	0,074	0,251	0,137	0,114	0,057	0,051	0,023	0,017	0,029	0,034	0,034	0,017	0,109	0,040

FigS66. TLC plates with substrates and biotransformation products (chloroform:methanol 9:1 (*v/v*))

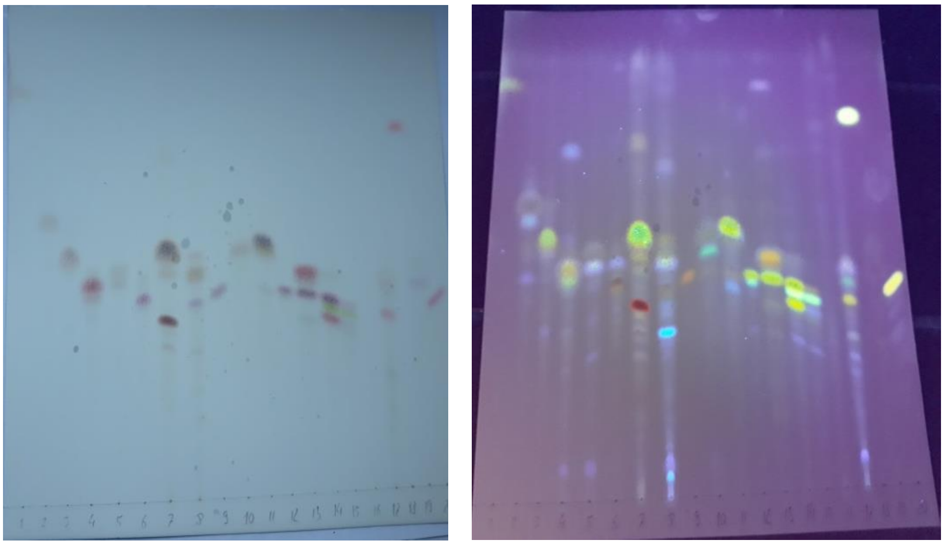

FigS67. TLC plates with substrates and biotransformation products (hexane:acetone 2:1 (*v/v*))

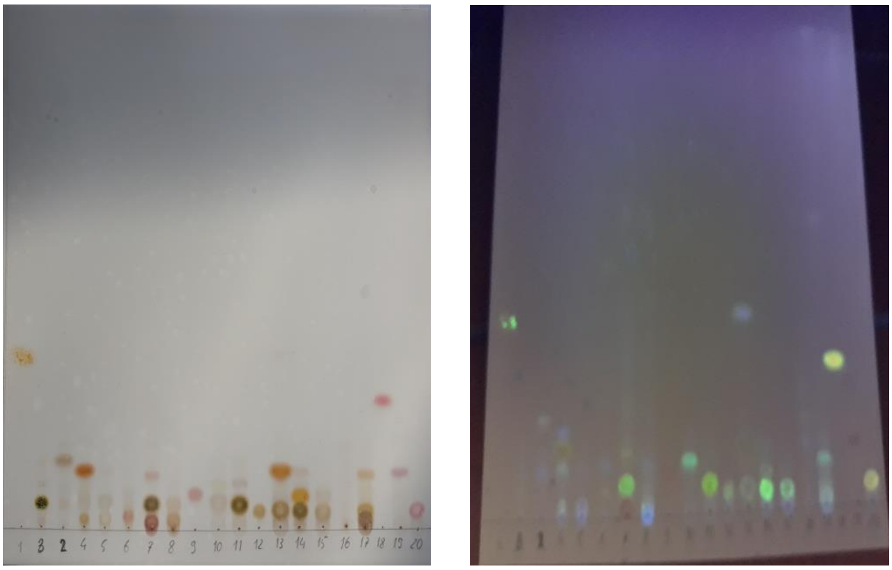

Supplement: Supplementary file 1 — Cascade biotransformation of estrogens by Isaria fumosorosea KCh J2 [file 41598_2019_47225_MOESM1_ESM.pdf]
